# Supplementary material for: Concurrent oxygen reduction and water oxidation at high ionic strength for scalable electrosynthesis of hydrogen peroxide
Source: Nat Commun. 2023 Sep 19;14:5822. doi: 10.1038/s41467-023-41397-1 (PMC10509222; doi:10.1038/s41467-023-41397-1)
Supplement: Supplementary file 1 — Supplementary information [file 41467_2023_41397_MOESM1_ESM.pdf]

Supplementary Information

Concurrent oxygen reduction and water oxidation at high ionic strength for scalable electrosynthesis of hydrogen peroxide

Changmin Kim[1], Sung O Park[2], Sang-Kyu Kwak[2,3], Zhenhai Xia[1], Guntae Kim[4,] and Liming Dai[1,*]*

[1] School of Chemical Engineering, University of New South Wales, Sydney, NSW 2052, Australia,
[2] Department of Energy Engineering, Ulsan National Institute of Science and Technology (UNIST) Ulsan, 44919, Korea,
[3] Department of Chemical and Biological Engineering, Korea University, Seoul, 02841, Korea
[4] Key Laboratory of Interfacial Physics and Technology, Shanghai Institute of Applied Physics, Chinese Academy of Sciences, Shanghai, 201800, China

**Correspondence should be addressed: gtkim@sinap.ac.cn (G.K.), l.dai@unsw.edu.au (L.D.)*

List of Contents

Supplementary Figures:

Supplementary Fig. 1 | XRD profiles of $Pr_{1.0}Sr_{1.0}Fe_{1.0-x}Zn_xO_{4-\delta}$ (x = 0, 0.25, 0.50, 0.75; denoted PSF, PSFZ25 PSFZ50, and PSFZ75) oxides. The oxides were calcined at 1150 °C air for 4 hours and ground into powder for an XRD studies. 10

Supplementary Fig. 2 | SEM images of D-PSFZ calcined at 1150 °C air. Magnification in **a** and **b** 20,000x with a scale bar of 2 μm, **c** 10,000x with a scale bar of 5 μm, and **d** 5,000x with a scale bar of 10 μm. 11

Supplementary Fig. 3 | Elemental mapping analysis *via* STEM-EDS. a STEM–EDS mapping image of D-PSFZ and **b** the corresponding the point spectrum. 12

Supplementary Fig. 4 | XRD profile of PSF oxide calcined at 1150 °C air for 4 hours. As prepared PSF oxide was ground into powder and utilised for an XRD study at a scan rate of 2° min^{-1}.........................12

Supplementary Fig. 5 | XPS profiles of PSF oxide. a Surface XPS full survey scan of PSF oxide. High-resolution surface XPS scan of **b** Pr 3*d,* **c** Sr 3*d,* **d** Fe 2*p,* **e** O 1*s* of PSF oxide..13

Supplementary Fig. 6 | XPS profiles of D-PSFZ oxide. a Surface XPS full survey scan of D-PSFZ oxide. High-resolution surface XPS scan of **b** Pr 3*d,* **c** Sr 3*d,* **d** Fe 2*p,* **e** Zn 2*p,* **f** O 1*s* of D-PSFZ oxide...........14

Supplementary Fig. 7 | XPS depth profiles of PSF oxide. XPS spectra for **a** Pr 3*d,* **b** Sr 3*d,* **c** Fe 2*p,* **d** O 1*s* regions over 22 etching levels. 15

Supplementary Fig. 8 | XPS depth profiles of D-PSFZ oxide. XPS spectra for **a** Pr 3*d,* **b** Sr 3*d,* **c** Fe 2*p,* **d** Zn 2*p,* **e** O 1*s* regions over 22 etching levels. **f** Depth profiling of O 1*s* for 4 etch levels with fittings. ..16

Supplementary Fig. 9 | BET analysis of D-PSFZ and PSF. BET surface area calculated from N_2 adsorption and desorption isotherms of **a** D-PSFZ and **c** PSF. BJH pore size distribution for **b** D-PSFZ and **d** PSF. 17

Supplementary Fig. 10 | RHE calibration profiles. Measured in H_2-saturated **a** 0.1 M, **b** 0.5 M, **c** 1.0 M, **d** 2.0 M $KHCO_3$ solution using Pt wires as a cathode and an anode and a standard calomel electrode (SCE, sat'd-KCl filled) as a reference electrode at a scan rate of 1 mV sec.$^{-1}$. 18

Supplementary Fig. 11 | RRDE polarization profiles for D-PSFZ conducted in O_2- and N_2-saturated 0.1 M $KHCO_3$. The background current profiles measured in the N_2-sat'd condition were subtracted. The solid line represents the disk current density, while the dashed line corresponds to the ring current. 19

Supplementary Fig. 12 | RRDE analysis for PSF. Studies conducted in O_2-saturated **a** 0.1 M $KHCO_3$ and **b** 0.1 M $NaHCO_3$. The background current profiles measured in the N_2-sat'd condition were subtracted. The solid line represents the disk current density, while the dashed line corresponds to the ring current. Blue scatter points indicate the corresponding H_2O_2 yield (%), and red scatter points represent the number of electrons transferred (n). 20

Supplementary Fig. 13 | RDE polarization profiles for control groups. Oxygen reduction reaction profiles at 400 to 2500 rpm in O_2-saturated 0.1 M $KHCO_3$ for **a** Pt/C, **c** PSF, and **e** D-PSFZ. The corresponding Koutechy-Levich slope analysis for **b** Pt/C, **d** PSF, and **f** D-PSFZ. 21

Supplementary Fig. 14 | RRDE analysis for Pt/C conducted in O_2-saturated 0.1 M $KHCO_3$. The solid line represents the disk current density, while the dashed line corresponds to the ring current. Blue scatter

points indicate the corresponding H_2O_2 yield (%), and red scatter points represent the number of electrons transferred (n). 22

Supplementary Fig. 15 | The ORR CA profiles of D-PSFZ. The potential ranges from 0.45 to 0.15 V measured in O_2- and N_2-saturated 0.1 M $KHCO_3$ on RRDE configuration. The background current profiles measured in the N_2-sat'd condition were subtracted. 23

Supplementary Fig. 16 | The ORR CA profiles of D-PSFZ in the $KHCO_3$ conditions. The potential ranges from 0.45 to 0.05 V obtained in O_2- **a** 0.1 M, **b** 0.5 M, **c** 1.0 M, **d** 2.0 M, and **e** saturated $KHCO_3$ on H-cell configuration and their H_2O_2 yield rate and electrons transferred number. The transferred charge and the titration amount that used for to determine H_2O_2 yields are available at **Supplementary Table 5-9.**..24

Supplementary Fig. 17 | EIS profiles of D-PSFZ at the potential range from 0.35 to 0.15 V. EIS spectra measured in O_2-saturated **a** 0.1 M, **b** 0.5 M, **c** 1.0 M, and **d** 2.0 M $KHCO_3$ on H-cell configuration. The bubbling of oxygen gas was stopped during EIS measurements....................25

Supplementary Fig. 18 | The colour mapped contour plots for the control groups. The FEs toward 2e-ORR were identified as a function of applied potentials (0.45 to 0.05 V) and solution concentrations (0.1 to 2.0 M $KHCO_3$) for **a** AB carbon black and **b** PSF oxide....................26

Supplementary Fig. 19 | XRD profiles of δ-controlled D-PSFZ oxides. δ-controlled D-PSFZ oxides are prepared by post-heating procedures and the lower or higher O*v* samples have been prepared by quenching or reducing method (denoted Q-PSFZ and R-PSFZ, respectively) as described in Methods. 27

Supplementary Fig. 20 | Digital photographs of δ-controlled D-PSFZ oxides. a Q-PSFZ (δ = 0.10-0.11): black colour; **b** D-PSFZ (δ = 0.14-0.15): dark green colour; **c** R-PSFZ (δ = 0.19-0.20): brown colour. Since the δ values are relevant to the electronic structure of the perovskite oxides, the changes of colour can be found as a function of δ values. 27

Supplementary Fig. 21 | The FEs toward 2e-ORR for δ-controlled D-PSFZ oxides (*i.e.*, Q-PSFZ (δ = 0.10–0.11); D-PSFZ (δ = 0.14–0.15); R-PSFZ (δ = 0.19–0.20)). The FEs were obtained in the O_2-sat'd 2.0 M $KHCO_3$ condition. Error bars were obtained from at least three times of measurements........28

Supplementary Fig. 22 | The ORR CA profiles of R-PSFZ in the $KHCO_3$ conditions. The potential ranges from 0.05 to 0.45 V toward 2e-ORR in **a** 0.1 M, **b** 0.5 M, **c** 1.0 M, and **d** 2.0 $KHCO_3$ on H-cell configuration. The FEs, transferred charge and the titration amount that used for to determine H_2O_2 yields are available at **Supplementary Table 10-13.** 29

Supplementary Fig. 23 | The ORR CA profiles of Q-PSFZ. The potential ranges from 0.05 to 0.45 V toward 2e-ORR in 2.0 M $KHCO_3$ on H-cell configuration. The FEs, transferred charge and the titration amount that used for to determine H_2O_2 yields are available at **Supplementary Table 14.** 30

Supplementary Fig. 24 | The ORR CA profiles of D-PSFZ. The potential ranges from 0.05 to 0.45 V toward 2e-ORR in 0.1 M KOH (pH = 13.0) on H-cell configuration. The FEs, transferred charge and the titration amount that used for to determine H_2O_2 yields are available at **Supplementary Table 15.**..........30

Supplementary Fig. 25 | UV-vis calibration spectra. 20 mM $TiOSO_4$ dissolved in 1.0 M H_2SO_4 solution was used as a coloring-reagent. Standard H_2O_2 solutions with different concentrations were prepared by diluting a 3 wt.% H_2O_2 solution (Sigma-aldrich) in 0.5 M $KHCO_3$. Standard solutions are as follows: Red: 13.2 mg L^{-1}. Yellow: 66 mg L^{-1}. Light green: 132 mg L^{-1}. Green: 198 mg L^{-1}. Blue: 264 mg L^{-1}. Violet: 330 mg L^{-1}. Each standard solutions were further diluted with addition of the $TiOSO_4$ solution. **a** 2.5x dilution (*i.e.*, 0.4 mL of H_2SO_4 + 0.4 mL of standard H_2O_2 solution + 0.2 mL of $TiOSO_4$ solution), **b** 5.0x dilution

(*i.e.*, 0.6 mL of H_2SO_4 + 0.2 mL of standard H_2O_2 solution + 0.2 mL of $TiOSO_4$ solution), **c** 10.0x dilution (*i.e.*, 0.7 mL of H_2SO_4 + 0.1 mL of standard H_2O_2 solution + 0.2 mL of $TiOSO_4$ solution). **d** Calibration fitting profiles obtained from **Supplementary Fig. 24a-24c**. The intercept value was fixed at 0.............31

Supplementary Fig. 26 | UV-vis spectra of H_2O_2 yields for R-PSFZ. H_2O_2 products were accumulated in **a** 0.1 M, **b** 0.5 M, **c** 1.0 M, **d** 2.0 M $KHCO_3$ condition. **e** UV-vis spectra of H_2O_2 yields for Q-PSFZ accumulated in 1.0 M $KHCO_3$ related with **Supplementary Fig. 22. f** UV-vis spectra of H_2O_2 yields for D-PSFZ accumulated in 0.1 M KOH (pH = 13.0) related with Supplementary Fig. 23. Each H_2O_2 yields measured from the UV-vis are shown as insets. For better comparison, the absorbance values of the titration method (the inverted triangle points, blue) were supposed to be same to that of the UV-vis method. The well-overlapping of the triangle (red) and the inverted triangle (blue) indicates the obtained H_2O_2 yields are similar. 32

Supplementary Fig. 27 | UV-vis spectra of H_2O_2 yields. H_2O_2 products were accumulated in **a** the 0.1 M and **b** the 0.5 M $KHCO_3$ condition toward ORR and the calculation of each H_2O_2 yields that measured from the UV-vis as an inset. The comparison the H_2O_2 yields obtained from the UV-vis and the titration measured in **c** the 0.1 M and **d** 0.5 M $KHCO_3$ condition. For better comparison, the absorbance values of the titration method (the inverted triangle points, blue) were supposed to be same to that of the UV-vis method. The well-overlapping of the triangle (red) and the inverted triangle (blue) indicates the obtained H_2O_2 yields are similar. 33

Supplementary Fig. 28 | The WOR CA profiles of D-PSFZ in the $KHCO_3$ conditions. The potential ranges from 2.05 to 2.35 V toward 2e-WOR and the corresponding FE and n value measured in **a** and **c** 0.1 M, **b** and **d** 0.5 M, **e** and **g** 1.0 M, and **f** and **h** 2.0 $KHCO_3$ on H-cell configuration. The transferred charge and the titration amount that used for to determine H_2O_2 yields are available at **Supplementary Table 16-19.** 34

Supplementary Fig. 29 | The WOR CA profiles of D-PSFZ in the $KHCO_3$/K_2CO_3 conditions. The potential ranges from 2.20 to 2.70 V toward 2e-WOR measured in **a** 1.0 M, and **b** 2.0 M $KHCO_3$/K_2CO_3 (pH = 10) on H-cell configuration. The FEs, transferred charge and the titration amount that used for to determine H_2O_2 yields are available at **Supplementary Table 20-21.** 35

Supplementary Fig. 30 | Fractional amount of carbonic acid, bicarbonate, and carbonate ions in various pH conditions. To utilize carbonate ions for 2e-WOR, the pH level of solution should be alkaline conditions..........36

Supplementary Fig. 31 | The ORR CA profiles of D-PSFZ in the $KHCO_3$/K_2CO_3 conditions. The potential ranges from 0.05 to 0.45 V toward 2e-ORR measured in **a** 1.0 M, and **b** 2.0 M $KHCO_3$/K_2CO_3 (pH = 10) on H-cell configuration. The transferred charge and the titration amount that used for to determine H_2O_2 yields are available at **Supplementary Table 22-23.** 37

Supplementary Fig. 32 | UV-vis spectra of H_2O_2 yields accumulated during 2e-WOR. a 1.0 M, **b** and 2.0 M $KHCO_3$/ K_2CO_3 conditions, and obtained during 2e-ORR in O_2-sat'd **c** 1.0 M, **d** and 2.0 M $KHCO_3$/ K_2CO_3 conditions. Each H_2O_2 yields measured from the UV-vis are shown as insets. For better comparison, the absorbance values of the titration method (the inverted triangle points, blue) were supposed to be same to that of the UV-vis method. The well-overlapping of the triangle (red) and the inverted triangle (blue) indicates the obtained H_2O_2 yields are similar..........38

Supplementary Fig. 33 | UV-vis spectra of H_2O_2 yields in the $KHCO_3$ conditions. H_2O_2 products were accumulated in **a** 0.1 M and **b** 0.5 M $KHCO_3$ conditions toward WOR and the calculation of each H_2O_2

yields that measured from the UV-vis as an inset. The comparison the H_2O_2 yields obtained from the UV-vis and the titration measured in **c** the 0.1 M and **d** 0.5 M $KHCO_3$ condition. For better comparison, the absorbance values of the titration method (the inverted triangle points, blue) were supposed to be same to that of the UV-vis method. The well-overlapping of the triangle (red) and the inverted triangle (blue) indicates the obtained H_2O_2 yields are similar...39

Supplementary Fig. 34 | The optimized configurations of RP-PSF and RP-PSFZ. Three different distributions of A-site ion distribution in RP-PSF were compared in terms of relative energy. Note that the relative energies were labelled below each system. Zn ion was substituted in the most stable RP-PSF framework. Blue and gray octahedra represent Fe and Zn site, while yellow, green, and red balls represent Pr, Sr, and O atoms, respectively. 40

Supplementary Fig. 35 | The optimized configurations of defective RP-PSFZ. The relative energies of eight different configurations were compared each other while the relative energies were labelled below each configuration. Blue and gray octahedra represent Fe and Zn site, while yellow, green, and red balls represent Pr, Sr, and O atoms, respectively...41

Supplementary Fig. 36 | The considered surface structures of RP-PSF, RP-PSFZ, and D-PSFZ. Half of bottom layers were constrained during the simulation. Blue, gray, yellow, green, and red balls represent Fe, Zn Pr, Sr, and O atoms, respectively. Each model was named by the information of B-site ions in the top layer and A-site ions in the second layer; Fe-Pr, Fe-Sr, FZ-Pr, FZ-Sr ('FZ' represents Fe/Zn layer). The considered active sites were labelled between top and side views of each model system. 42

Supplementary Fig. 37 | HO* and O* covered D-PSFZ structures. Half of bottom layers were constrained during the simulation. Blue, gray, yellow, green, red, and white balls represent Fe, Zn Pr, Sr, O, and H atoms, respectively. When defective Fe/Zn perovskite surface was healed by HO* (O*), the model was called as FZ-Pr/Sr-OH (FZ-Pr/Sr-O). 43

Supplementary Fig. 38 | Solution resistance values obtained in various electrolyte conditions. The D-PSFZ coated electrodes were used for the measurements in a three-electrode configuration.....................44

Supplementary Fig. 39 | Overall H_2O_2 electrolysis performance in the $KHCO_3$ condition. a Photograph of the membrane-free overall H_2O_2 electrosynthesis unit in a three-electrode configuration using the D-PSFZ as both cathode and anode (denoted as D-PSFZ || D-PSFZ) with SCE reference electrode. **b** Polarization I-V profiles of D-PSFZ || D-PSFZ measured in O_2-sat'd 0.5 M or 1.0 M $KHCO_3$. **c** Faradaic efficiency profiles obtained by the titration method with the I-V polarization curves (the upper part). The CA profiles measured in the cell voltage between 1.6 to 2.2 V (the lower part). The dashed and the solid lines indicate the electrolyte conditions are 0.5 M and 1.0 M KHCO3, respectively. The transferred charge and the titration amount that used for to determine H_2O_2 yields are available at **Supplementary Table 24-25**. 45

Supplementary Fig. 40 | The CA profiles of D-PSFZ || D-PSFZ full-cell in the $KHCO_3/K_2CO_3$ condition. The cell potential ranges from 1.70 to 2.30 V measured in 1.0 M $KHCO_3/K_2CO_3$ (pH = 10). The transferred charge and the titration amount that used for to determine H_2O_2 yields are available at **Supplementary Table 26.**..46

Supplementary Fig. 41 | UV-vis spectra of H_2O_2 yields for D-PSFZ || D-PSFZ full-cell testing accumulated in the 1.0 M $KHCO_3/K_2CO_3$ condition. Each H_2O_2 yields measured from the UV-vis are shown as insets. For better comparison, the absorbance values of the titration method (the inverted triangle points, blue) were supposed to be same to that of the UV-vis method. The well-overlapping of the triangle

(red) and the inverted triangle (blue) indicates the obtained H_2O_2 yields are similar. 47

Supplementary Fig. 42 | XRD profiles of D-PSFZ electrodes used for the stability testing of the D-PSFZ || D-PSFZ full-cell in the 2.0 M $KHCO_3/K_2CO_3$ condition. The residue of potassium carbonate hydrate solid can be found after drying of the electrolyte. 48

Supplementary Fig. 43 | SEM images of D-PSFZ electrodes used for the stability testing of the D-PSFZ || D-PSFZ full-cell in the 2.0 M $KHCO_3/K_2CO_3$ condition. a and **b** as-prepared electrode, **c** and **d** the anode after testing, **e** and **f** the cathode after testing. 49

Supplementary Fig. 44 | High-resolution surface XPS scan of D-PSFZ electrodes used for the stability testing of the D-PSFZ || D-PSFZ full-cell in the 2.0 M $KHCO_3/K_2CO_3$ condition. a, Pr 3*d* **b**, Sr 3*d* **c**, Fe 2*p* **d**, Zn 2*p* **e**, O 1*s* of D-PSFZ electrodes. 50

Supplementary Fig. 45 | XPS depth profiles of the as-prepared D-PSFZ electrode. XPS spectra for **a** Pr 3*d,* **b** Sr 3*d,* **c** Fe 2*p,* **d** Zn 2*p,* **e** O 1*s* regions over 10 etching levels. 51

Supplementary Fig. 46 | XPS depth profiles of the D-PSFZ anode after the full-cell testing. XPS spectra for **a** Pr 3*d,* **b** Sr 3*d,* **c** Fe 2*p,* **d** Zn 2*p,* **e** O 1*s* regions over 10 etching levels. 52

Supplementary Fig. 47 | XPS depth profiles of the D-PSFZ cathode after the full-cell testing. XPS spectra for **a** Pr 3*d,* **b** Sr 3*d,* **c** Fe 2*p,* **d** Zn 2*p,* **e** O 1*s* regions over 10 etching levels. 53

Supplementary Fig. 48 | HR-TEM images of the D-PSFZ electrodes that used for the stability testing of the D-PSFZ || D-PSFZ full-cell in the 2.0 M $KHCO_3/K_2CO_3$ condition. a and **b** HR-TEM images of the anode and enlarged images as insets. **d** and **e** HR-TEM images of the cathode and an enlarged image as an inset. **c** and **f** Schematic models of RP-perovskite supercells ($A_{16}O_8O_{32}$) aligned with the zone axis of [331] and [100] respectively. 54

Supplementary Note 1 | Calculation details for the FE of the D-PSFZ || D-PSFZ cell of Supplementary Movie 1 55

Supplementary Tables:

Supplementary Table 1 | Summary of point spectrum in Supplementary Fig. 3. 56

Supplementary Table 2 | Lattice parameters of D-PSFZ calculated by Rietveld refinement. 56

Supplementary Table 3 | XPS peak deconvolution results of PSF oxide based on the relative area of each deconvolution peaks shown in **Supplementary Fig. 4**. Note Sr cation has the fixed transition state as 2+. 56

Supplementary Table 4 | XPS peak deconvolution results of D-PSFZ oxide based on the relative area of each deconvolution peaks shown in **Supplementary Fig. 5**. Note Sr and Zn cations have the fixed transition states as 2+. 56

Supplementary Table 5 | Data sheet of the permanganate titration to calculate H_2O_2 yield and Faradaic efficiency (%) during 2e-ORR in 0.1 M $KHCO_3$(aq) for D-PSFZ. The H_2O_2 concentration was accumulated by chronoamperometric measurement for 10 min at various potential range between 0.05 to 0.45 V *vs.* RHE (For the titration, 0.5 mL of the product solution was taken). 57

Supplementary Table 6 | Data sheet of the permanganate titration to calculate H_2O_2 yield and Faradaic

efficiency (%) during 2e-ORR in 0.5 M $KHCO_3$(aq) for D-PSFZ. The H_2O_2 concentration was accumulated by chronoamperometric measurement for 15 min at various potential range between 0.05 to 0.45 V *vs.* RHE (For the titration, 0.3 mL of the product solution was taken). 57

Supplementary Table 7 | Data sheet of the permanganate titration to calculate H_2O_2 yield and Faradaic efficiency (%) during 2e-ORR in 1.0 M $KHCO_3$(aq) for D-PSFZ. The H_2O_2 concentration was accumulated by chronoamperometric measurement for 10 min at various potential range between 0.05 to 0.45 V *vs.* RHE (For the titration, 0.5 mL of the product solution was taken). 58

Supplementary Table 8 | Data sheet of the permanganate titration to calculate H_2O_2 yield and Faradaic efficiency (%) during 2e-ORR in 2.0 M $KHCO_3$(aq) for D-PSFZ. The H_2O_2 concentration was accumulated by chronoamperometric measurement for 10 min at various potential range between 0.05 to 0.45 V *vs.* RHE (For the titration, 0.3 mL of the product solution was taken, *for the 0.05 V condition, 0.2 mL of the solution was taken). 58

Supplementary Table 9 | Data sheet of the permanganate titration to calculate H_2O_2 yield and Faradaic efficiency (%) during 2e-ORR in saturated $KHCO_3$(aq) for D-PSFZ. The H_2O_2 concentration was accumulated by chronoamperometric measurement for 10 min at various potential range between 0.05 to 0.45 V *vs.* RHE (For the titration, 0.3 mL of the product solution was taken). 59

Supplementary Table 10 | Data sheet of the permanganate titration to calculate H_2O_2 yield and Faradaic efficiency (%) during 2e-ORR in 0.1 M $KHCO_3$(aq) for R-PSFZ. (Electrolyte: 12 mL, for the titration, 0.5 mL of the product solution was taken). 59

Supplementary Table 11 | Data sheet of the permanganate titration to calculate H_2O_2 yield and Faradaic efficiency (%) during 2e-ORR in 0.5 M $KHCO_3$(aq) for R-PSFZ. (Electrolyte: 12 mL, for the titration, 0.5 mL of the product solution was taken). 60

Supplementary Table 12 | Data sheet of the permanganate titration to calculate H_2O_2 yield and Faradaic efficiency (%) during 2e-ORR in 1.0 M $KHCO_3$(aq) for R-PSFZ. (Electrolyte: 12 mL, for the titration, 0.5 mL of the product solution was taken). 60

Supplementary Table 13 | Data sheet of the permanganate titration to calculate H_2O_2 yield and Faradaic efficiency (%) during 2e-ORR in 2.0 M $KHCO_3$(aq) for R-PSFZ. (Electrolyte: 12 mL, for the titration, 0.5 mL of the product solution was taken). 61

Supplementary Table 14 | Data sheet of the permanganate titration to calculate H_2O_2 yield and Faradaic efficiency (%) during 2e-ORR in 2.0 M $KHCO_3$(aq) for Q-PSFZ. (Electrolyte: 12 mL, for the titration, 0.5 mL of the product solution was taken). 61

Supplementary Table 15 | Data sheet of the permanganate titration to calculate H_2O_2 yield and Faradaic efficiency (%) during 2e-ORR in 0.1 M KOH(aq) for D-PSFZ. (Electrolyte: 12 mL, for the titration, 0.5 mL of the product solution was taken). 62

Supplementary Table 16 | Data sheet of the permanganate titration to calculate H_2O_2 yield and Faradaic efficiency (%) during 2e-WOR in 0.1 M $KHCO_3$(aq) for D-PSFZ. The H_2O_2 concentration was accumulated by chronoamperometric measurement for 30 min at various potential range between 0.05 to 0.45 V *vs.* RHE (For the titration, 0.5 mL of the product solution was taken). 62

Supplementary Table 17 | Data sheet of the permanganate titration to calculate H_2O_2 yield and Faradaic efficiency (%) during 2e-WOR in 0.5 M $KHCO_3$(aq) for D-PSFZ. The H_2O_2 concentration was accumulated by chronoamperometric measurement for 30 min at various potential range between 0.05 to 0.45 V *vs.* RHE

(For the titration, 0.5 mL of the product solution was taken). 63

Supplementary Table 18 | Data sheet of the permanganate titration to calculate H_2O_2 yield and Faradaic efficiency (%) during 2e-WOR in 1.0 M $KHCO_3$(aq) for D-PSFZ. The H_2O_2 concentration was accumulated by chronoamperometric measurement for 30 min at various potential range between 0.05 to 0.45 V *vs.* RHE (For the titration, 0.5 mL of the product solution was taken). 63

Supplementary Table 19 | Data sheet of the permanganate titration to calculate H_2O_2 yield and Faradaic efficiency (%) during 2e-WOR in 2.0 M $KHCO_3$(aq) for D-PSFZ. The H_2O_2 concentration was accumulated by chronoamperometric measurement for 15 min at various potential range between 0.05 to 0.45 V *vs.* RHE (For the titration, 0.5 mL of the product solution was taken). 64

Supplementary Table 20 | Data sheet of the permanganate titration to calculate H_2O_2 yield and Faradaic efficiency (%) during 2e-WOR in 1.0 M $KHCO_3/K_2CO_3$(aq) for D-PSFZ. (Electrolyte: 90 mL, for the titration, 0.5 mL of the product solution was taken). 64

Supplementary Table 21 | Data sheet of the permanganate titration to calculate H_2O_2 yield and Faradaic efficiency (%) during 2e-WOR in 2.0 M $KHCO_3/K_2CO_3$(aq) for D-PSFZ. (Electrolyte: 75 mL, for the titration, 0.5 mL of the product solution was taken, *only for 2.10 V, 1.0 mL of the solution was taken to avoid error) 65

Supplementary Table 22 | Data sheet of the permanganate titration to calculate H_2O_2 yield and Faradaic efficiency (%) during 2e-ORR in 1.0 M $KHCO_3/K_2CO_3$(aq) for D-PSFZ. (Electrolyte: 12 mL, for the titration, 0.5 mL of the product solution was taken). 65

Supplementary Table 23 | Data sheet of the permanganate titration to calculate H_2O_2 yield and Faradaic efficiency (%) during 2e-ORR in 2.0 M $KHCO_3/K_2CO_3$(aq) for D-PSFZ. (Electrolyte: 12 mL, for the titration, 0.5 mL of the product solution was taken) 66

Supplementary Table 24 | Data sheet of the permanganate titration to calculate H_2O_2 yield and Faradaic efficiency (%) during the overall H_2O_2 electrolysis in 0.5 M $KHCO_3$(aq) for D-PSFZ || D-PSFZ. The H_2O_2 concentration was accumulated by chronoamperometric measurement for 10 min at the cell potential range between 1.60 to 2.20 V and 16 mL of electrolyte was used (For the titration, 0.5 mL of the product solution was taken, *only for 1.60 V, 0.4 mL of the solution was taken.). 66

Supplementary Table 25 | Data sheet of the permanganate titration to calculate H_2O_2 yield and Faradaic efficiency (%) during the overall H_2O_2 electrolysis in 1.0 M $KHCO_3$(aq) for D-PSFZ || D-PSFZ. The H_2O_2 concentration was accumulated by chronoamperometric measurement for 10 min at the cell potential range between 1.60 to 2.20 V and 16 mL of electrolyte was used (For the titration, 0.2 mL of the product solution was taken, *only for 1.60 V, 0.4 mL of the solution was taken.). 67

Supplementary Table 26 | Data sheet of the permanganate titration to calculate H_2O_2 yield and Faradaic efficiency (%) during the overall H_2O_2 electrolysis in 1.0 M $KHCO_3/K_2CO_3$(aq) for D-PSFZ || D-PSFZ. (Electrolyte: 12 mL, for the titration, 0.5 mL of the product solution was taken) 67

Supplementary Table 27 | Data sheet of the permanganate titration to calculate H_2O_2 yield and Faradaic efficiency (%) during the stability test toward 2e-ORR in 1.0 M $KHCO_3$(aq) for D-PSFZ shown in **Figure 3d**. (Electrolyte flow rate: 10 sccm, for the titration, 0.5 mL of the product solution was taken) 68

Supplementary Table 28 | Data sheet of the permanganate titration to calculate H_2O_2 yield and Faradaic efficiency (%) during the stability test toward 2e-WOR in 1.0 M $KHCO_3/K_2CO_3$(aq) for D-PSFZ shown in **Figure 4d**. (Electrolyte flow rate: 15 sccm, chronopotentiometry at 70 mA cm^{-2} on 1 cm^2 electrode) 69

Supplementary Table 29 | Data sheet of the permanganate titration to calculate H_2O_2 yield and Faradaic efficiency (%) during the stability test toward the overall H_2O_2 electrosynthesis in 2.0 M $KHCO_3/K_2CO_3$(aq) shown in **Figure 6c**. (Electrolyte flow rate: 15 sccm, at 50 mA cm^{-2} on 1 cm^2 electrodes, cathode FE = 95 %) 69

Supplementary Table 30 | Comparison of 2e-ORR activities of D-PSFZ to recently published articles measured at high η region. [a] η = 0.6 V indicates where E = 0.10 V *vs.* RHE since $E^o(O_2/H_2O_2)$ = 0.70 V. 70

Supplementary Table 31 | Comparison of 2e-WOR activities of D-PSFZ to recently published articles measured at high η region. [a] η = 0.6 V indicates where E = ~2.35 V *vs.* RHE since $E^o(H_2O_2/H_2O)$ = 1.76 V. 73

Supplementary Table 32 | Concurrent H_2O_2 production electrochemical cells and their performance. [a]The active areas of cathode and anode are different thus the current density (J) are shown in J_{anode} and $J_{cathode}$. [b]The FEs are not available. [c]The system is based on the photo-electrocatalysis. 74

Supplementary References. 74

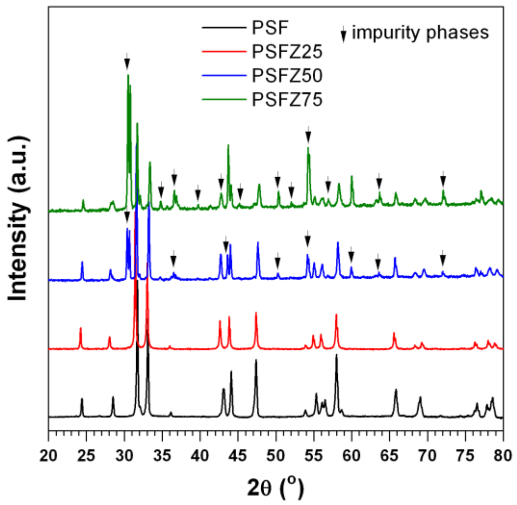

Supplementary Fig. 1 | XRD profiles of $Pr_{1.0}Sr_{1.0}Fe_{1.0-x}Zn_xO_{4-\delta}$ (x = 0, 0.25, 0.50, 0.75; denoted PSF, PSFZ25 PSFZ50, and PSFZ75) oxides. The oxides were calcined at 1150 °C air for 4 hours and ground into powder for an XRD studies.

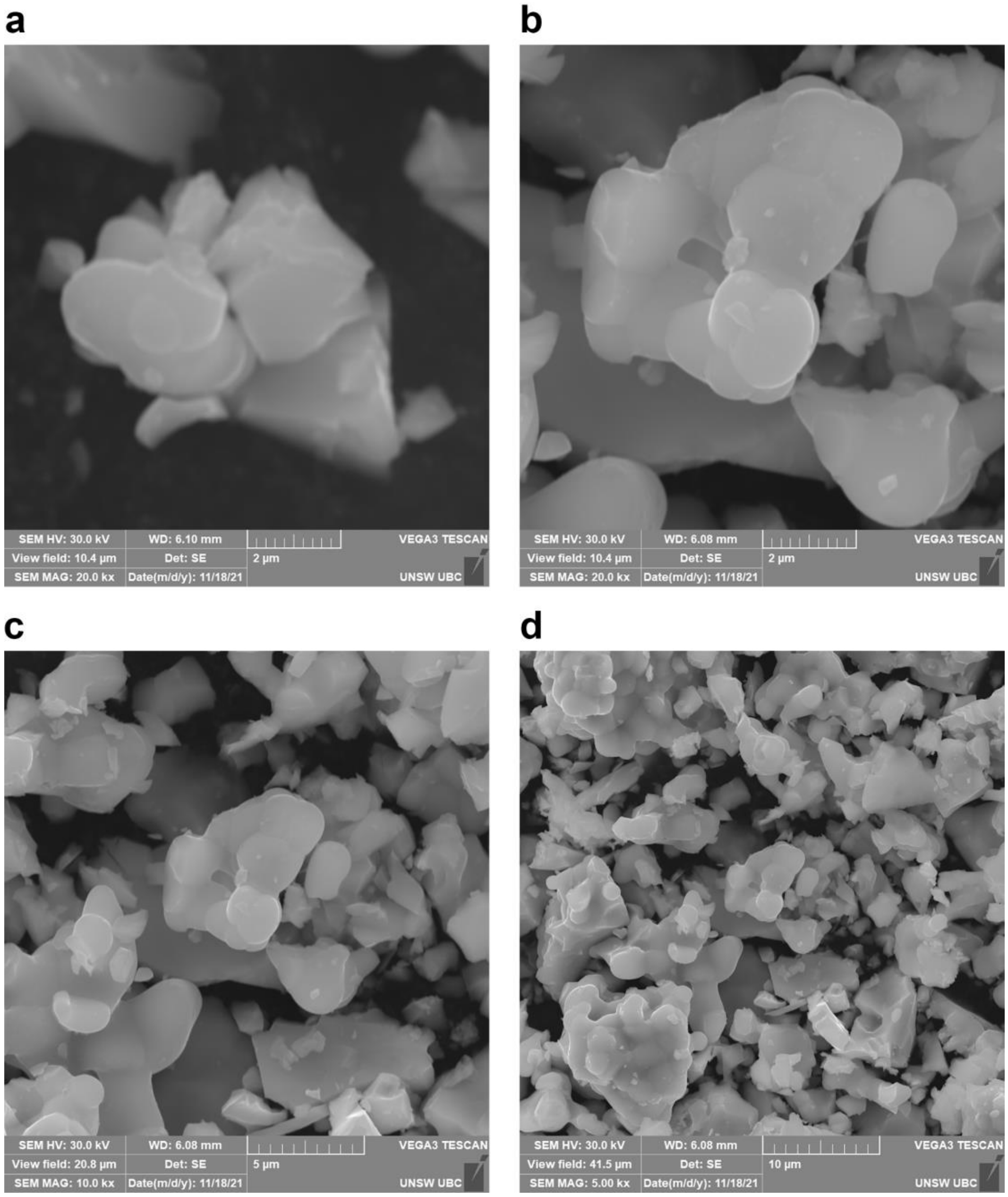

Supplementary Fig. 2 | SEM images of D-PSFZ calcined at 1150 ºC air. Magnification in **a** and **b** 20,000x with a scale bar of 2 μm, **c** 10,000x with a scale bar of 5 μm, and **d** 5,000x with a scale bar of 10 μm.

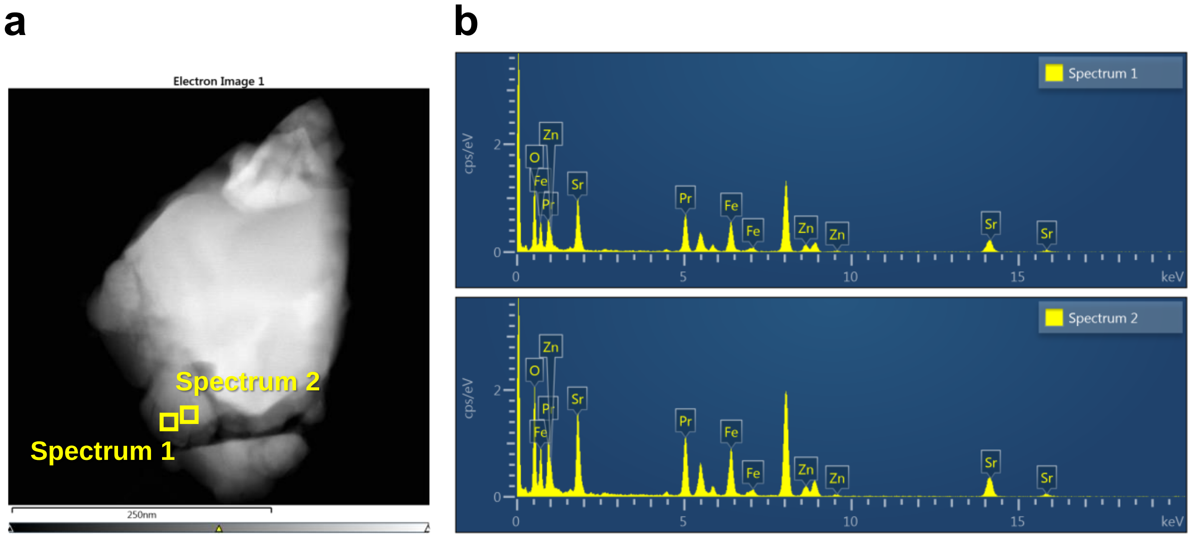

Supplementary Fig. 3 | Elemental mapping analysis *via* STEM-EDS. a STEM–EDS mapping image of D-PSFZ and **b** the corresponding the point spectrum.

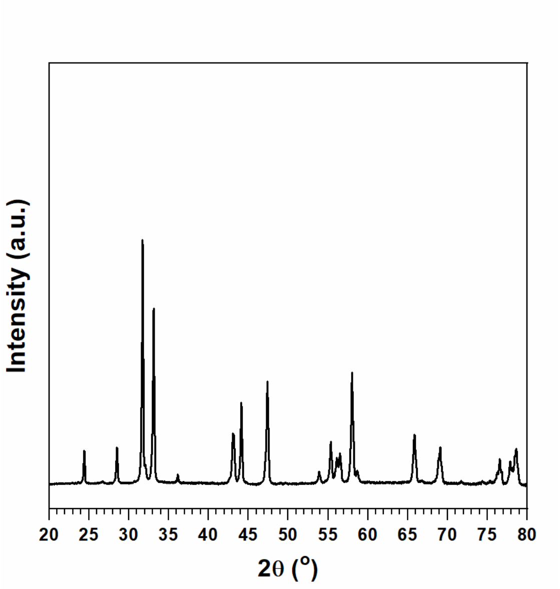

Supplementary Fig. 4 | XRD profile of PSF oxide calcined at 1150 °C air for 4 hours. As prepared PSF oxide was ground into powder and utilised for an XRD study at a scan rate of 2° min^{-1}.

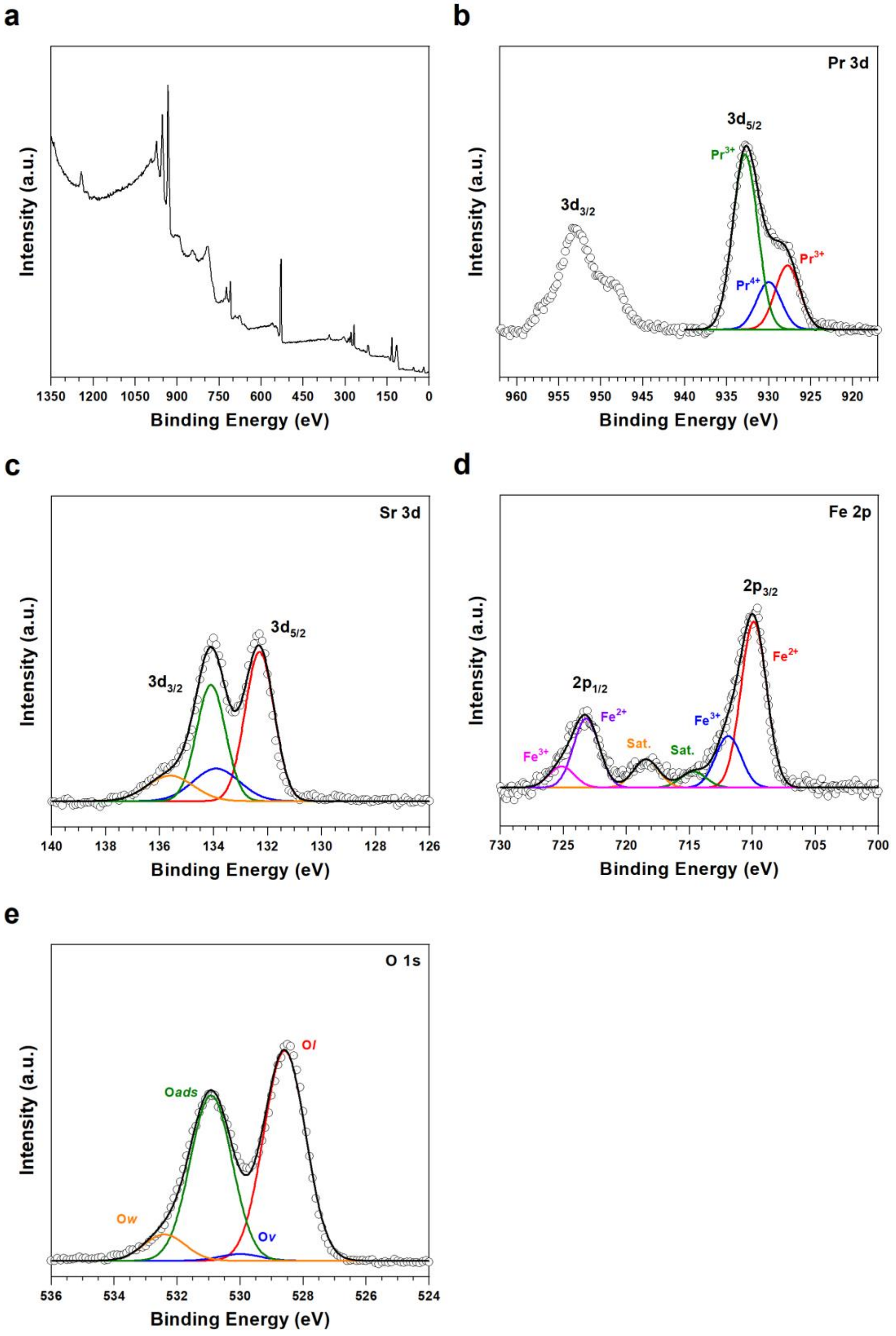

Supplementary Fig. 5 | XPS profiles of PSF oxide. a Surface XPS full survey scan of PSF oxide. High-resolution surface XPS scan of **b** Pr 3*d*, **c** Sr 3*d*, **d** Fe 2*p*, **e** O 1*s* of PSF oxide.

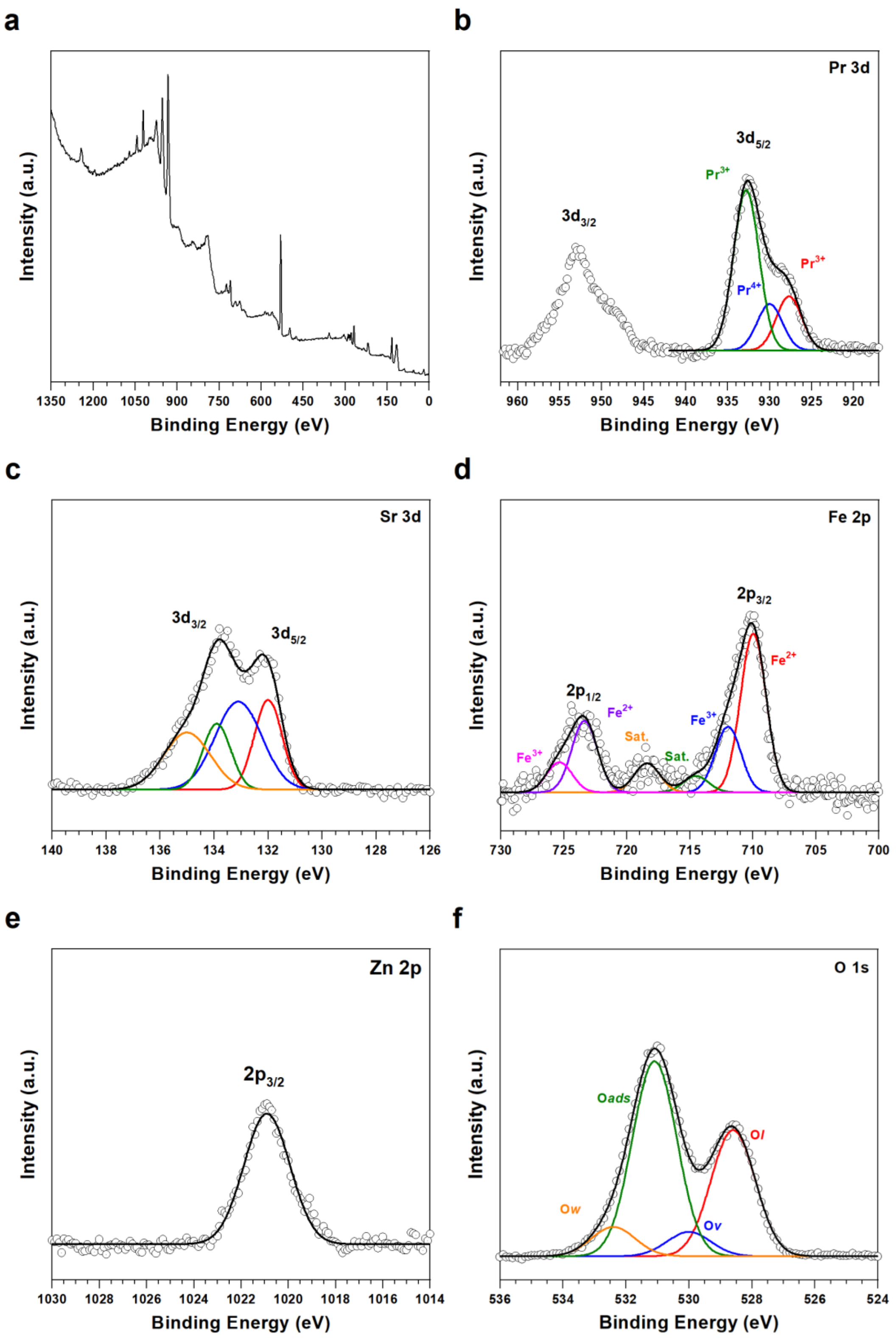

Supplementary Fig. 6 | XPS profiles of D-PSFZ oxide. a Surface XPS full survey scan of D-PSFZ oxide. High-resolution surface XPS scan of **b** Pr 3*d*, **c** Sr 3*d*, **d** Fe 2*p*, **e** Zn 2*p*, **f** O 1*s* of D-PSFZ oxide.

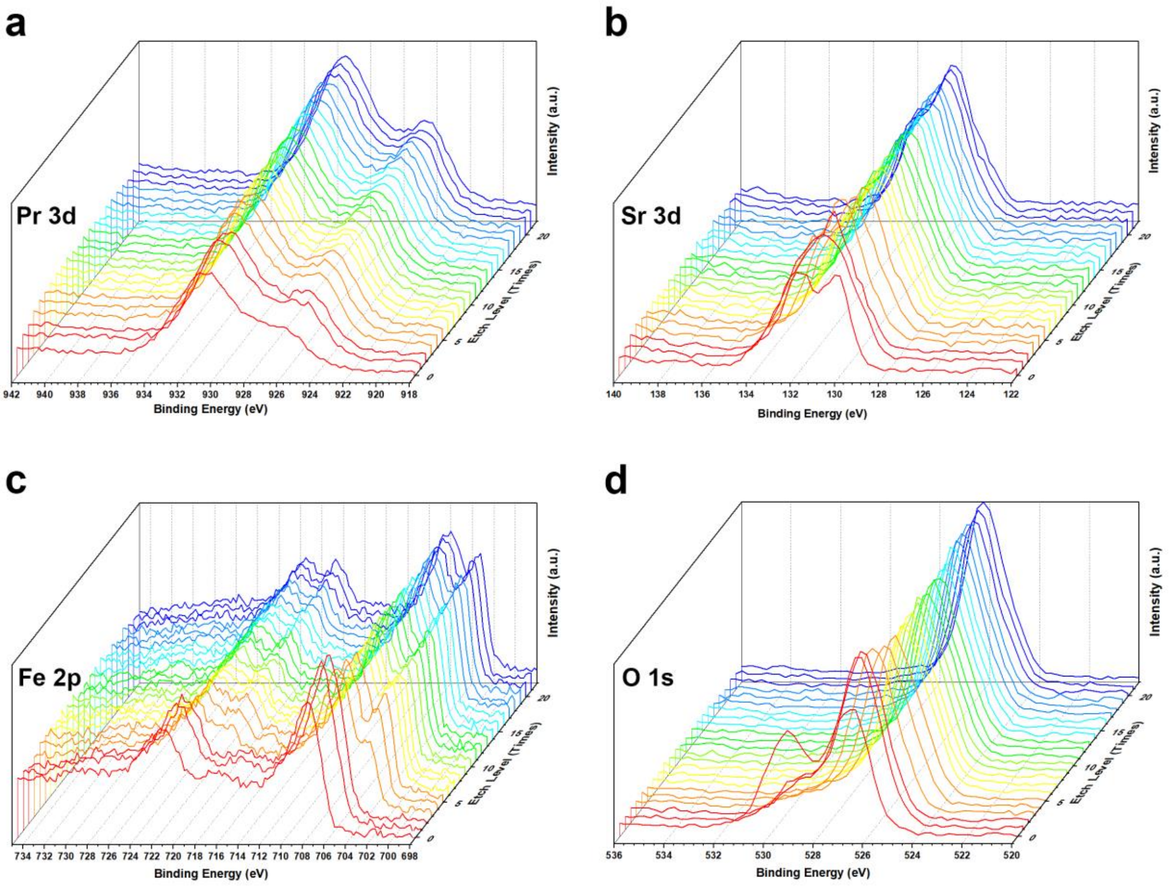

Supplementary Fig. 7 | XPS depth profiles of PSF oxide. XPS spectra for **a** Pr 3*d*, **b** Sr 3*d*, **c** Fe 2*p*, **d** O 1*s* regions over 22 etching levels.

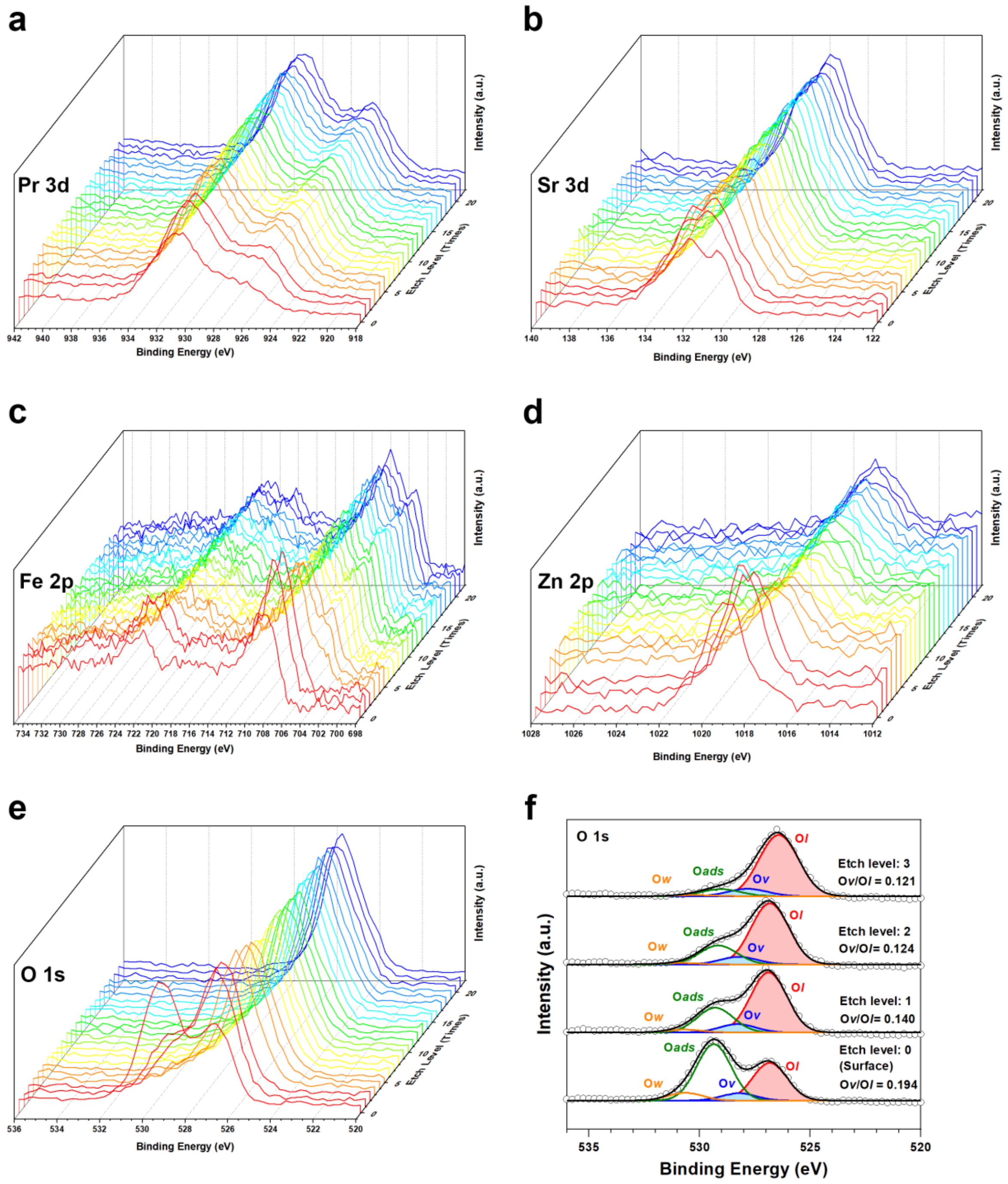

Supplementary Fig. 8 | XPS depth profiles of D-PSFZ oxide. XPS spectra for **a** Pr 3*d*, **b** Sr 3*d*, **c** Fe 2*p*, **d** Zn 2*p*, **e** O 1*s* regions over 22 etching levels. **f** Depth profiling of O 1*s* for 4 etch levels with fittings.

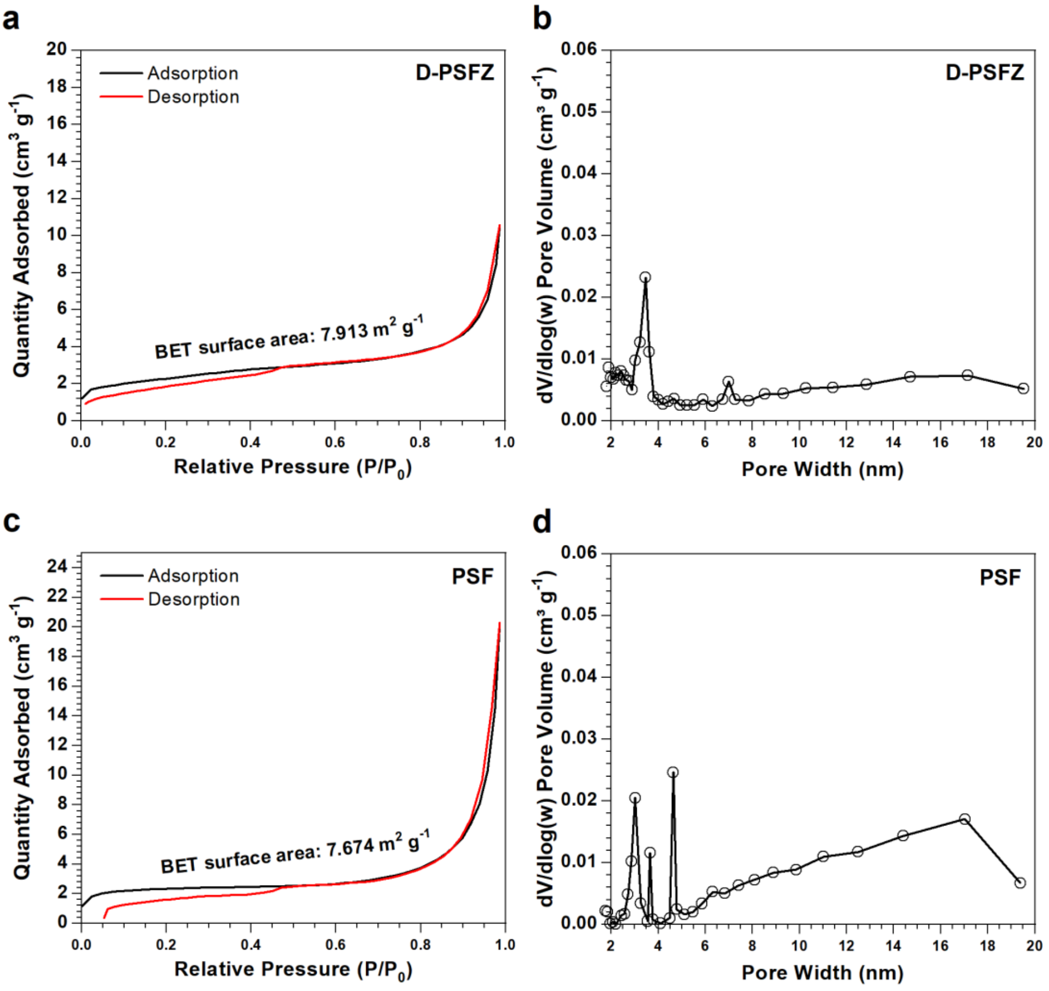

Supplementary Fig. 9 | BET analysis of D-PSFZ and PSF. BET surface area calculated from N_2 adsorption and desorption isotherms of **a** D-PSFZ and **c** PSF. BJH pore size distribution for **b** D-PSFZ and **d** PSF.

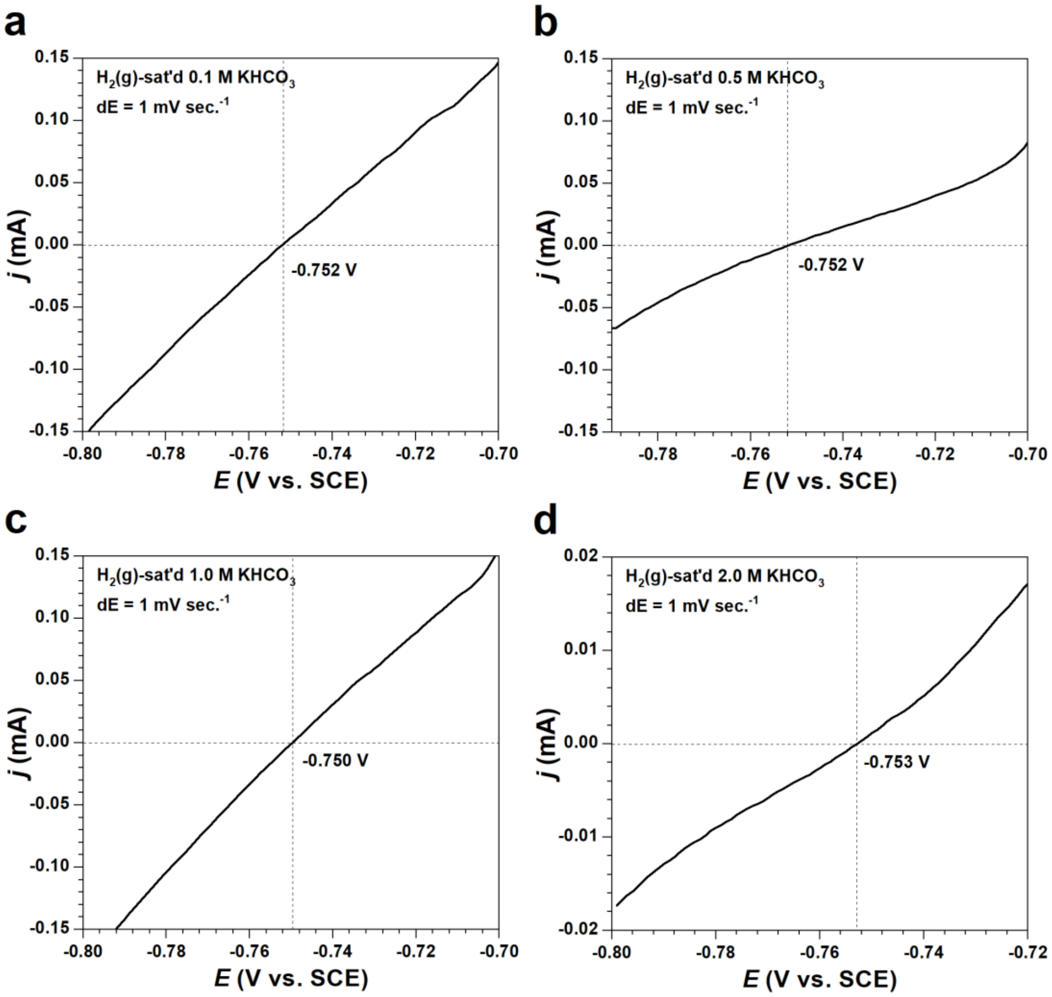

Supplementary Fig. 10 | RHE calibration profiles. Measured in H_2-saturated **a** 0.1 M, **b** 0.5 M, **c** 1.0 M, **d** 2.0 M $KHCO_3$ solution using Pt wires as a cathode and an anode and a standard calomel electrode (SCE, sat'd-KCl filled) as a reference electrode at a scan rate of 1 mV sec.$^{-1}$.

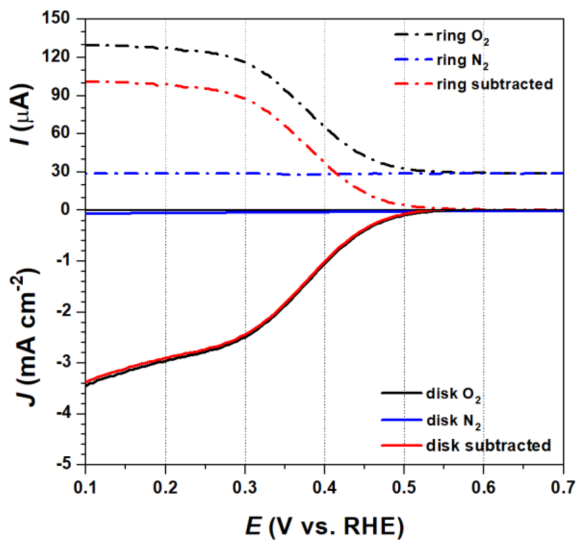

Supplementary Fig. 11 | RRDE polarization profiles for D-PSFZ conducted in O_2- and N_2-saturated 0.1 M $KHCO_3$. The background current profiles measured in the N_2-sat'd condition were subtracted. The solid line represents the disk current density, while the dashed line corresponds to the ring current.

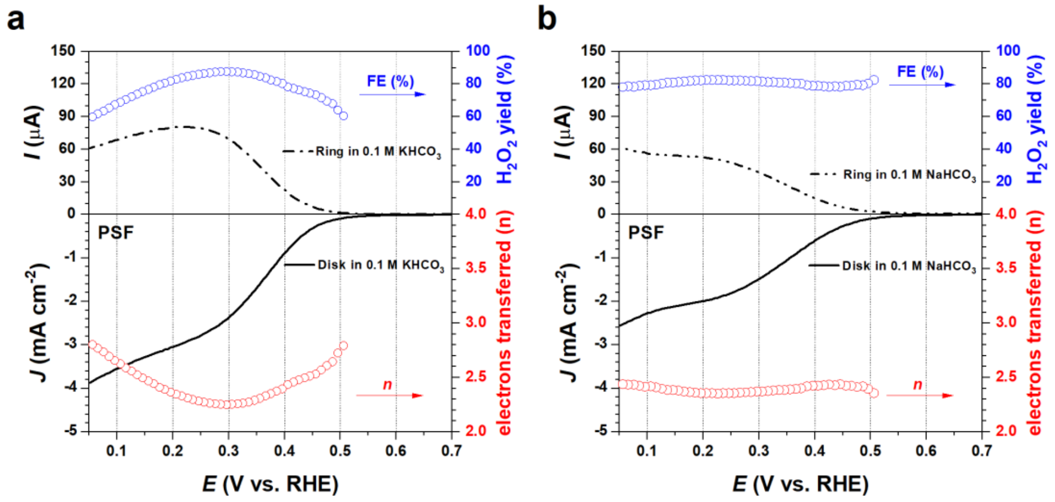

Supplementary Fig. 12 | RRDE analysis for PSF. Studies conducted in O_2-saturated **a** 0.1 M $KHCO_3$ and **b** 0.1 M $NaHCO_3$. The background current profiles measured in the N_2-sat'd condition were subtracted. The solid line represents the disk current density, while the dashed line corresponds to the ring current. Blue scatter points indicate the corresponding H_2O_2 yield (%), and red scatter points represent the number of electrons transferred (n).

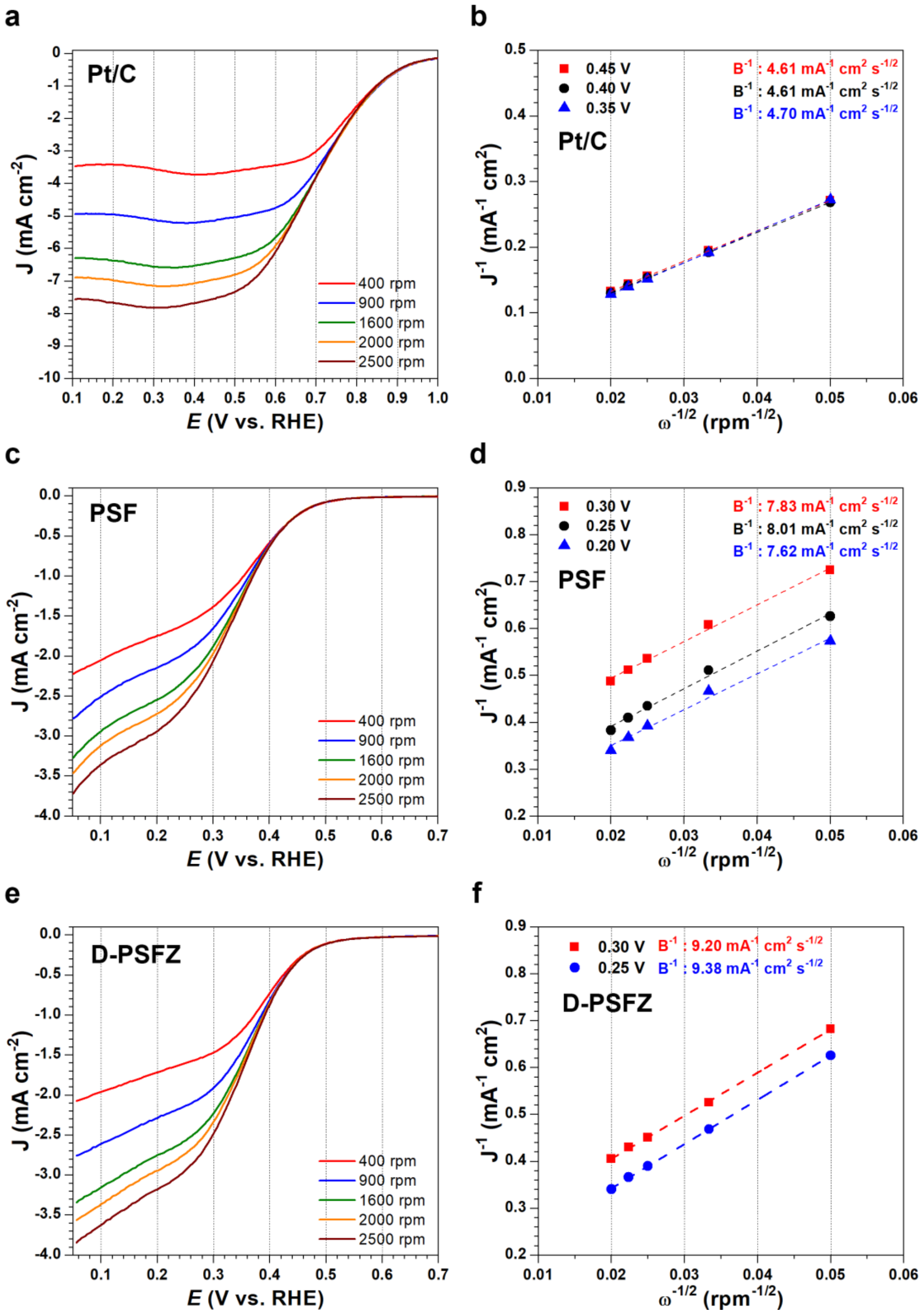

Supplementary Fig. 13 | RDE polarization profiles for control groups. Oxygen reduction reaction profiles at 400 to 2500 rpm in O_2-saturated 0.1 M $KHCO_3$ for **a** Pt/C, **c** PSF, and **e** D-PSFZ. The corresponding Koutechy-Levich slope analysis for **b** Pt/C, **d** PSF, and **f** D-PSFZ.

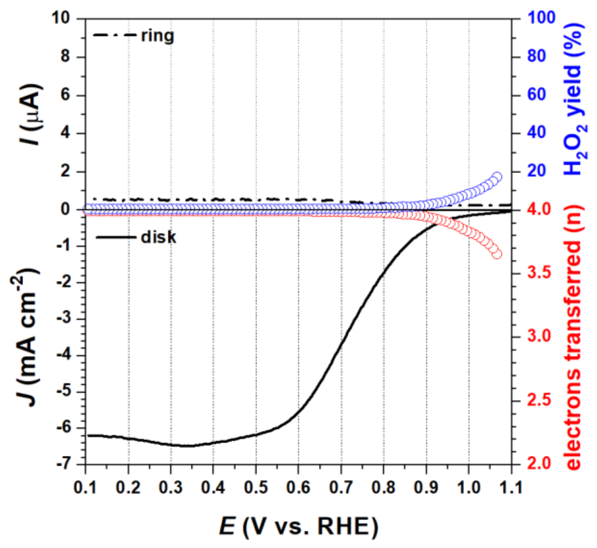

Supplementary Fig. 14 | RRDE analysis for Pt/C conducted in O_2-saturated 0.1 M $KHCO_3$. The solid line represents the disk current density, while the dashed line corresponds to the ring current. Blue scatter points indicate the corresponding H_2O_2 yield (%), and red scatter points represent the number of electrons transferred (n).

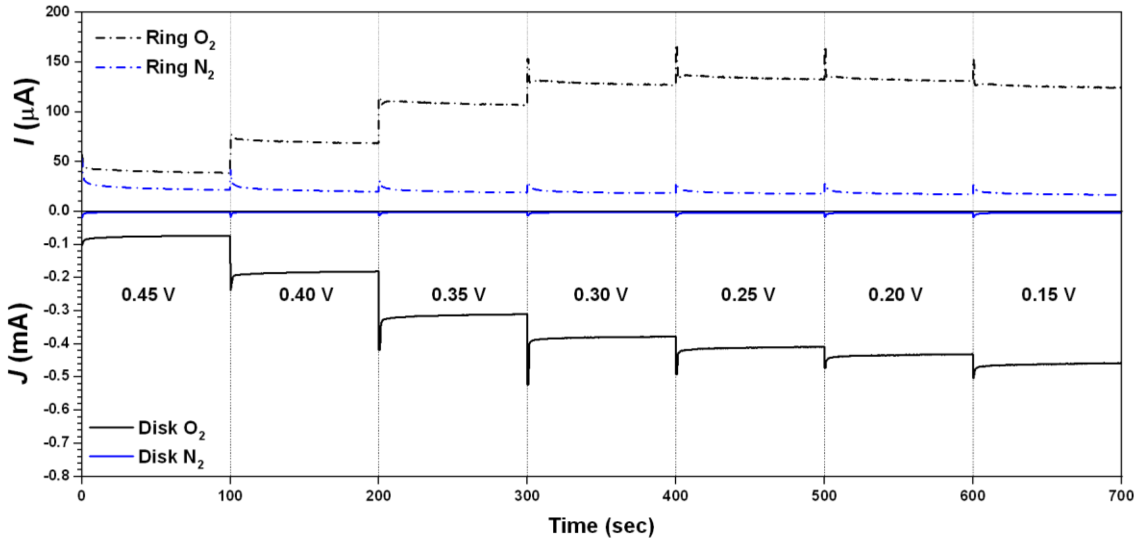

Supplementary Fig. 15 | The ORR CA profiles of D-PSFZ. The potential ranges from 0.45 to 0.15 V measured in O_2- and N_2-saturated 0.1 M $KHCO_3$ on RRDE configuration. The background current profiles measured in the N_2-sat'd condition were subtracted.

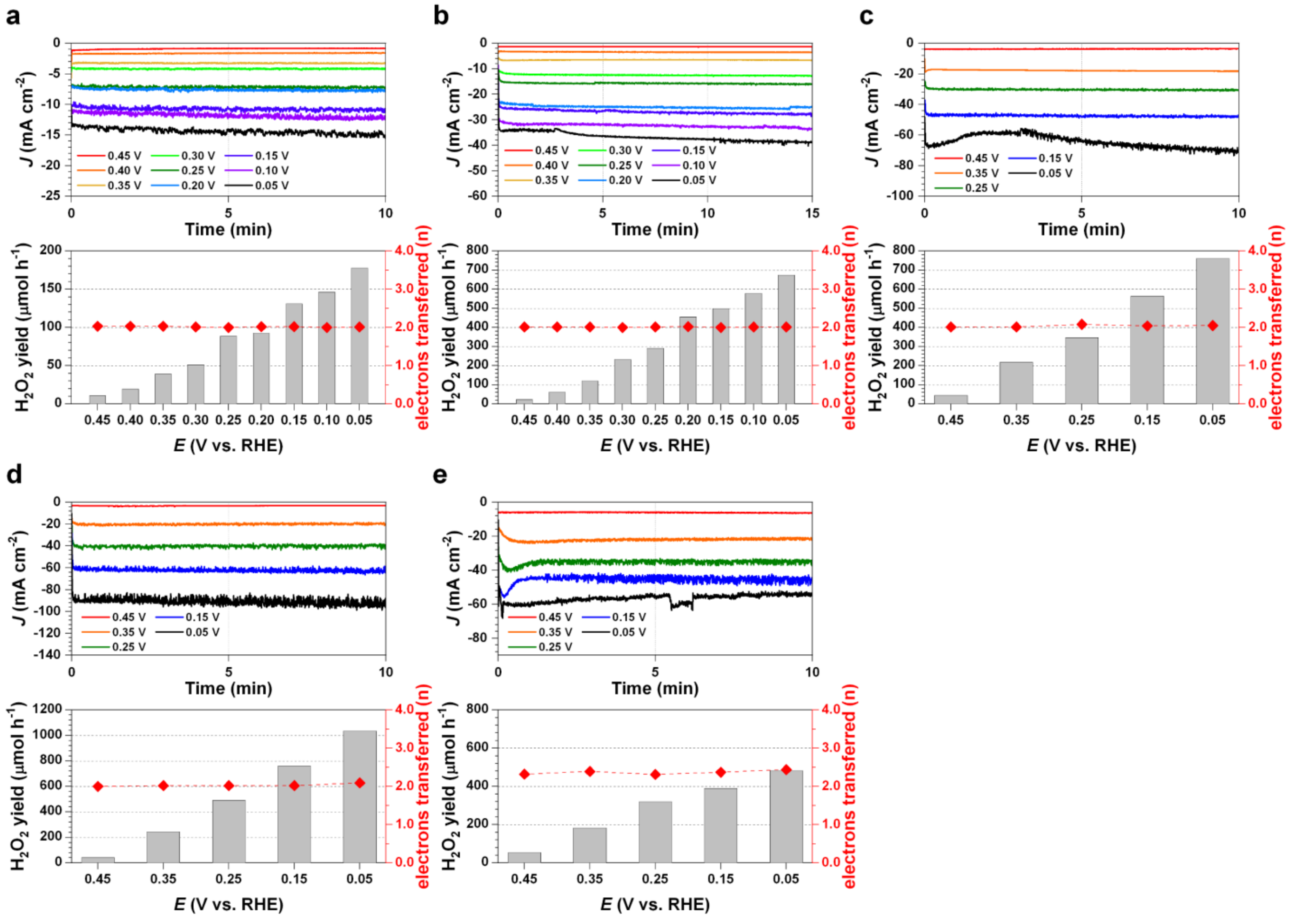

Supplementary Fig. 16 | The ORR CA profiles of D-PSFZ in the $KHCO_3$ conditions. The potential ranges from 0.45 to 0.05 V obtained in O_2- **a** 0.1 M, **b** 0.5 M, **c** 1.0 M, **d** 2.0 M, and **e** saturated $KHCO_3$ on H-cell configuration and their H_2O_2 yield rate and electrons transferred number. The transferred charge and the titration amount that used for to determine H_2O_2 yields are available at **Supplementary Table 5-9.**

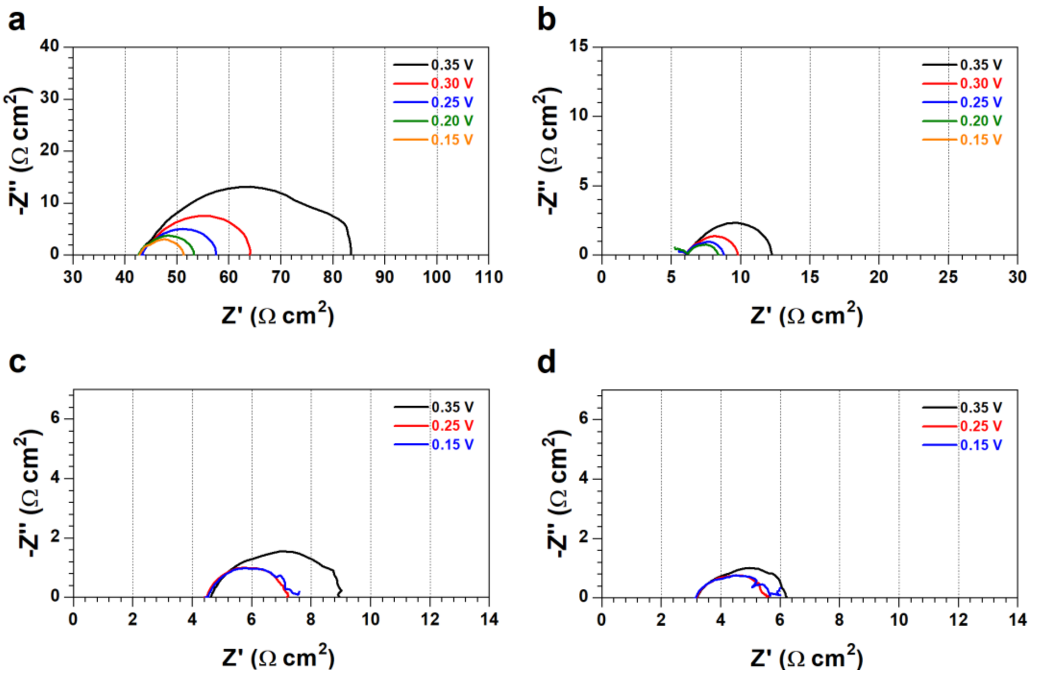

Supplementary Fig. 17 | EIS profiles of D-PSFZ at the potential range from 0.35 to 0.15 V. EIS spectra measured in O_2-saturated **a** 0.1 M, **b** 0.5 M, **c** 1.0 M, and **d** 2.0 M $KHCO_3$ on H-cell configuration. The bubbling of oxygen gas was stopped during EIS measurements.

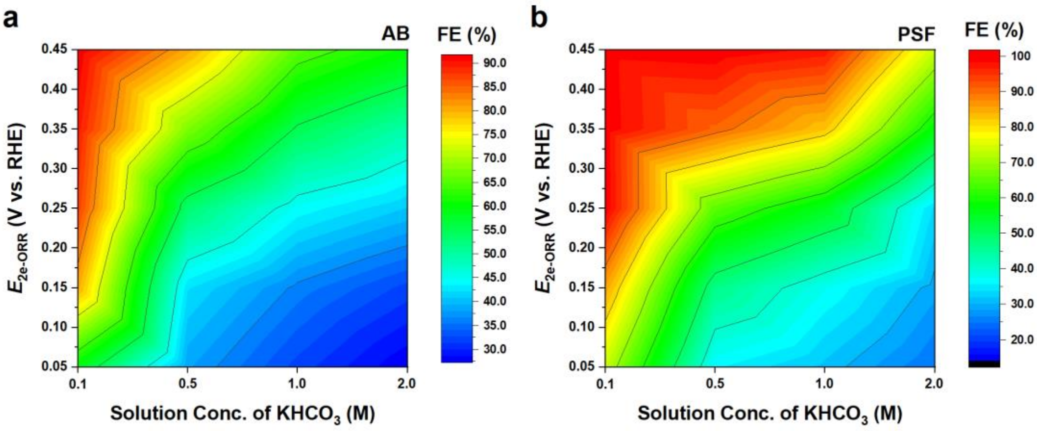

Supplementary Fig. 18 | The colour mapped contour plots for the control groups. The FEs toward 2e-ORR were identified as a function of applied potentials (0.45 to 0.05 V) and solution concentrations (0.1 to 2.0 M $KHCO_3$) for **a** AB carbon black and **b** PSF oxide.

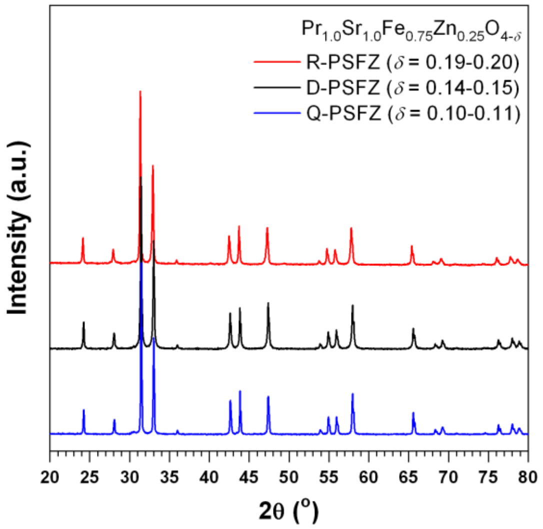

Supplementary Fig. 19 | XRD profiles of δ-controlled D-PSFZ oxides. δ-controlled D-PSFZ oxides are prepared by post-heating procedures and the lower or higher O*v* samples have been prepared by quenching or reducing method (denoted Q-PSFZ and R-PSFZ, respectively) as described in Methods.

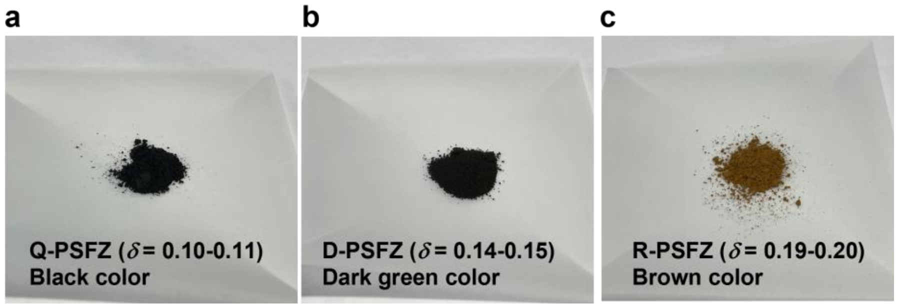

Supplementary Fig. 20 | Digital photographs of δ-controlled D-PSFZ oxides. a Q-PSFZ (δ = 0.10-0.11): black colour; **b** D-PSFZ (δ= 0.14-0.15): dark green colour; **c** R-PSFZ (δ= 0.19-0.20): brown colour. Since the δ values are relevant to the electronic structure of the perovskite oxides, the changes of colour can be found as a function of δ values.

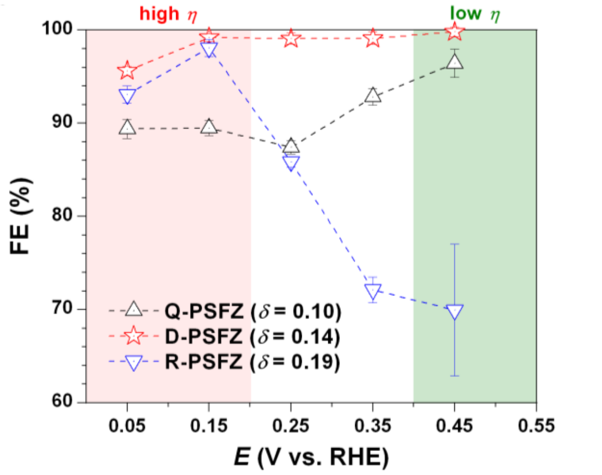

Supplementary Fig. 21 | The FEs toward 2e-ORR for δ-controlled D-PSFZ oxides (*i.e.*, Q-PSFZ ($\delta = 0.10$–0.11); D-PSFZ ($\delta = 0.14$–0.15); R-PSFZ ($\delta = 0.19$–0.20)). The FEs were obtained in the O_2-sat'd 2.0 M $KHCO_3$ condition. Error bars were obtained from at least three times of measurements.

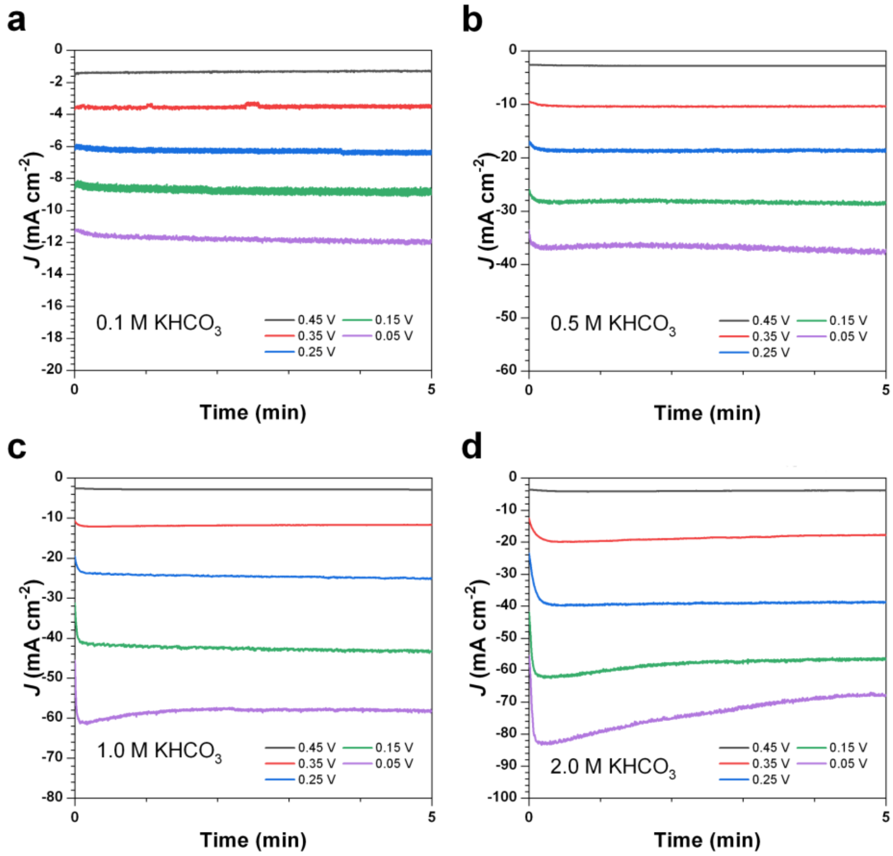

Supplementary Fig. 22 | The ORR CA profiles of R-PSFZ in the $KHCO_3$ conditions. The potential ranges from 0.05 to 0.45 V toward 2e-ORR in **a** 0.1 M, **b** 0.5 M, **c** 1.0 M, and **d** 2.0 $KHCO_3$ on H-cell configuration. The FEs, transferred charge and the titration amount that used for to determine H_2O_2 yields are available at **Supplementary Table 10-13.**

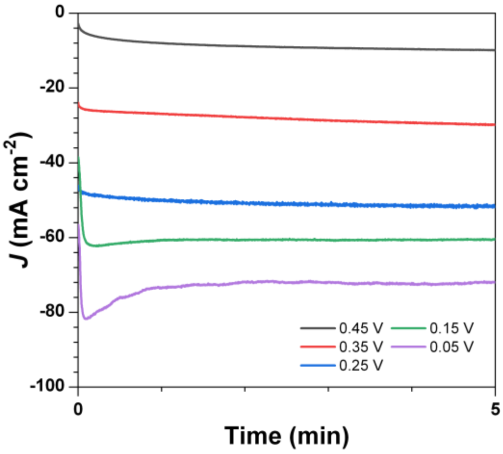

Supplementary Fig. 23 | The ORR CA profiles of Q-PSFZ. The potential ranges from 0.05 to 0.45 V toward 2e-ORR in 2.0 M $KHCO_3$ on H-cell configuration. The FEs, transferred charge and the titration amount that used for to determine H_2O_2 yields are available at **Supplementary Table 14.**

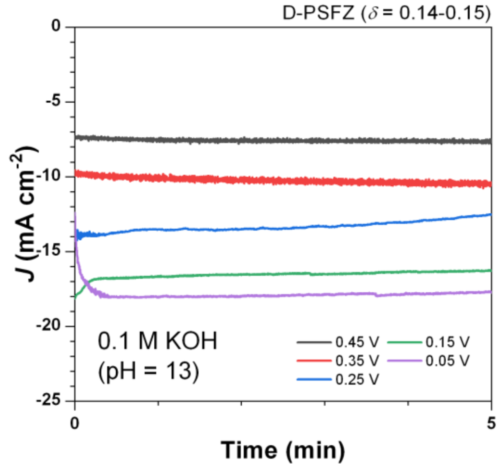

Supplementary Fig. 24 | The ORR CA profiles of D-PSFZ. The potential ranges from 0.05 to 0.45 V toward 2e-ORR in 0.1 M KOH (pH = 13.0) on H-cell configuration. The FEs, transferred charge and the titration amount that used for to determine H_2O_2 yields are available at **Supplementary Table 15.**

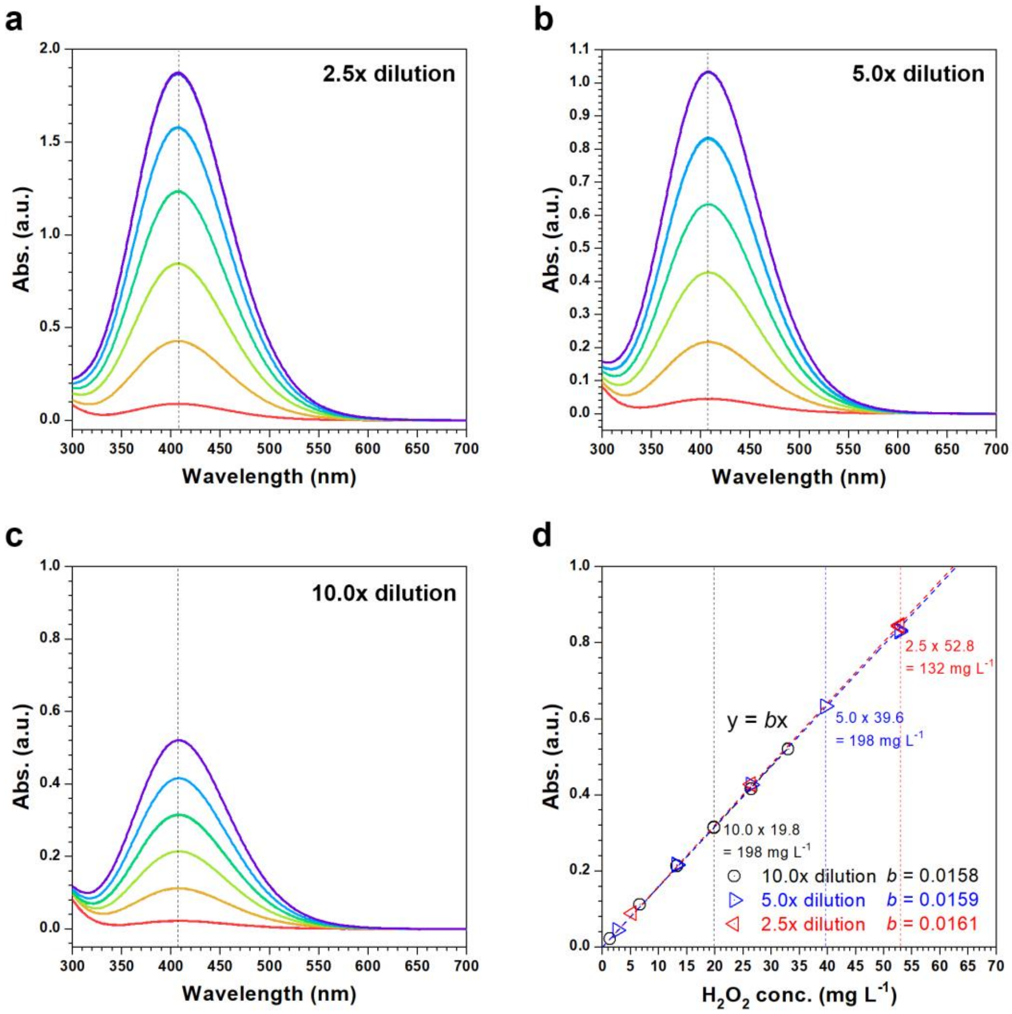

Supplementary Fig. 25 | UV-vis calibration spectra. 20 mM $TiOSO_4$ dissolved in 1.0 M H_2SO_4 solution was used as a coloring-reagent. Standard H_2O_2 solutions with different concentrations were prepared by diluting a 3 wt.% H_2O_2 solution (Sigma-aldrich) in 0.5 M $KHCO_3$. Standard solutions are as follows: Red: 13.2 mg L^{-1}. Yellow: 66 mg L^{-1}. Light green: 132 mg L^{-1}. Green: 198 mg L^{-1}. Blue: 264 mg L^{-1}. Violet: 330 mg L^{-1}. Each standard solutions were further diluted with addition of the $TiOSO_4$ solution. **a** 2.5x dilution (*i.e.*, 0.4 mL of H_2SO_4 + 0.4 mL of standard H_2O_2 solution + 0.2 mL of $TiOSO_4$ solution), **b** 5.0x dilution (*i.e.*, 0.6 mL of H_2SO_4 + 0.2 mL of standard H_2O_2 solution + 0.2 mL of $TiOSO_4$ solution), **c** 10.0x dilution (*i.e.*, 0.7 mL of H_2SO_4 + 0.1 mL of standard H_2O_2 solution + 0.2 mL of $TiOSO_4$ solution). **d** Calibration fitting profiles obtained from **Supplementary Fig. 24a-24c**. The intercept value was fixed at 0.

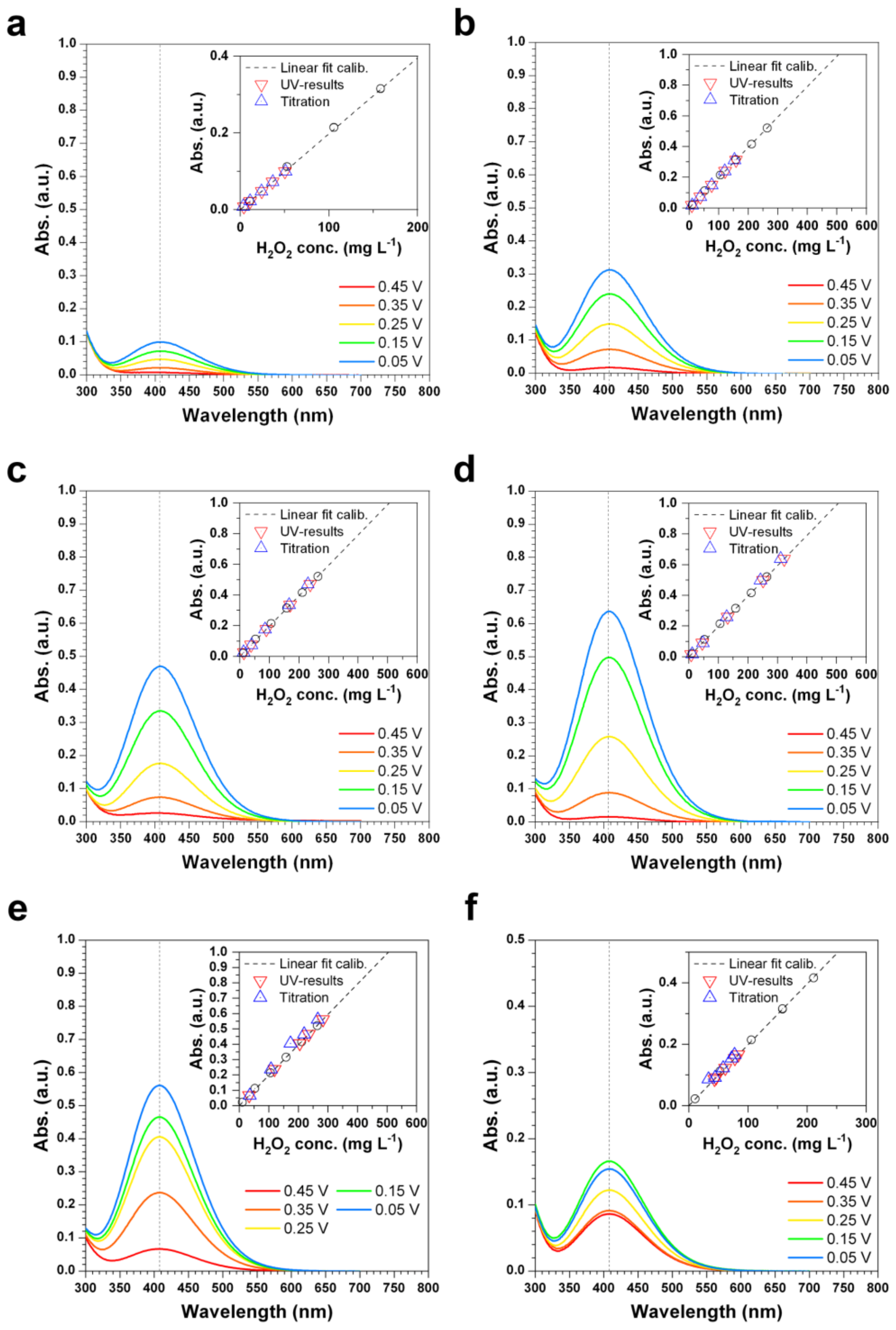

Supplementary Fig. 26 | UV-vis spectra of H_2O_2 yields for R-PSFZ. H_2O_2 products were accumulated in **a** 0.1 M, **b** 0.5 M, **c** 1.0 M, **d** 2.0 M $KHCO_3$ condition. **e** UV-vis spectra of H_2O_2 yields for Q-PSFZ accumulated in 1.0 M $KHCO_3$ related with **Supplementary Fig. 22. f** UV-vis spectra of H_2O_2 yields for D-PSFZ accumulated in 0.1 M KOH (pH = 13.0) related with Supplementary Fig. 23. Each H_2O_2 yields measured from the UV-vis are shown as insets. For better comparison, the absorbance values of the titration method (the inverted triangle points, blue) were supposed to be same to that of the UV-vis method. The well-overlapping of the triangle (red) and the inverted triangle (blue) indicates the obtained H_2O_2 yields are similar.

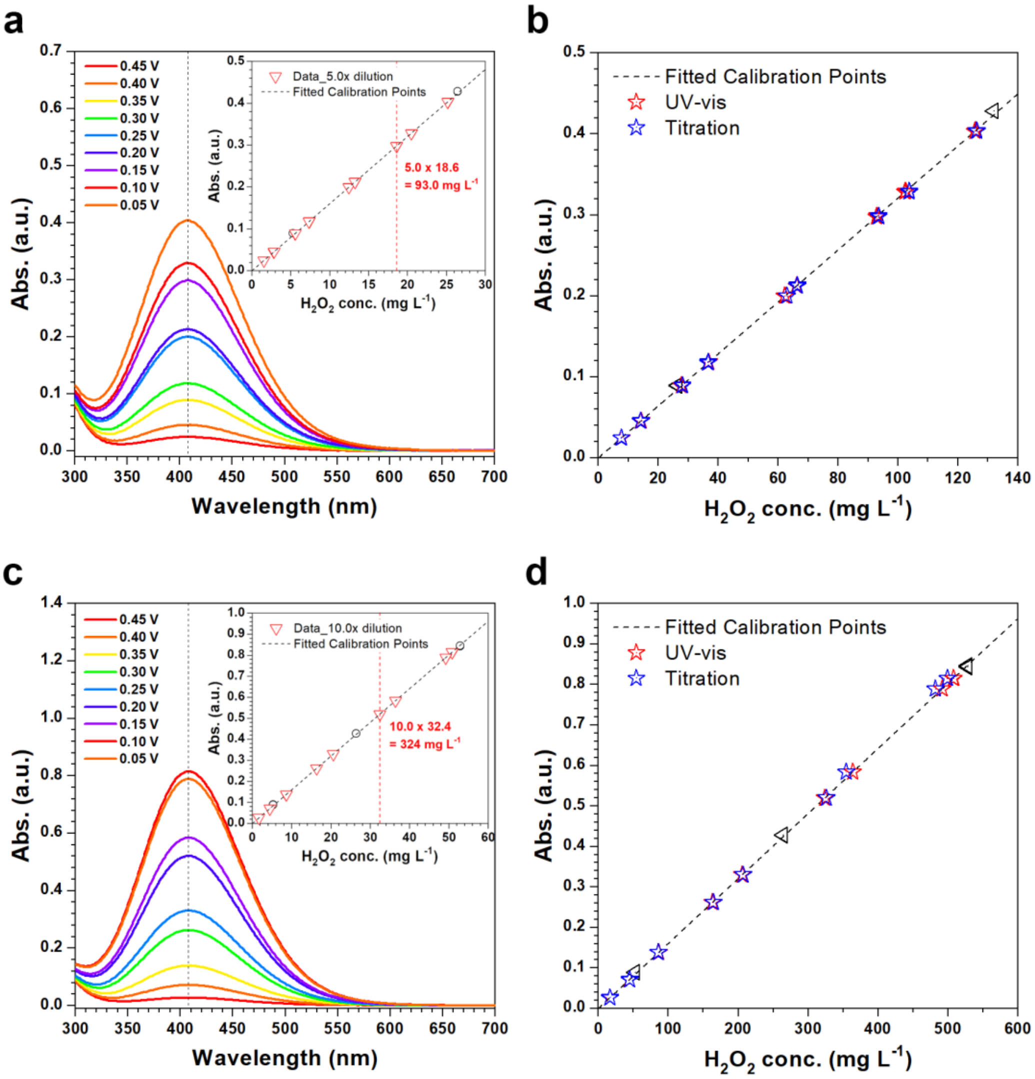

Supplementary Fig. 27 | UV-vis spectra of H_2O_2 yields. H_2O_2 products were accumulated in **a** the 0.1 M and **b** the 0.5 M $KHCO_3$ condition toward ORR and the calculation of each H_2O_2 yields that measured from the UV-vis as an inset. The comparison the H_2O_2 yields obtained from the UV-vis and the titration measured in **c** the 0.1 M and **d** 0.5 M $KHCO_3$ condition. For better comparison, the absorbance values of the titration method (the inverted triangle points, blue) were supposed to be same to that of the UV-vis method. The well-overlapping of the triangle (red) and the inverted triangle (blue) indicates the obtained H_2O_2 yields are similar.

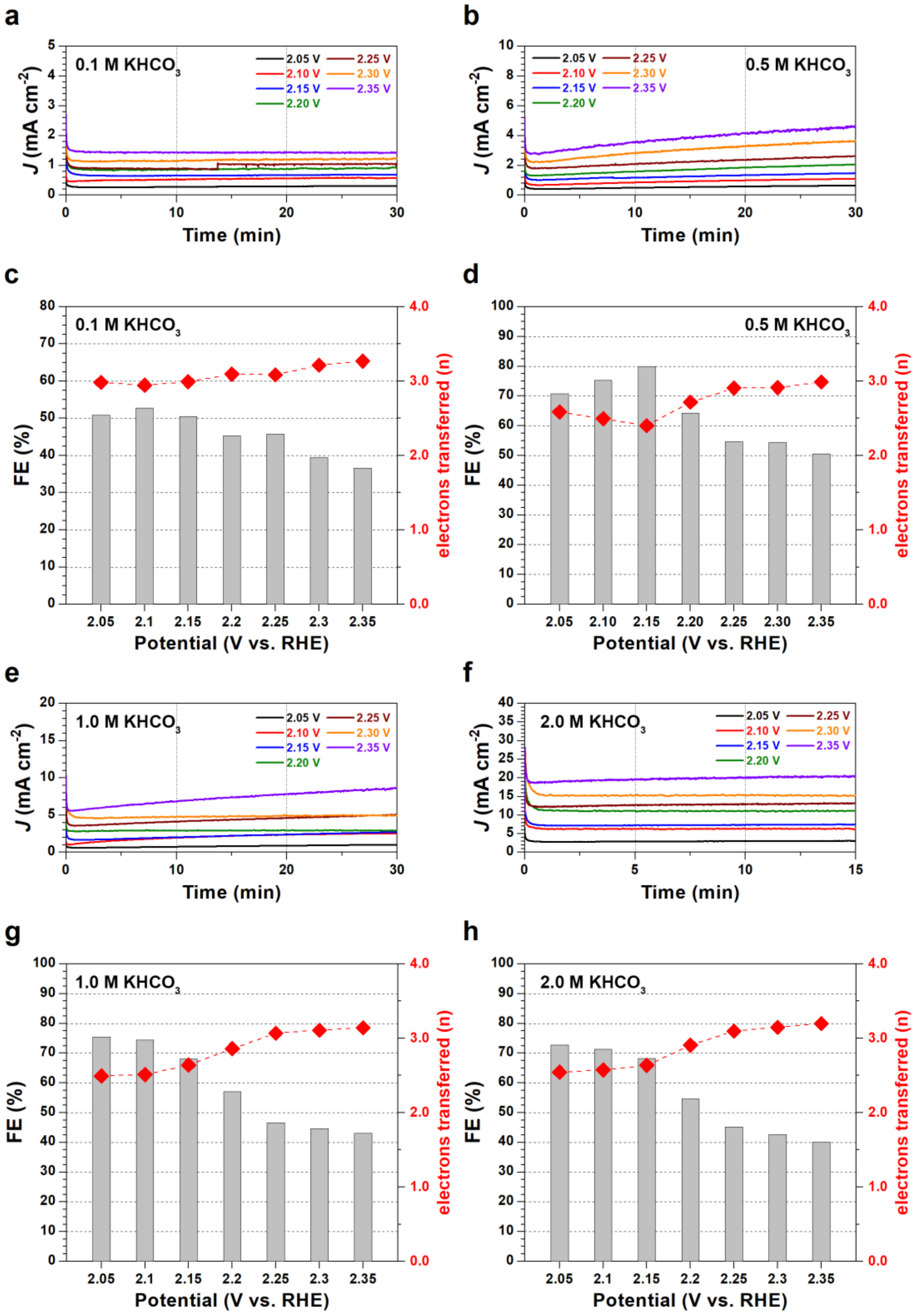

Supplementary Fig. 28 | The WOR CA profiles of D-PSFZ in the $KHCO_3$ conditions. The potential ranges from 2.05 to 2.35 V toward 2e-WOR and the corresponding FE and n value measured in **a** and **c** 0.1 M, **b** and **d** 0.5 M, **e** and **g** 1.0 M, and **f** and **h** 2.0 $KHCO_3$ on H-cell configuration. The transferred charge and the titration amount that used for to determine H_2O_2 yields are available at **Supplementary Table 16-19.**

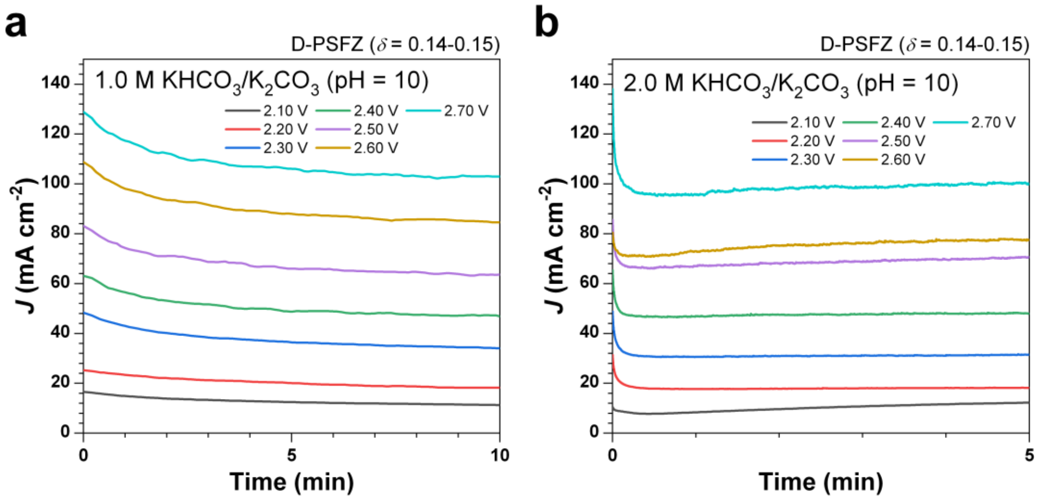

Supplementary Fig. 29 | The WOR CA profiles of D-PSFZ in the $KHCO_3/K_2CO_3$ conditions. The potential ranges from 2.20 to 2.70 V toward 2e-WOR measured in **a** 1.0 M, and **b** 2.0 M $KHCO_3/K_2CO_3$ (pH = 10) on H-cell configuration. The FEs, transferred charge and the titration amount that used for to determine H_2O_2 yields are available at **Supplementary Table 20-21.**

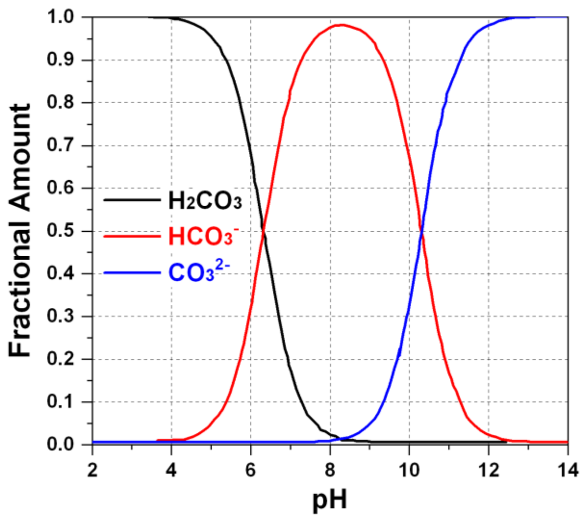

Supplementary Fig. 30 | Fractional amount of carbonic acid, bicarbonate, and carbonate ions in various pH conditions. To utilize carbonate ions for 2e-WOR, the pH level of solution should be alkaline conditions.

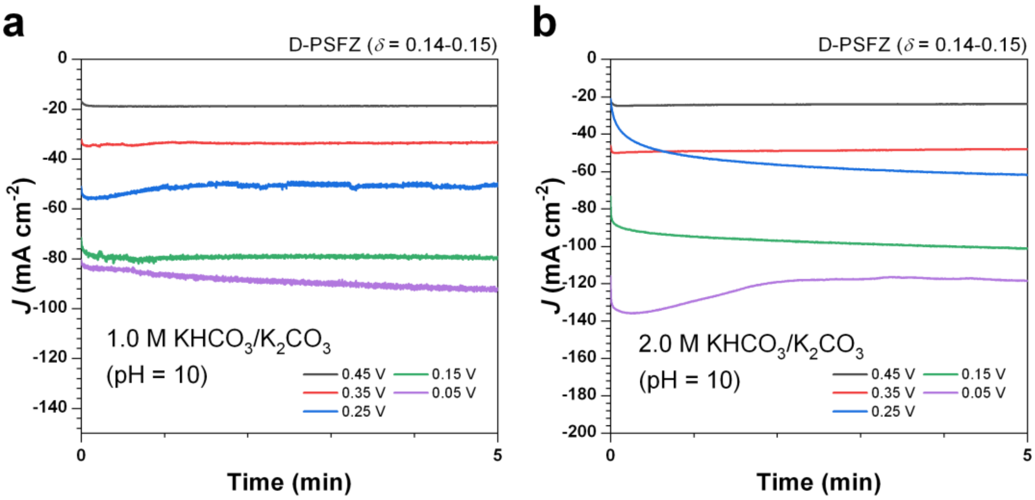

Supplementary Fig. 31 | The ORR CA profiles of D-PSFZ in the $KHCO_3/K_2CO_3$ conditions. The potential ranges from 0.05 to 0.45 V toward 2e-ORR measured in **a** 1.0 M, and **b** 2.0 M $KHCO_3/K_2CO_3$ (pH = 10) on H-cell configuration. The transferred charge and the titration amount that used for to determine H_2O_2 yields are available at **Supplementary Table 22-23.**

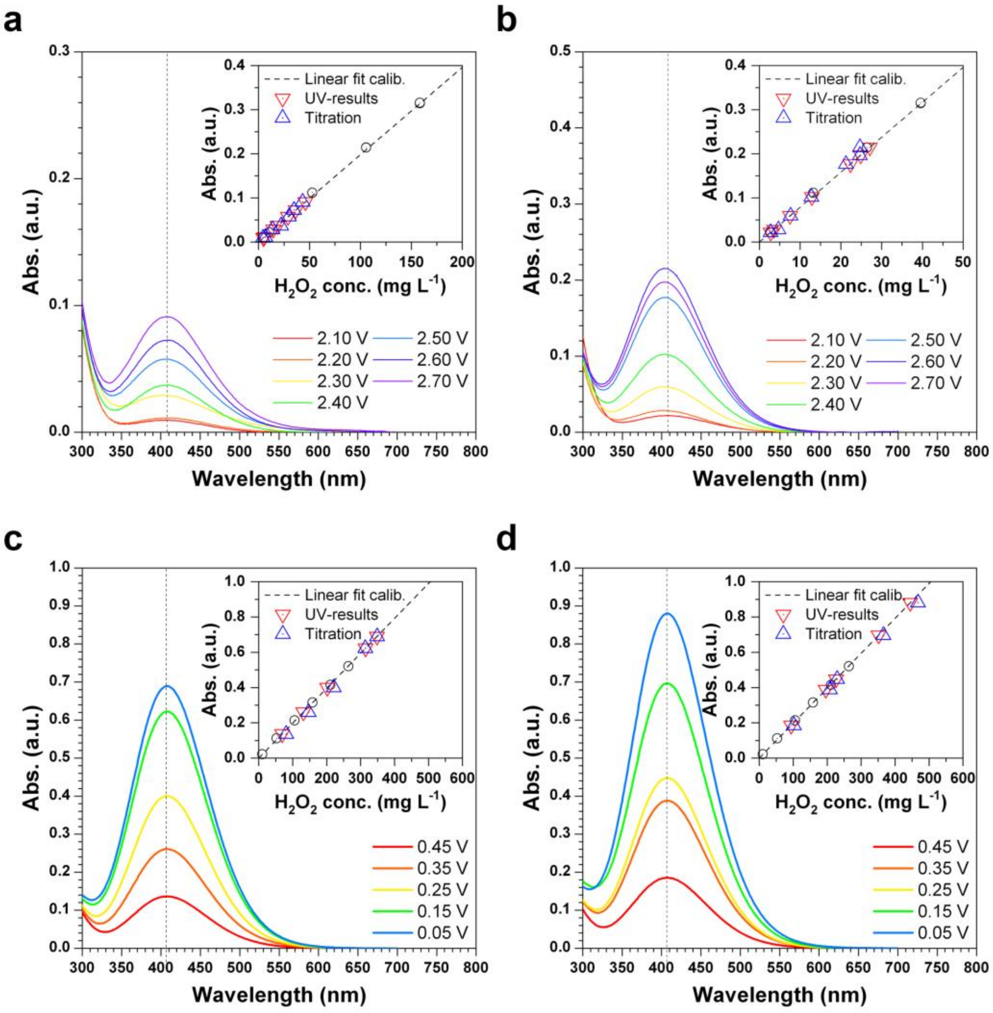

Supplementary Fig. 32 | UV-vis spectra of H_2O_2 yields accumulated during 2e-WOR. **a** 1.0 M, **b** and 2.0 M $KHCO_3$/ K_2CO_3 conditions, and obtained during 2e-ORR in O_2-sat'd **c** 1.0 M, **d** and 2.0 M $KHCO_3$/ K_2CO_3 conditions. Each H_2O_2 yields measured from the UV-vis are shown as insets. For better comparison, the absorbance values of the titration method (the inverted triangle points, blue) were supposed to be same to that of the UV-vis method. The well-overlapping of the triangle (red) and the inverted triangle (blue) indicates the obtained H_2O_2 yields are similar.

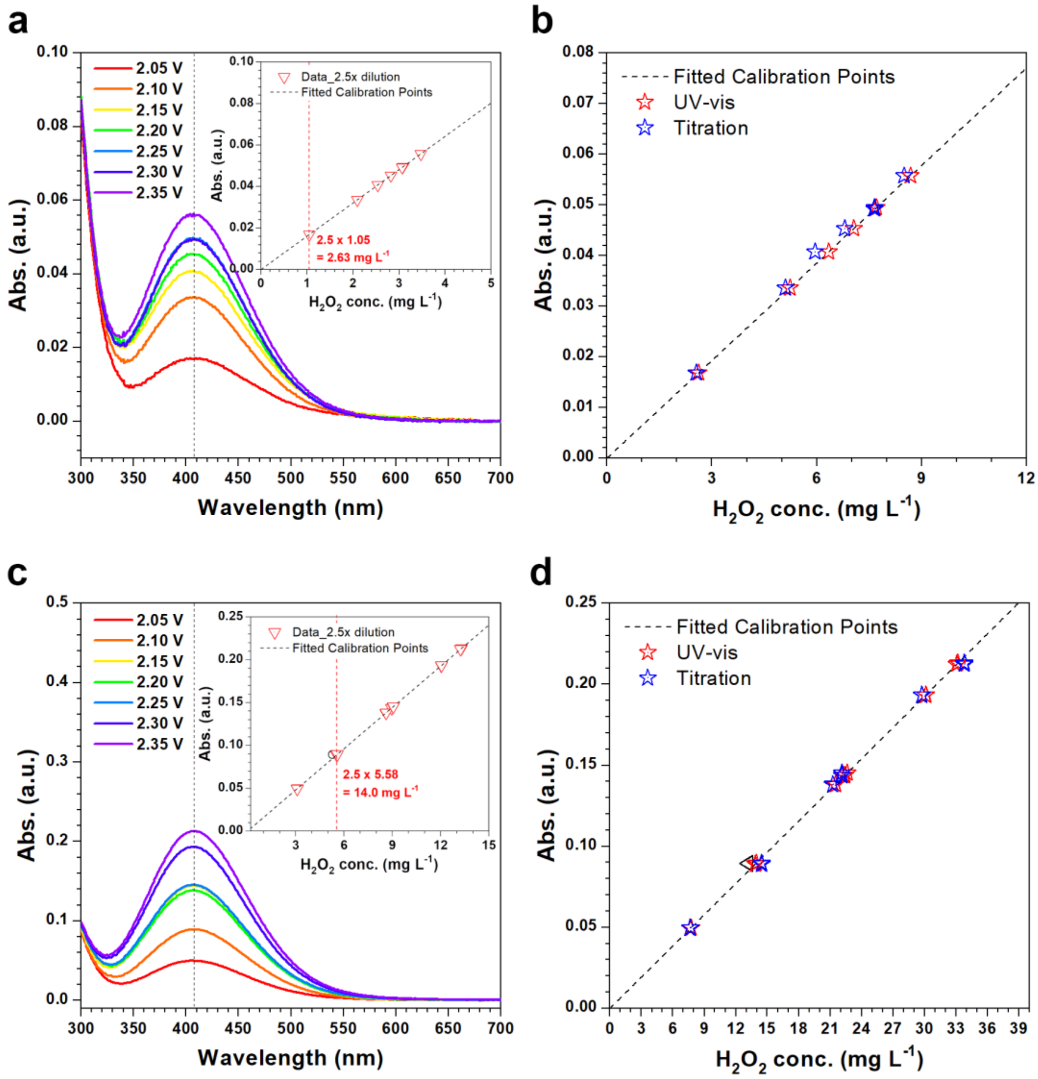

Supplementary Fig. 33 | UV-vis spectra of H_2O_2 yields in the $KHCO_3$ conditions. H_2O_2 products were accumulated in **a** 0.1 M and **b** 0.5 M $KHCO_3$ conditions toward WOR and the calculation of each H_2O_2 yields that measured from the UV-vis as an inset. The comparison the H_2O_2 yields obtained from the UV-vis and the titration measured in **c** the 0.1 M and **d** 0.5 M $KHCO_3$ condition. For better comparison, the absorbance values of the titration method (the inverted triangle points, blue) were supposed to be same to that of the UV-vis method. The well-overlapping of the triangle (red) and the inverted triangle (blue) indicates the obtained H_2O_2 yields are similar.

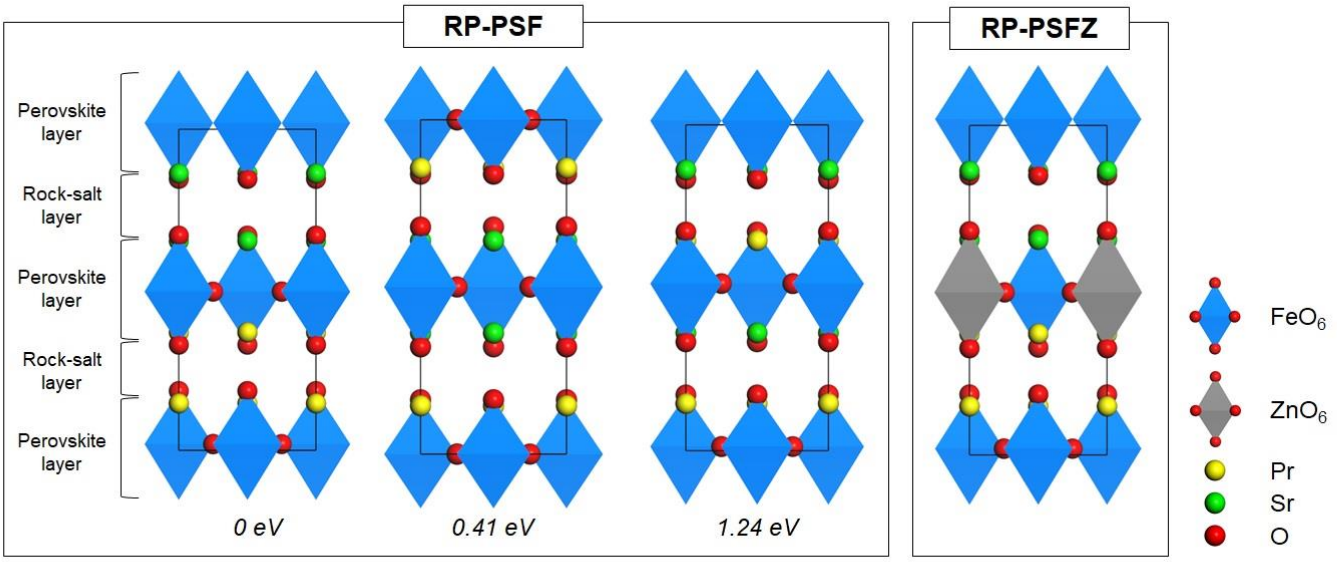

Supplementary Fig. 34 | The optimized configurations of RP-PSF and RP-PSFZ. Three different distributions of A-site ion distribution in RP-PSF were compared in terms of relative energy. Note that the relative energies were labelled below each system. Zn ion was substituted in the most stable RP-PSF framework. Blue and gray octahedra represent Fe and Zn site, while yellow, green, and red balls represent Pr, Sr, and O atoms, respectively.

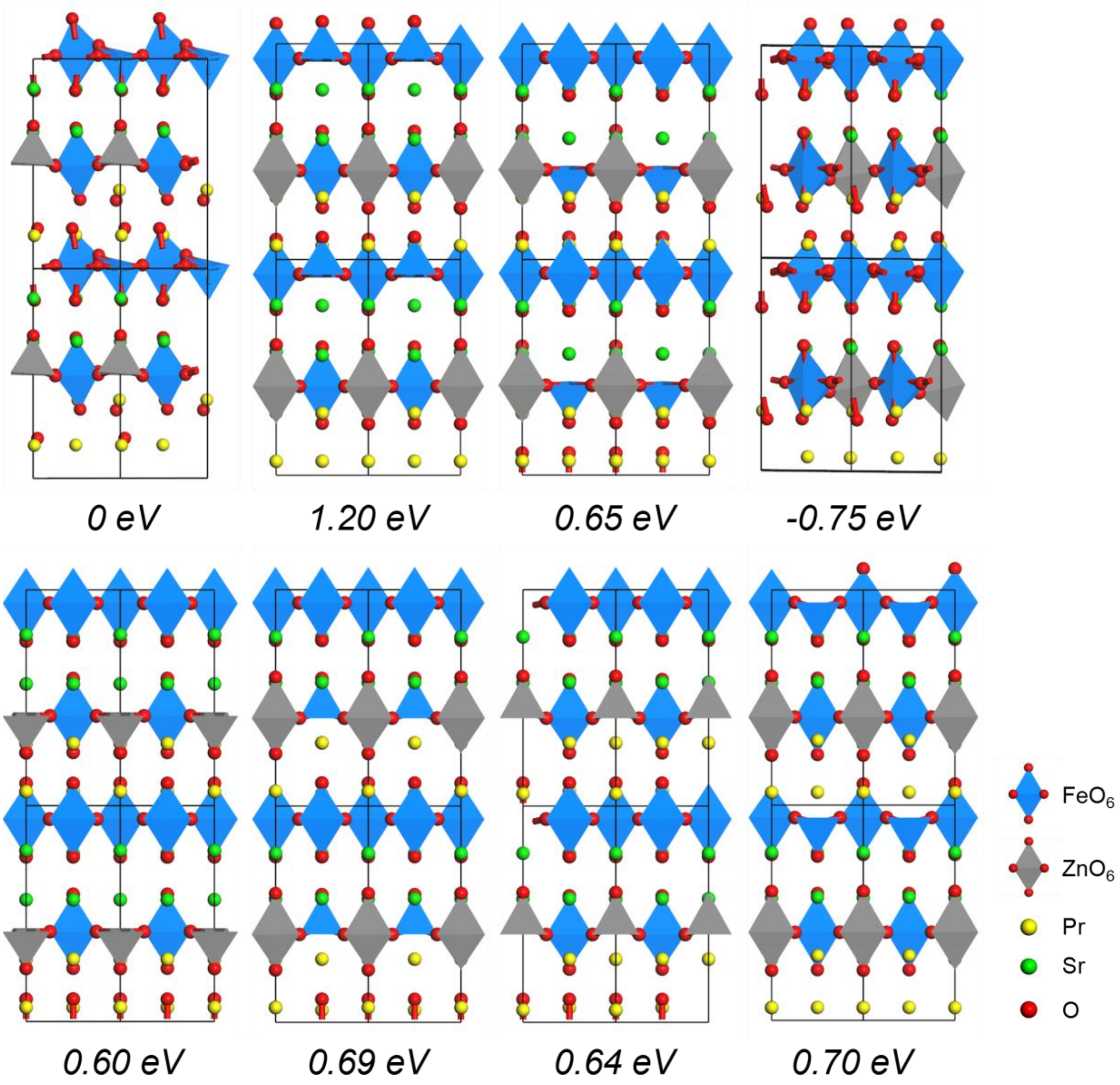

Supplementary Fig. 35 | The optimized configurations of defective RP-PSFZ. The relative energies of eight different configurations were compared each other while the relative energies were labelled below each configuration. Blue and gray octahedra represent Fe and Zn site, while yellow, green, and red balls represent Pr, Sr, and O atoms, respectively.

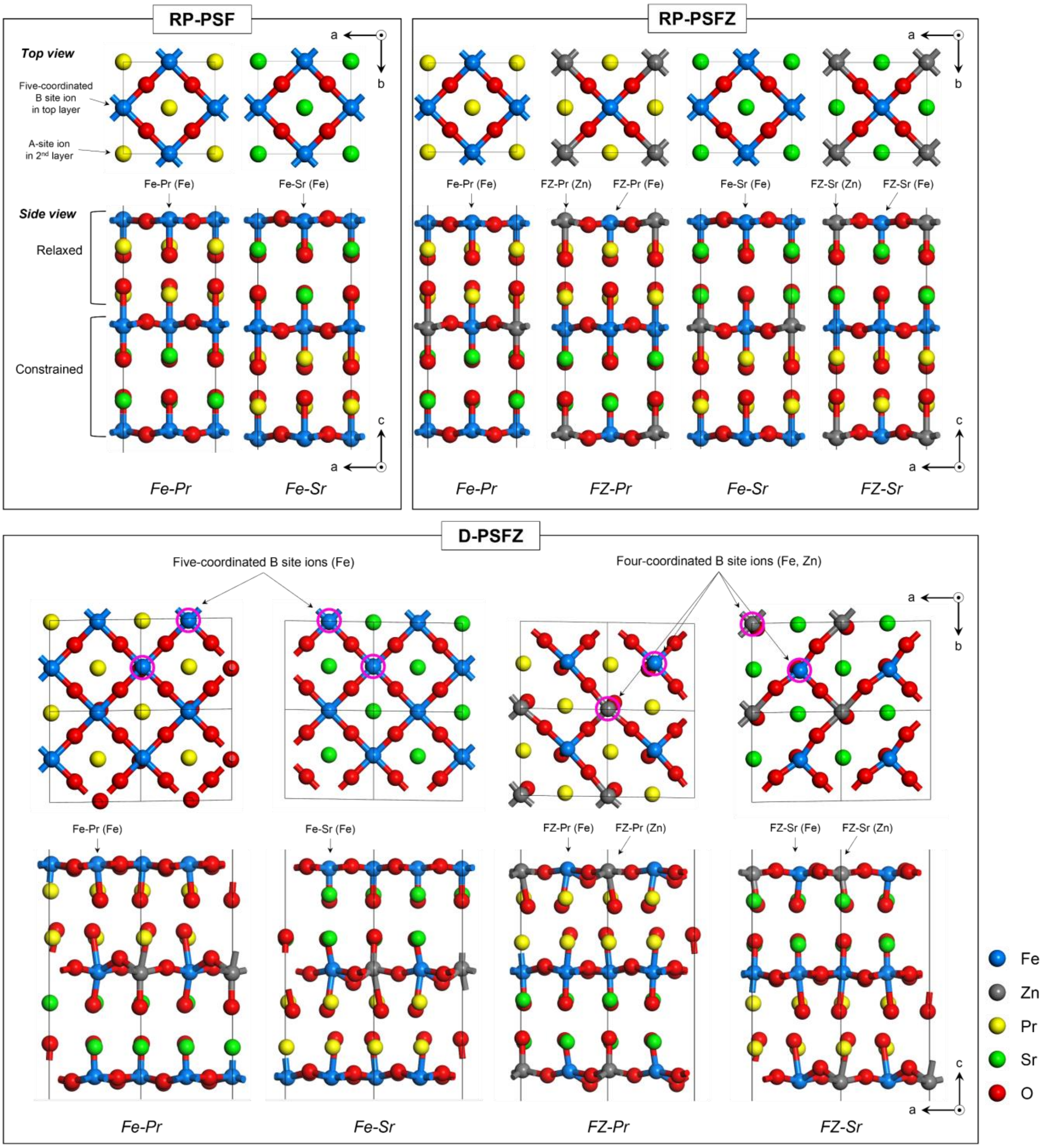

Supplementary Fig. 36 | The considered surface structures of RP-PSF, RP-PSFZ, and D-PSFZ. Half of bottom layers were constrained during the simulation. Blue, gray, yellow, green, and red balls represent Fe, Zn Pr, Sr, and O atoms, respectively. Each model was named by the information of B-site ions in the top layer and A-site ions in the second layer; Fe-Pr, Fe-Sr, FZ-Pr, FZ-Sr ('FZ' represents Fe/Zn layer). The considered active sites were labelled between top and side views of each model system.

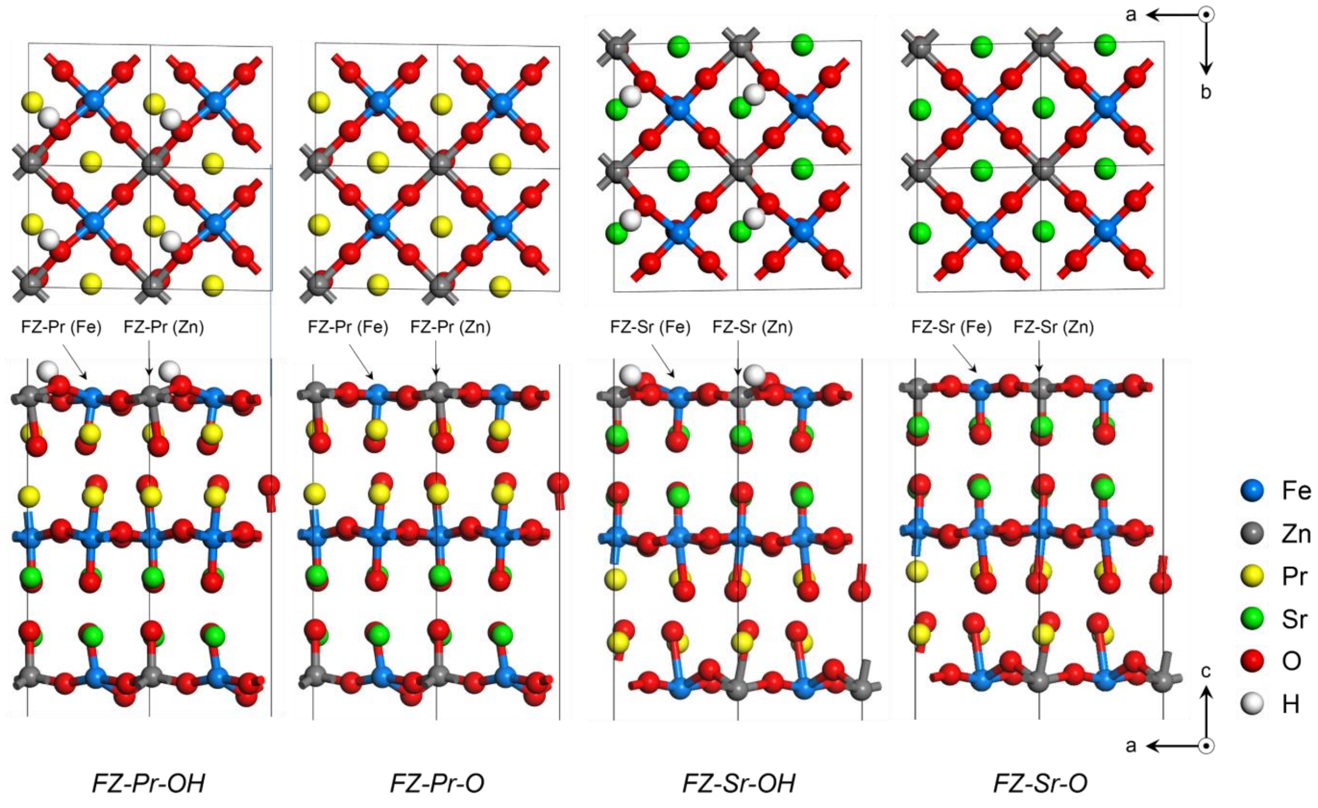

Supplementary Fig. 37 | HO* and O* covered D-PSFZ structures. Half of bottom layers were constrained during the simulation. Blue, gray, yellow, green, red, and white balls represent Fe, Zn Pr, Sr, O, and H atoms, respectively. When defective Fe/Zn perovskite surface was healed by HO* (O*), the model was called as FZ-Pr/Sr-OH (FZ-Pr/Sr-O).

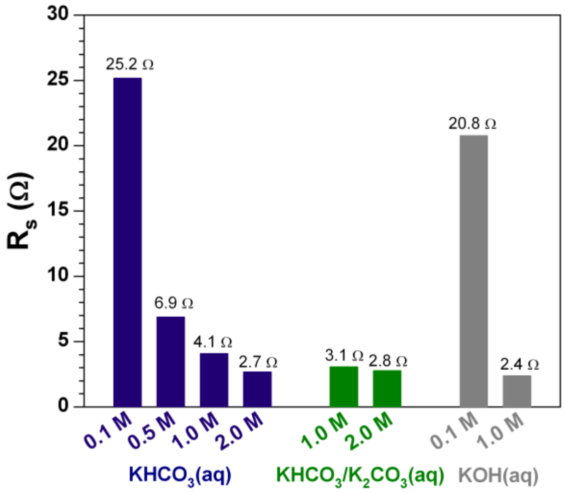

Supplementary Fig. 38 | Solution resistance values obtained in various electrolyte conditions. The D-PSFZ coated electrodes were used for the measurements in a three-electrode configuration.

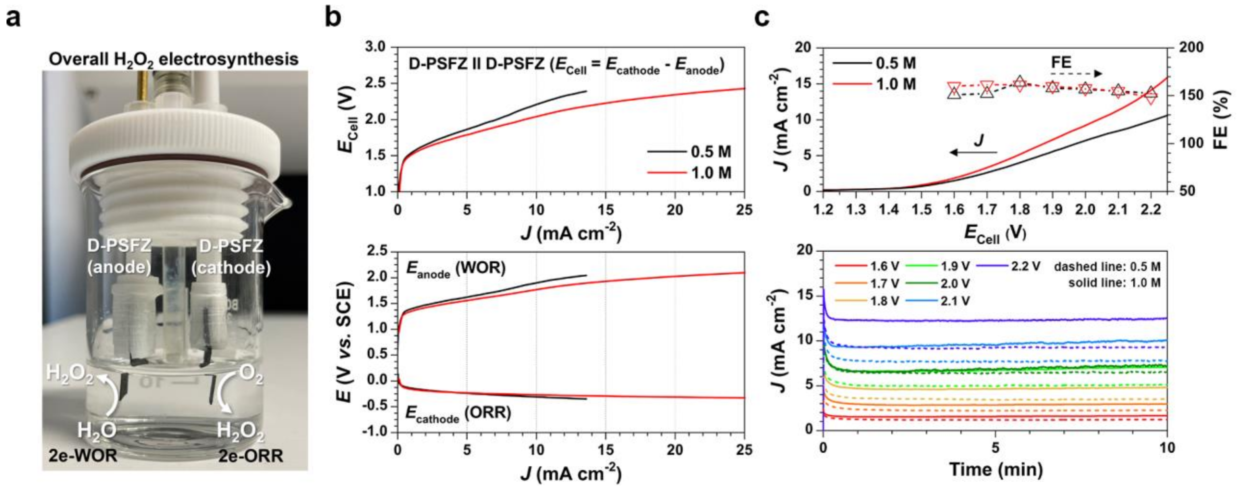

Supplementary Fig. 39 | Overall H_2O_2 electrolysis performance in the $KHCO_3$ condition. a Photograph of the membrane-free overall H_2O_2 electrosynthesis unit in a three-electrode configuration using the D-PSFZ as both cathode and anode (denoted as D-PSFZ || D-PSFZ) with SCE reference electrode. **b** Polarization I-V profiles of D-PSFZ || D-PSFZ measured in O_2-sat'd 0.5 M or 1.0 M $KHCO_3$. **c** Faradaic efficiency profiles obtained by the titration method with the I-V polarization curves (the upper part). The CA profiles measured in the cell voltage between 1.6 to 2.2 V (the lower part). The dashed and the solid lines indicate the electrolyte conditions are 0.5 M and 1.0 M KHCO3, respectively. The transferred charge and the titration amount that used for to determine H_2O_2 yields are available at **Supplementary Table 24-25**.

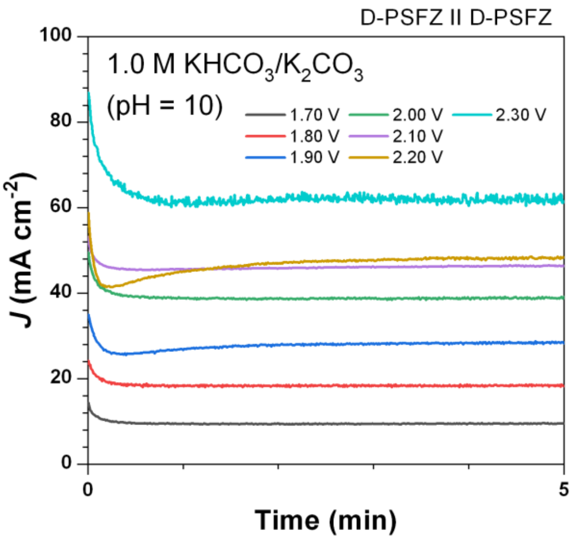

Supplementary Fig. 40 | The CA profiles of D-PSFZ || D-PSFZ full-cell in the $KHCO_3/K_2CO_3$ condition. The cell potential ranges from 1.70 to 2.30 V measured in 1.0 M $KHCO_3/K_2CO_3$ (pH = 10). The transferred charge and the titration amount that used for to determine H_2O_2 yields are available at **Supplementary Table 26.**

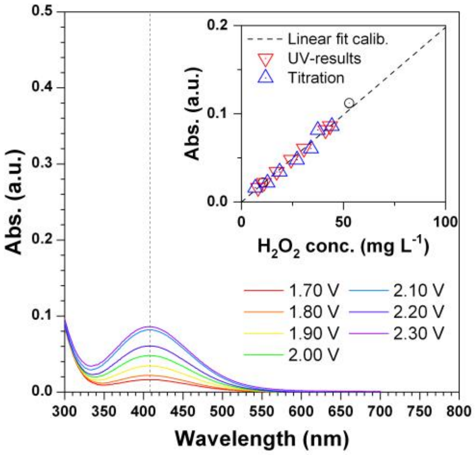

Supplementary Fig. 41 | UV-vis spectra of H_2O_2 yields for D-PSFZ || D-PSFZ full-cell testing accumulated in the 1.0 M $KHCO_3$/K_2CO_3 condition. Each H_2O_2 yields measured from the UV-vis are shown as insets. For better comparison, the absorbance values of the titration method (the inverted triangle points, blue) were supposed to be same to that of the UV-vis method. The well-overlapping of the triangle (red) and the inverted triangle (blue) indicates the obtained H_2O_2 yields are similar.

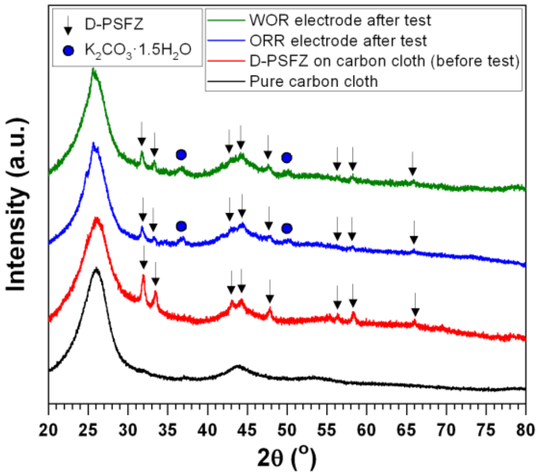

Supplementary Fig. 42 | XRD profiles of D-PSFZ electrodes used for the stability testing of the D-PSFZ || D-PSFZ full-cell in the 2.0 M $KHCO_3/K_2CO_3$ condition. The residue of potassium carbonate hydrate solid can be found after drying of the electrolyte.

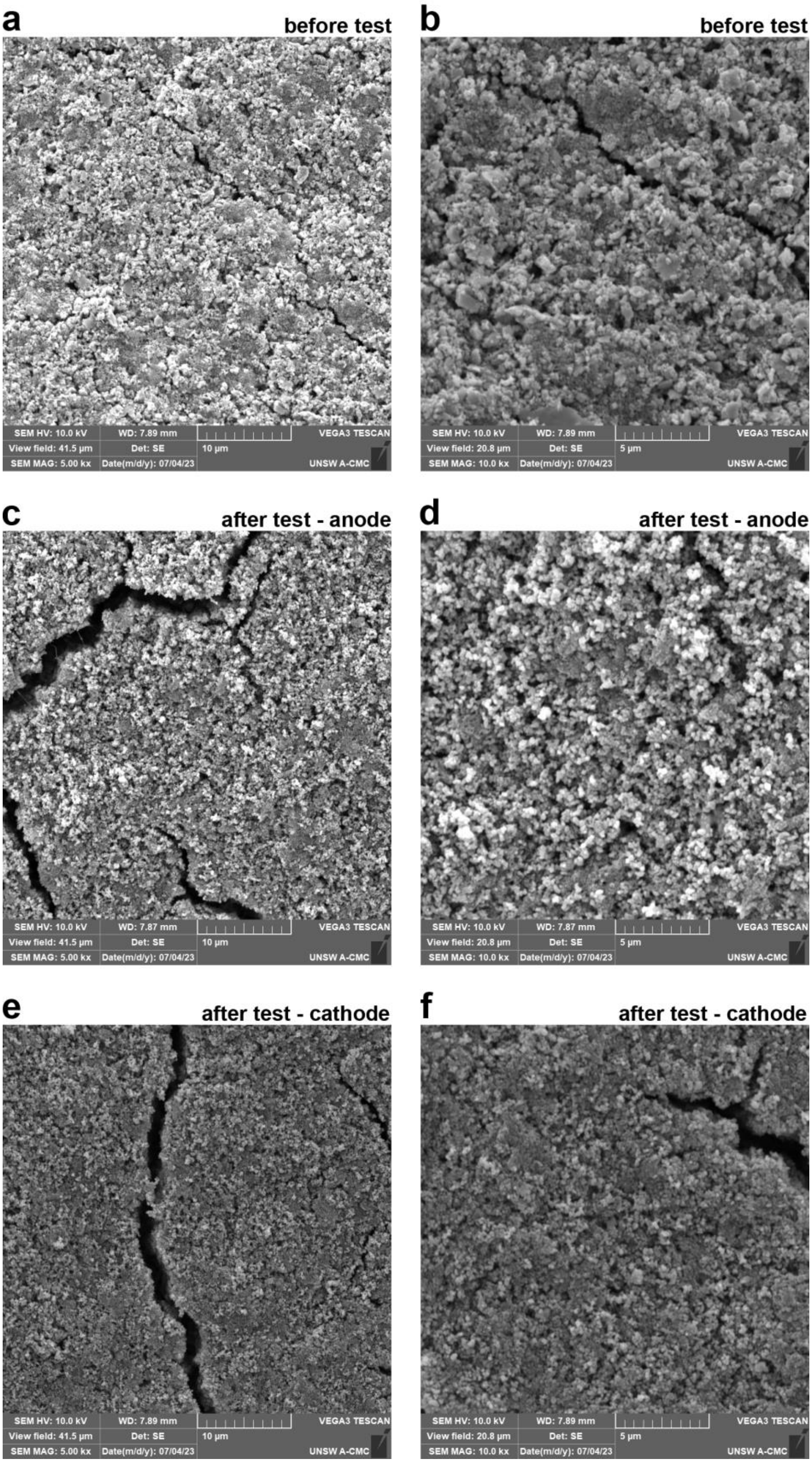

Supplementary Fig. 43 | SEM images of D-PSFZ electrodes used for the stability testing of the D-PSFZ || D-PSFZ full-cell in the 2.0 M $KHCO_3$/K_2CO_3 condition. **a** and **b** as-prepared electrode, **c** and **d** the anode after testing, **e** and **f** the cathode after testing.

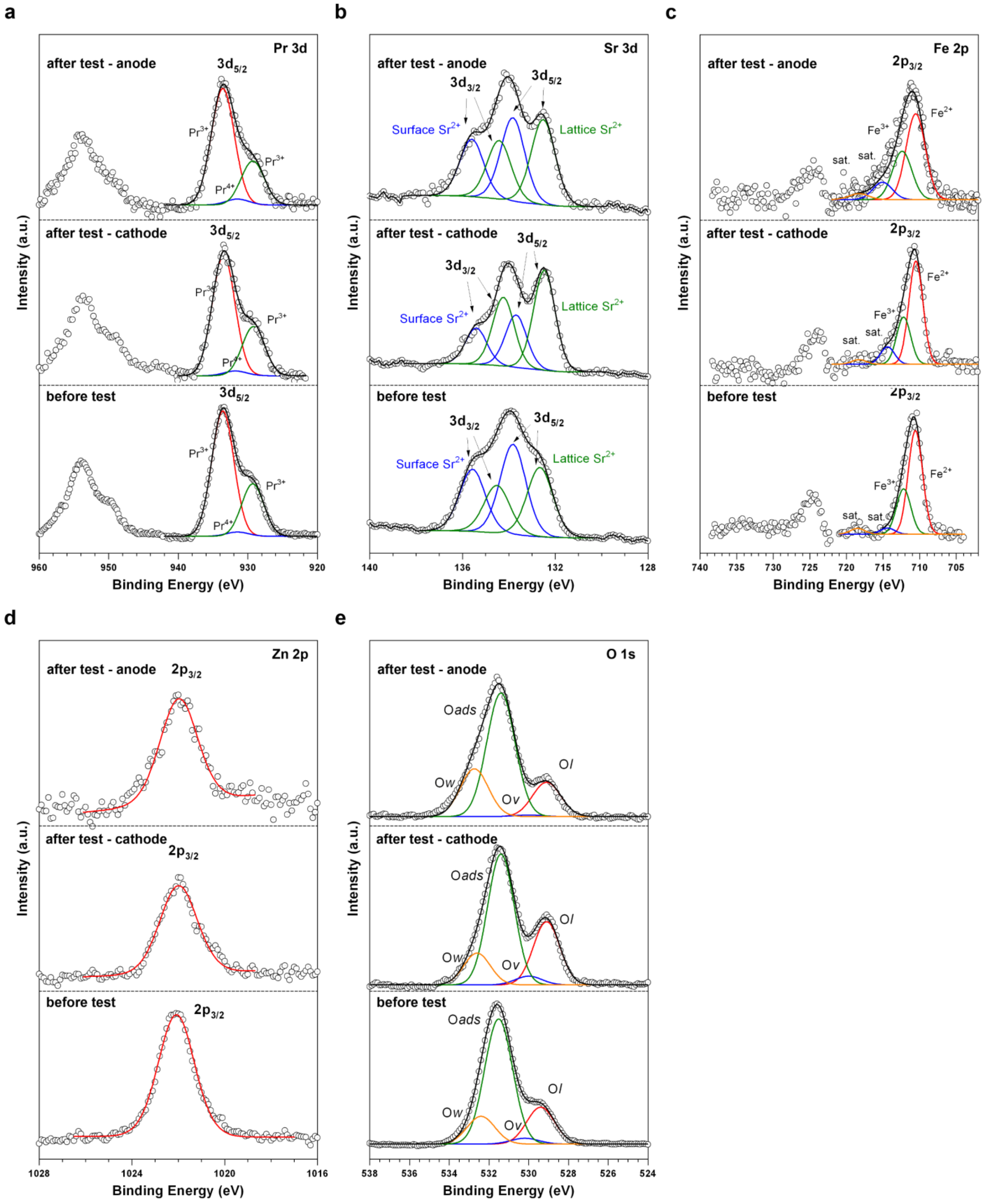

Supplementary Fig. 44 | High-resolution surface XPS scan of D-PSFZ electrodes used for the stability testing of the D-PSFZ || D-PSFZ full-cell in the 2.0 M $KHCO_3/K_2CO_3$ condition. a, Pr 3*d* **b**, Sr 3*d* **c**, Fe 2*p* **d**, Zn 2*p* **e**, O 1*s* of D-PSFZ electrodes.

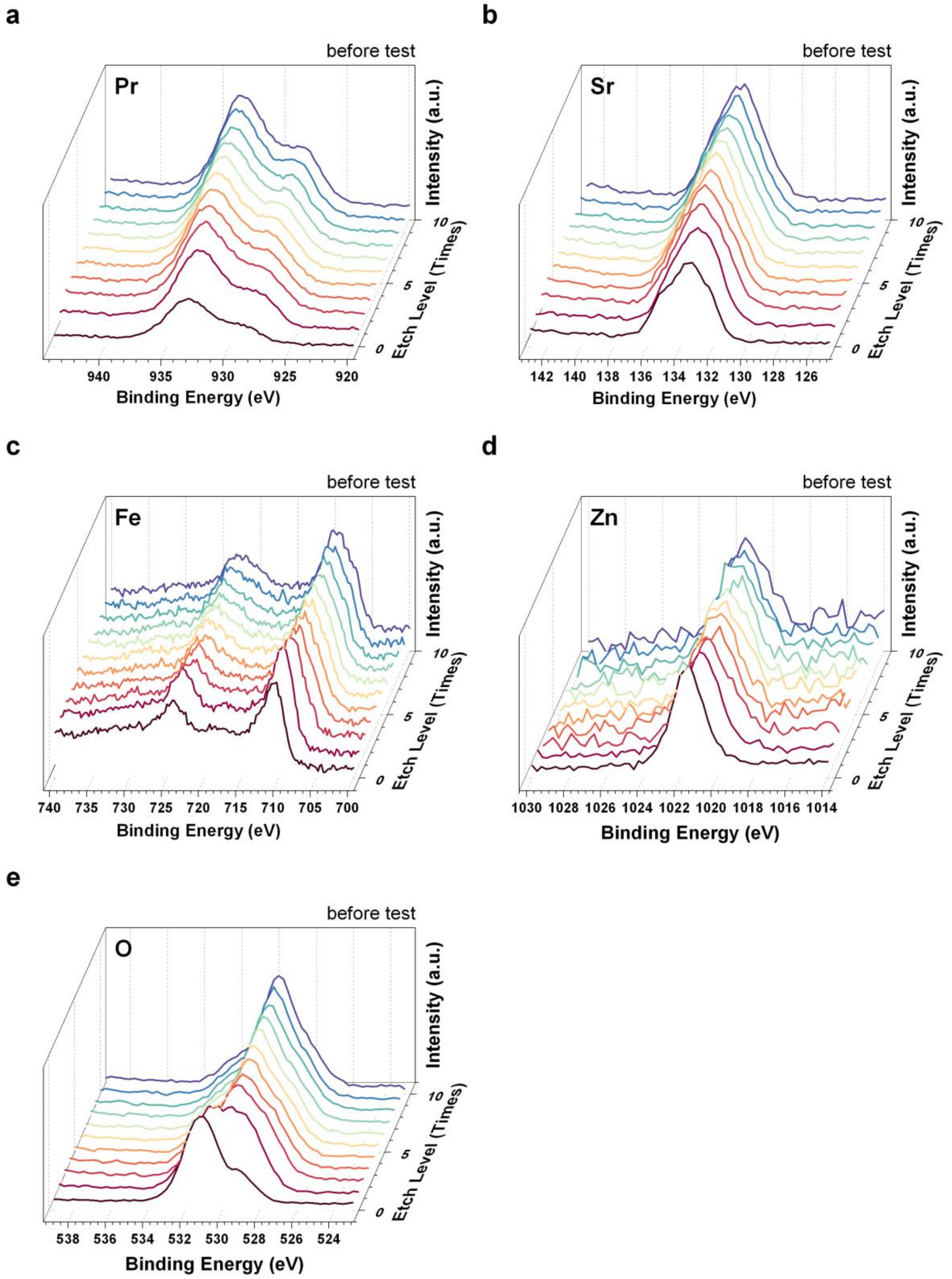

Supplementary Fig. 45 | XPS depth profiles of the as-prepared D-PSFZ electrode. XPS spectra for **a** Pr 3*d*, **b** Sr 3*d*, **c** Fe 2*p*, **d** Zn 2*p*, **e** O 1*s* regions over 10 etching levels.

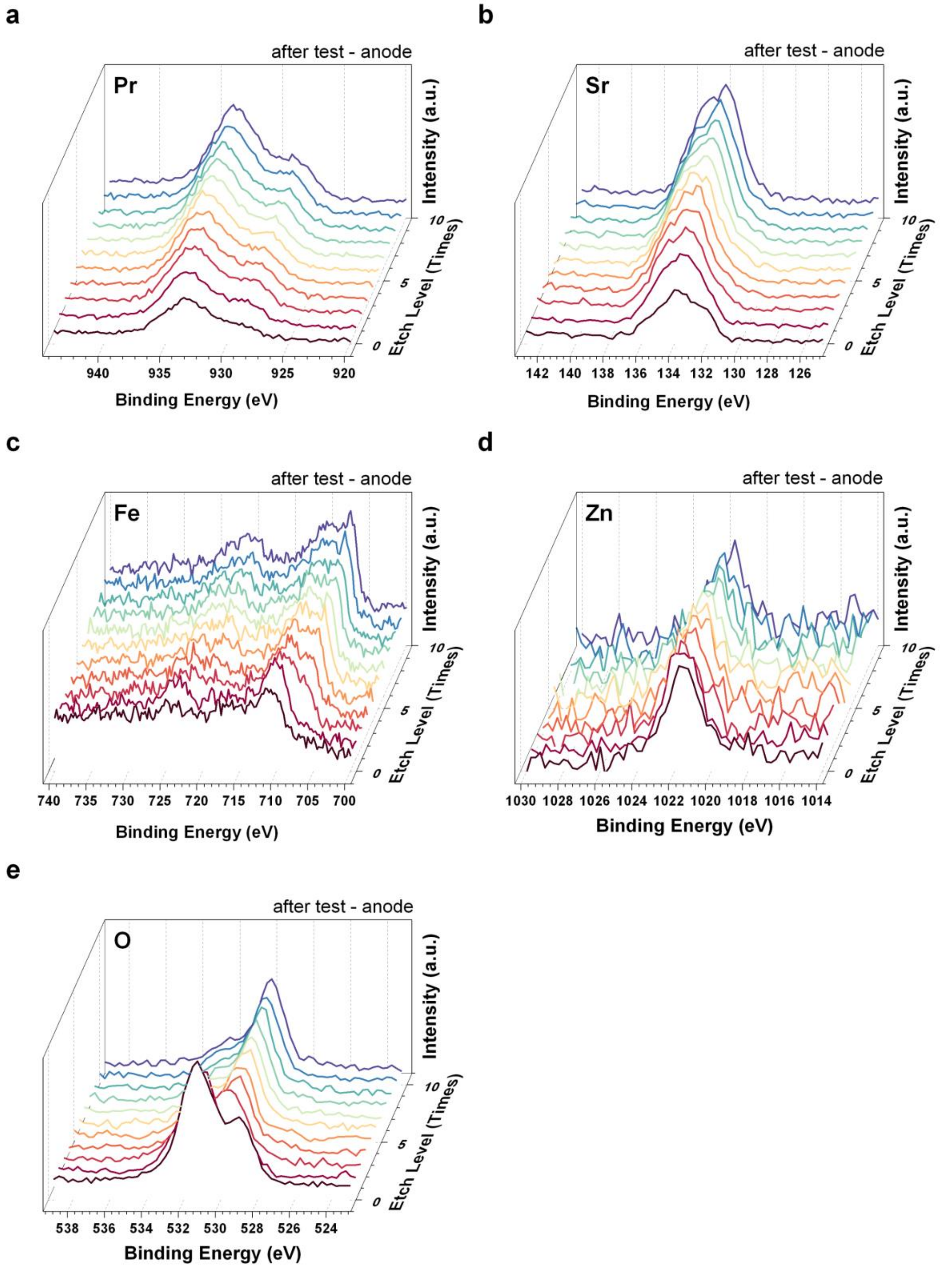

Supplementary Fig. 46 | XPS depth profiles of the D-PSFZ anode after the full-cell testing. XPS spectra for **a** Pr 3*d*, **b** Sr 3*d*, **c** Fe 2*p*, **d** Zn 2*p*, **e** O 1*s* regions over 10 etching levels.

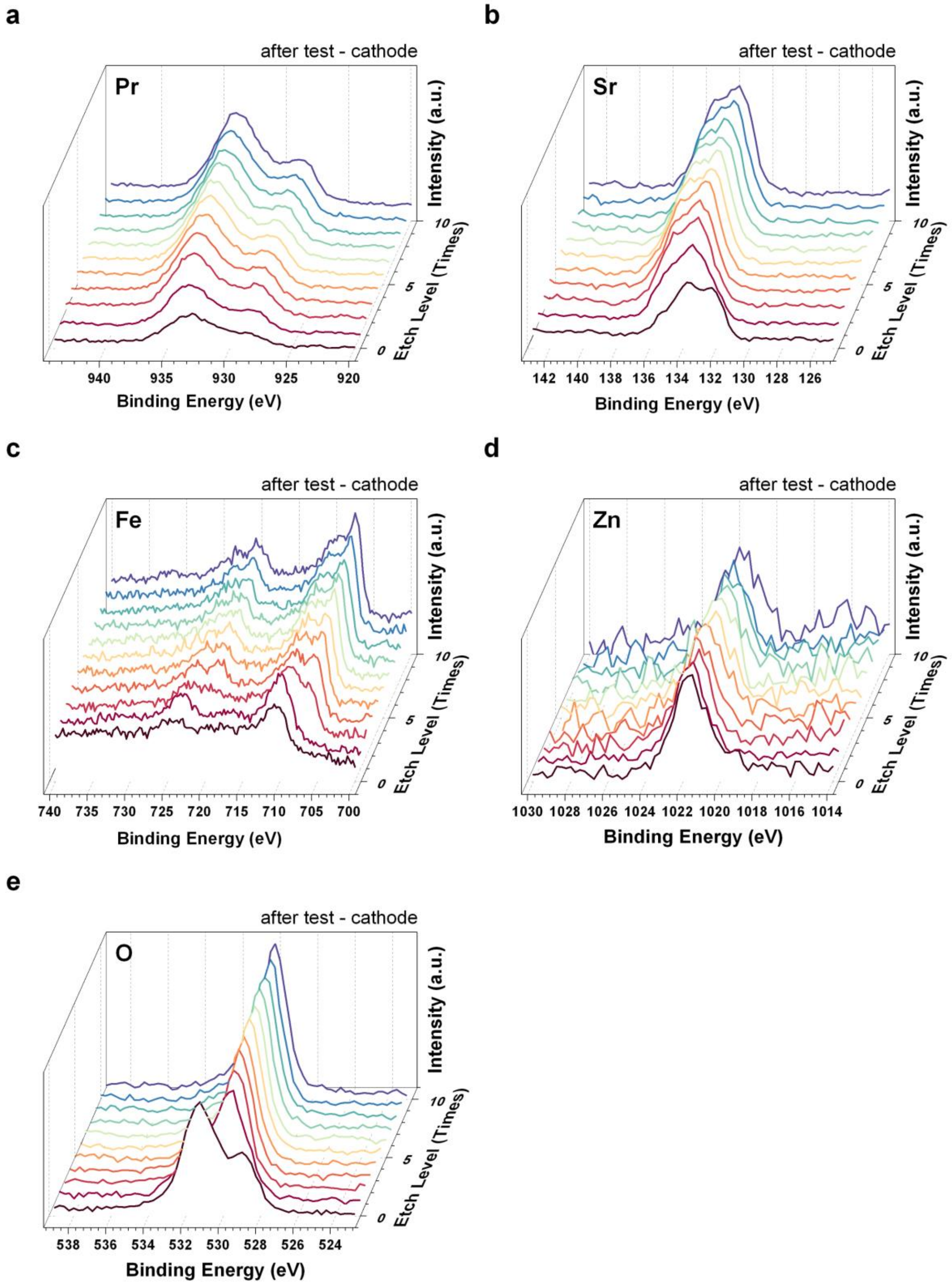

Supplementary Fig. 47 | XPS depth profiles of the D-PSFZ cathode after the full-cell testing. XPS spectra for **a** Pr 3*d*, **b** Sr 3*d*, **c** Fe 2*p*, **d** Zn 2*p*, **e** O 1*s* regions over 10 etching levels.

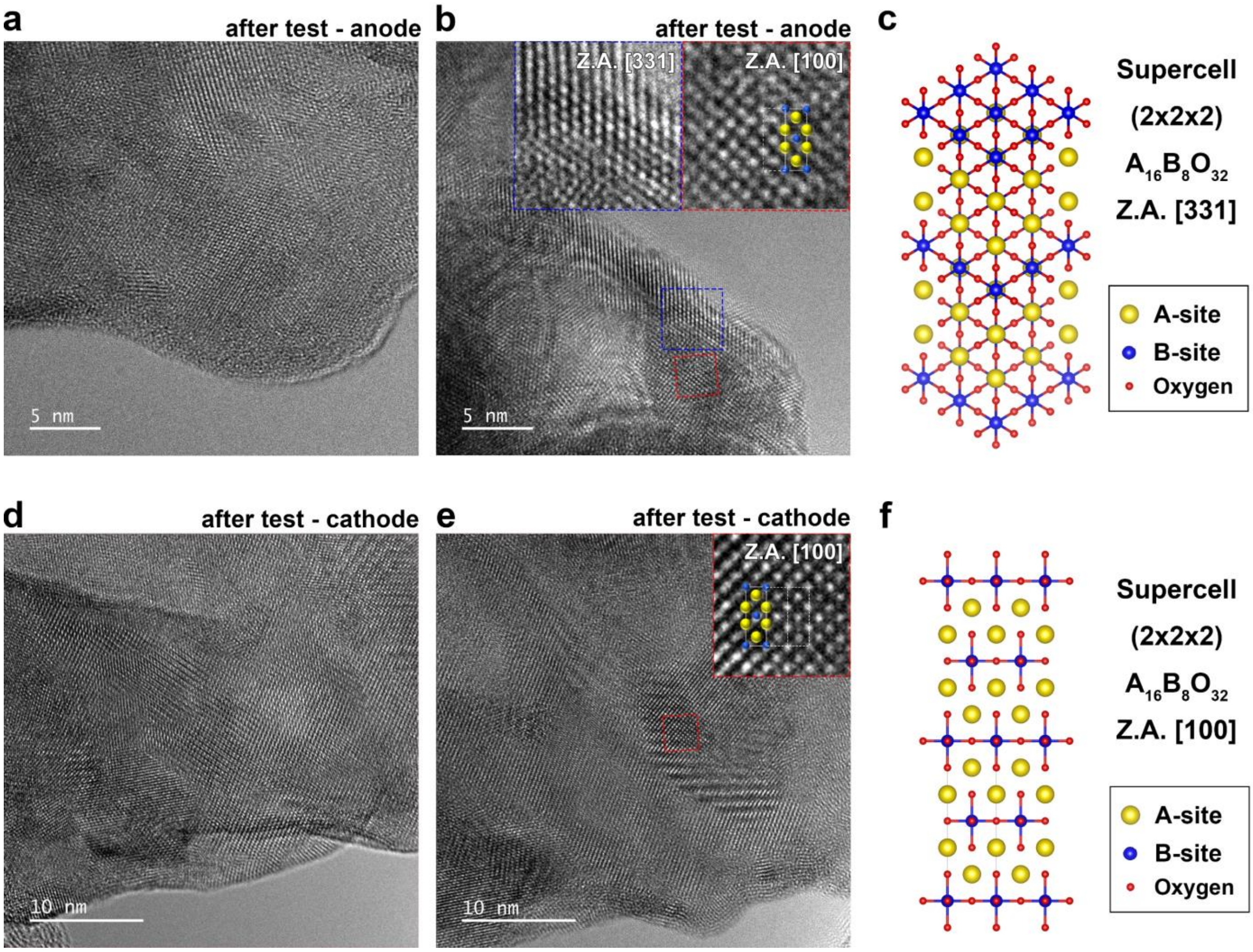

Supplementary Fig. 48 | HR-TEM images of the D-PSFZ electrodes that used for the stability testing of the D-PSFZ || D-PSFZ full-cell in the 2.0 M $KHCO_3/K_2CO_3$ condition. a and **b** HR-TEM images of the anode and enlarged images as insets. **d** and **e** HR-TEM images of the cathode and an enlarged image as an inset. **c** and **f** Schematic models of RP-perovskite supercells ($A_{16}O_8O_{32}$) aligned with the zone axis of [331] and [100] respectively.

Supplementary Note 1 | Calculation details for the FE of the D-PSFZ || D-PSFZ cell of **Supplementary Movie 1.**

Dye decomposition experiments were performed during an overall H_2O_2 electrosynthesis under the D-PSFZ || D-PSFZ configuration (**Supplementary Movie 1**). The beaker cell reactor shown in **Figure 6a** is connected to the inlet flow pumps (single pass) of the electrolyte reservoir (*i.e.*, 2.0 M $KHCO_3/K_2CO_3$) at the flow rate of 15 sccm. The outlet electrolyte was directly supplied to the dye solution (*i.e.*, 2.5 μmol $KMnO_4$ in sulfuric acid). The reaction takes approximately 20 seconds, corresponding to 5 mL of the electrolyte outlet to remove the dye. Since two permanganate ions react to five hydrogen peroxide molecules, the consumed H_2O_2 corresponds to 6.25 μmol for 20 seconds (*i.e.*, H_2O_2 production rate: 18.75 μmol min^{-1}). The electrolysis proceeds at 50 mA cm^{-2} on 1 cm^2 electrodes of the cathode and the anode, the H_2O_2 yield can be calculated as follows. Current: 50 mA = 50 mC s^{-1} = 0.031088 $mmol_{electrons}$ min^{-1} = 0.015544 $mmol_{H2O2}$ min^{-1} = 15.544 μmol_{H2O2} min^{-1}. Since the FE can be calculated from the following equation and the cathodic FE was experimentally determined to be 95 %.

$$\text{FE (\%)} = \frac{\text{charge used for 2e reaction/2 (C)}}{\text{charge used for 2e reaction/2 + charge used for 4e reaction/4 (C)}} \times 100\ (\%)$$

The cathodic H_2O_2 yield corresponds to 14.0636 μmol_{H2O2} min^{-1}. Then anodic H_2O_2 yield can be determined to be 4.6864 μmol_{H2O2} min^{-1} (∵ 18.750 − 14.0636 μmol_{H2O2} min^{-1}) and thus the anodic FE is 46.4 %. Thus the total FE is ~141 % that can be calculated as the sum of the anodic FE and the cathodic FE.

Supplementary Table 1 | Summary of point spectrum in Supplementary Fig. 3.

Spectrum 1	Line type	k factor	Adsorption correction	Weight %	Atomic %
O	K series	2.007	1.00	20.31	61.19
Fe	K series	1.155	1.00	10.85	9.36
Zn	K series	1.310	1.00	2.64	1.95
Sr	L series	1.727	1.00	23.40	12.87
Pr	L series	2.032	1.00	42.80	14.64
Sub Total:				100.00	100.00
Spectrum 2	Line type	k factor	Adsorption correction	Weight %	Atomic %
O	K series	2.007	1.00	20.67	61.55
Fe	K series	1.155	1.00	11.00	9.39
Zn	K series	1.310	1.00	3.12	2.28
Sr	L series	1.727	1.00	22.99	12.50
Pr	L series	2.032	1.00	42.22	14.28
Sub Total:				100.00	100.00

Supplementary Table 2 | Lattice parameters of D-PSFZ calculated by Rietveld refinement.

Space group	*a* (Å)	*b* (Å)	*c* (Å)	*V* (Å^3)	R_{wp}	R_p
I4/mmm	3.84	3.84	12.74	187.86	15.7	15.9

Supplementary Table 3 | XPS peak deconvolution results of PSF oxide based on the relative area of each deconvolution peaks shown in **Supplementary Fig. 4**. Note Sr cation has the fixed transition state as 2+.

Pr 3*d*		Fe 2*p*			O 1*s*					
Pr^{3+} (%)	Pr^{4+} (%)	Fe^{2+} (%)	Fe^{3+} (%)	Fe^{2+}/Fe^{3+} (a.u.)	O*l* (%)	O*v* (%)	O*ads* (%)	O*w* (%)	O*v*/O*l* (a.u.)	O*ads*/O*l* (a.u.)
83.4	16.6	71.3	28.7	2.48	51.4	1.65	40.5	6.5	0.032	0.788

Supplementary Table 4 | XPS peak deconvolution results of D-PSFZ oxide based on the relative area of each deconvolution peaks shown in **Supplementary Fig. 5**. Note Sr and Zn cations have the fixed transition states as 2+.

Pr 3*d*		Fe 2*p*			O 1*s*					
Pr^{3+} (%)	Pr^{4+} (%)	Fe^{2+} (%)	Fe^{3+} (%)	Fe^{2+}/Fe^{3+} (a.u.)	O*l* (%)	O*v* (%)	O*ads* (%)	O*w* (%)	O*v*/O*l* (a.u.)	O*ads*/O*l* (a.u.)
82.2	17.8	66.4	33.6	1.97	21.9	4.2	33.8	5.1	0.194	1.546

Supplementary Table 5 | Data sheet of the permanganate titration to calculate H_2O_2 yield and Faradaic efficiency (%) during 2e-ORR in 0.1 M $KHCO_3$(aq) for D-PSFZ. The H_2O_2 concentration was accumulated by chronoamperometric measurement for 10 min at various potential range between 0.05 to 0.45 V *vs.* RHE (For the titration, 0.5 mL of the product solution was taken).

	0.45 V	0.40 V	0.35 V	0.30 V	0.25 V	0.20 V	0.15 V	0.10 V	0.05 V
Charge (mC)	530	975.8	1943	2497.4	4290	4556.9	6430	7076	8620
Titrant volume (μL)	9	16.6	33	43	74	78	110	122	148.5
H_2O_2 conc. (mg L^{-1})	7.65	14.12	28.06	36.57	62.93	66.33	93.54	103.75	126.28
FE (%)	98.31	98.48	98.32	99.68	99.86	99.09	99.04	99.81	99.73
n	2.03	2.03	2.03	2.01	2.00	2.02	2.02	2.00	2.01

Supplementary Table 6 | Data sheet of the permanganate titration to calculate H_2O_2 yield and Faradaic efficiency (%) during 2e-ORR in 0.5 M $KHCO_3$(aq) for D-PSFZ. The H_2O_2 concentration was accumulated by chronoamperometric measurement for 15 min at various potential range between 0.05 to 0.45 V *vs.* RHE (For the titration, 0.3 mL of the product solution was taken).

	0.45 V	0.40 V	0.35 V	0.30 V	0.25 V	0.20 V	0.15 V	0.10 V	0.05 V
Charge (mC)	1127	3056	5859	11200	14123	22445	24162	28190	33040
Titrant volume (μL)	11.6	31.5	60.5	116	146	230	250	290	340
H_2O_2 conc. (mg L^{-1})	16.44	44.64	85.64	164.41	206.92	325.98	354.32	414.1	481.88
FE (%)	99.31	99.45	99.63	99.93	99.74	98.87	99.83	99.43	99.29
n	2.01	2.01	2.01	2.00	2.01	2.02	2.00	2.01	2.01

Supplementary Table 7 | Data sheet of the permanganate titration to calculate H_2O_2 yield and Faradaic efficiency (%) during 2e-ORR in 1.0 M $KHCO_3$(aq) for D-PSFZ. The H_2O_2 concentration was accumulated by chronoamperometric measurement for 10 min at various potential range between 0.05 to 0.45 V *vs.* RHE (For the titration, 0.5 mL of the product solution was taken).

	0.45 V	0.35 V	0.25 V	0.15 V	0.05 V
Charge (mC)	2187	10663	18133	28393	38608
Titrant volume (μL)	37.5	183	301	480	650
H_2O_2 conc. (mg L^{-1})	31.89	155.62	255.76	408.18	552.74
FE (%)	99.26	99.35	96.10	97.87	97.46
n	2.01	2.01	2.08	2.04	2.05

Supplementary Table 8 | Data sheet of the permanganate titration to calculate H_2O_2 yield and Faradaic efficiency (%) during 2e-ORR in 2.0 M $KHCO_3$(aq) for D-PSFZ. The H_2O_2 concentration was accumulated by chronoamperometric measurement for 10 min at various potential range between 0.05 to 0.45 V *vs.* RHE (For the titration, 0.3 mL of the product solution was taken, *for the 0.05 V condition, 0.2 mL of the solution was taken).

	0.45 V	0.35 V	0.25 V	0.15 V	0.05 V
Charge (mC)	1936	11990	24085	37264	54507
Titrant volume (μL)	20	122	245	380	345* (0.2 mL)
H_2O_2 conc. (mg L^{-1})	28.35	172.91	347.24	538.57	733.45
FE (%)	99.84	99.08	99.07	99.19	95.62
n	2.00	2.02	2.02	2.02	2.09

Supplementary Table 9 | Data sheet of the permanganate titration to calculate H_2O_2 yield and Faradaic efficiency (%) during 2e-ORR in saturated $KHCO_3$(aq) for D-PSFZ. The H_2O_2 concentration was accumulated by chronoamperometric measurement for 10 min at various potential range between 0.05 to 0.45 V *vs.* RHE (For the titration, 0.3 mL of the product solution was taken).

	0.45 V	0.35 V	0.25 V	0.15 V	0.05 V
Charge (mC)	3600	13149	21136	27266	36400
Titrant volume (μL)	27	92	160	195	240
H_2O_2 conc. (mg L^{-1})	38.27	130.39	226.77	276.37	340.15
FE (%)	83.96	80.60	84.42	81.66	77.97
n	2.32	2.39	2.31	2.37	2.44

Supplementary Table 10 | Data sheet of the permanganate titration to calculate H_2O_2 yield and Faradaic efficiency (%) during 2e-ORR in 0.1 M $KHCO_3$(aq) for R-PSFZ. (Electrolyte: 12 mL, for the titration, 0.5 mL of the product solution was taken).

	0.45 V	0.35 V	0.25 V	0.15 V	0.05 V
Charge (mC)	400	1058	1844	2621	3542
Titrant volume (μL)	4	13	28	43	60
H_2O_2 conc. (mg L^{-1})	3.40	11.05	23.81	36.57	51.02
FE (%)	73.33	83.13	93.56	97.42	99.02
n	2.53	2.34	2.13	2.05	2.02

Supplementary Table 11 | Data sheet of the permanganate titration to calculate H_2O_2 yield and Faradaic efficiency (%) during 2e-ORR in 0.5 M $KHCO_3$(aq) for R-PSFZ. (Electrolyte: 12 mL, for the titration, 0.5 mL of the product solution was taken).

	0.45 V	0.35 V	0.25 V	0.15 V	0.05 V
Charge (mC)	828.5	3104	5591	8486	11050
Titrant volume (μL)	12	44	89	141	180
H_2O_2 conc. (mg L^{-1})	10.20	37.42	75.68	119.90	153.07
FE (%)	91.22	90.15	95.92	98.06	97.07
n	2.18	2.20	2.08	2.04	2.06

Supplementary Table 12 | Data sheet of the permanganate titration to calculate H_2O_2 yield and Faradaic efficiency (%) during 2e-ORR in 1.0 M $KHCO_3$(aq) for R-PSFZ. (Electrolyte: 12 mL, for the titration, 0.5 mL of the product solution was taken).

	0.45 V	0.35 V	0.25 V	0.15 V	0.05 V
Charge (mC)	852	3548	7345	127440	17490
Titrant volume (μL)	14.5	46	100	195	270
H_2O_2 conc. (mg L^{-1})	12.33	39.12	85.04	165.82	229.60
FE (%)	99.26	85.75	88.15	16.27	94.39
n	2.01	2.28	2.24	3.67	2.11

Supplementary Table 13 | Data sheet of the permanganate titration to calculate H_2O_2 yield and Faradaic efficiency (%) during 2e-ORR in 2.0 M $KHCO_3$(aq) for R-PSFZ. (Electrolyte: 12 mL, for the titration, 0.5 mL of the product solution was taken).

	0.45 V	0.35 V	0.25 V	0.15 V	0.05 V
Charge (mC)	1202	5596	11680	17460	24720
Titrant volume (μL)	13	56	150	285	365
H_2O_2 conc. (mg L^{-1})	11.05	47.62	127.56	242.36	310.39
FE (%)	77.01	73.36	85.29	97.17	92.17
n	2.46	2.53	2.29	2.06	2.16

Supplementary Table 14 | Data sheet of the permanganate titration to calculate H_2O_2 yield and Faradaic efficiency (%) during 2e-ORR in 2.0 M $KHCO_3$(aq) for Q-PSFZ. (Electrolyte: 12 mL, for the titration, 0.5 mL of the product solution was taken).

	0.45 V	0.35 V	0.25 V	0.15 V	0.05 V
Charge (mC)	2612	8424	15210	18180	21940
Titrant volume (μL)	42	126	204	258	312
H_2O_2 conc. (mg L^{-1})	35.72	107.15	173.48	219.40	265.32
FE (%)	96.42	92.81	87.42	90.20	90.31
n	2.07	2.14	2.25	2.20	2.19

Supplementary Table 15 | Data sheet of the permanganate titration to calculate H_2O_2 yield and Faradaic efficiency (%) during 2e-ORR in 0.1 M KOH(aq) for D-PSFZ. (Electrolyte: 12 mL, for the titration, 0.5 mL of the product solution was taken).

	0.45 V	0.35 V	0.25 V	0.15 V	0.05 V
Charge (mC)	2268	3070	3997	5350	4963
Titrant volume (μL)	39	52	68	91	85
H_2O_2 conc. (mg L^{-1})	33.16	44.22	57.83	77.38	72.28
FE (%)	99.77	99.02	99.24	99.23	99.57
n	2.00	2.02	2.02	2.02	2.01

Supplementary Table 16 | Data sheet of the permanganate titration to calculate H_2O_2 yield and Faradaic efficiency (%) during 2e-WOR in 0.1 M $KHCO_3$(aq) for D-PSFZ. The H_2O_2 concentration was accumulated by chronoamperometric measurement for 30 min at various potential range between 0.05 to 0.45 V *vs*. RHE (For the titration, 0.5 mL of the product solution was taken).

	2.05 V	2.10 V	2.15 V	2.20 V	2.25 V	2.30 V	2.35 V
Charge (mC)	509.26	969.4	1201.5	1578.8	1754	2124.2	2586.6
Titrant volume (μL)	3	6	7	8	9	9	10
H_2O_2 conc. (mg L^{-1})	2.55	5.10	5.95	6.80	7.65	7.65	8.50
FE (%)	50.86	52.76	50.44	45.36	45.80	39.40	36.57
n	2.98	2.94	2.99	3.09	3.08	3.21	3.27

Supplementary Table 17 | Data sheet of the permanganate titration to calculate H_2O_2 yield and Faradaic efficiency (%) during 2e-WOR in 0.5 M $KHCO_3$(aq) for D-PSFZ. The H_2O_2 concentration was accumulated by chronoamperometric measurement for 30 min at various potential range between 0.05 to 0.45 V *vs*. RHE (For the titration, 0.5 mL of the product solution was taken).

	2.05 V	2.10 V	2.15 V	2.20 V	2.25 V	2.30 V	2.35 V
Charge (mC)	951.2	1631	2257.1	3060.8	3998.2	5424	6832.1
Titrant volume (μL)	9	17	26	25	26	35	40
H_2O_2 conc. (mg L^{-1})	7.65	14.46	22.11	21.26	22.11	29.76	34.02
FE (%)	70.78	75.27	80.02	64.20	54.70	54.40	50.62
n	2.58	2.49	2.40	2.72	2.91	2.91	2.99

Supplementary Table 18 | Data sheet of the permanganate titration to calculate H_2O_2 yield and Faradaic efficiency (%) during 2e-WOR in 1.0 M $KHCO_3$(aq) for D-PSFZ. The H_2O_2 concentration was accumulated by chronoamperometric measurement for 30 min at various potential range between 0.05 to 0.45 V *vs*. RHE (For the titration, 0.5 mL of the product solution was taken).

	2.05 V	2.10 V	2.15 V	2.20 V	2.25 V	2.30 V	2.35 V
Charge (mC)	1435.7	3709.7	3931.8	5229.5	7808.9	8677.3	13086
Titrant volume (μL)	15	38	35	36	41	43	62
H_2O_2 conc. (mg L^{-1})	12.76	32.31	29.76	30.61	34.87	36.57	52.72
FE (%)	75.37	74.45	68.01	56.99	46.63	44.59	43.05
n	2.49	2.51	2.64	2.86	3.07	3.11	3.14

Supplementary Table 19 | Data sheet of the permanganate titration to calculate H_2O_2 yield and Faradaic efficiency (%) during 2e-WOR in 2.0 M $KHCO_3$(aq) for D-PSFZ. The H_2O_2 concentration was accumulated by chronoamperometric measurement for 15 min at various potential range between 0.05 to 0.45 V *vs*. RHE (For the titration, 0.5 mL of the product solution was taken).

	2.05 V	2.10 V	2.15 V	2.20 V	2.25 V	2.30 V	2.35 V
Charge (mC)	2631	5642	6595.7	10003	11507	13687.7	17807
Titrant volume (μL)	26	54	59	65	58	64	77
H_2O_2 conc. (mg L^{-1})	22.11	45.92	50.17	55.27	49.32	54.42	65.48
FE (%)	72.78	71.31	68.23	54.67	45.18	42.61	40.04
n	2.54	2.57	2.64	2.91	3.10	3.15	3.20

Supplementary Table 20 | Data sheet of the permanganate titration to calculate H_2O_2 yield and Faradaic efficiency (%) during 2e-WOR in 1.0 M $KHCO_3/K_2CO_3$(aq) for D-PSFZ. (Electrolyte: 90 mL, for the titration, 0.5 mL of the product solution was taken).

	2.10 V	2.20 V	2.30 V	2.40 V	2.50 V	2.60 V	2.70 V
Charge (mC)	5549	10120	18870	27040	36790	49490	60440
Titrant volume (μL)	5	8	16	26	36	41	51
H_2O_2 conc. (mg L^{-1})	4.25	6.80	13.61	22.11	30.61	34.87	43.37
FE (%)	56.24	51.10	53.82	58.90	59.64	52.91	53.63
n	2.88	2.98	2.92	2.82	2.81	2.94	2.93

Supplementary Table 21 | Data sheet of the permanganate titration to calculate H_2O_2 yield and Faradaic efficiency (%) during 2e-WOR in 2.0 M $KHCO_3/K_2CO_3$(aq) for D-PSFZ. (Electrolyte: 75 mL, for the titration, 0.5 mL of the product solution was taken, *only for 2.10 V, 1.0 mL of the solution was taken to avoid error)

	2.10 V	2.20 V	2.30 V	2.40 V	2.50 V	2.60 V	2.70 V
Charge (mC)	3030	5428	9351	14295	20572	22558	29567
Titrant volume (μL)	6.5	5.5	9	15	25	29	29
H_2O_2 conc. (mg L^{-1})	2.76	4.68	7.65	12.76	21.26	24.66	24.66
FE (%)	55.92	53.65	51.66	55.04	61.08	63.49	52.39
n	2.88	2.93	2.97	2.90	2.78	2.73	2.95

Supplementary Table 22 | Data sheet of the permanganate titration to calculate H_2O_2 yield and Faradaic efficiency (%) during 2e-ORR in 1.0 M $KHCO_3/K_2CO_3$(aq) for D-PSFZ. (Electrolyte: 12 mL, for the titration, 0.5 mL of the product solution was taken).

	0.45 V	0.35 V	0.25 V	0.15 V	0.05 V
Charge (mC)	5627	10100	15410	23790	26630
Titrant volume (μL)	95	173	260	370	410
H_2O_2 conc. (mg L^{-1})	80.79	147.11	221.10	314.64	348.65
FE (%)	98.86	99.58	98.82	94.76	94.25
n	2.02	2.01	2.02	2.10	2.11

Supplementary Table 23 | Data sheet of the permanganate titration to calculate H_2O_2 yield and Faradaic efficiency (%) during 2e-ORR in 2.0 M $KHCO_3/K_2CO_3$(aq) for D-PSFZ. (Electrolyte: 12 mL, for the titration, 0.5 mL of the product solution was taken)

	0.45 V	0.35 V	0.25 V	0.15 V	0.05 V
Charge (mC)	7264	14640	16730	29160	36600
Titrant volume (μL)	120	245	270	430	550
H_2O_2 conc. (mg L^{-1})	102.05	208.34	229.60	365.66	467.71
FE (%)	97.77	98.42	96.60	92.11	93.05
n	2.04	2.03	2.07	2.16	2.14

Supplementary Table 24 | Data sheet of the permanganate titration to calculate H_2O_2 yield and Faradaic efficiency (%) during the overall H_2O_2 electrolysis in 0.5 M $KHCO_3$(aq) for D-PSFZ || D-PSFZ. The H_2O_2 concentration was accumulated by chronoamperometric measurement for 10 min at the cell potential range between 1.60 to 2.20 V and 16 mL of electrolyte was used (For the titration, 0.5 mL of the product solution was taken, *only for 1.60 V, 0.4 mL of the solution was taken.).

	1.60 V	1.70 V	1.80 V	1.90 V	2.20 V	2.10 V	2.20 V
Charge (mC)	724	1364.7	2114.6	3031.7	3897.7	4655.9	5588.2
Titrant volume (μL)	10*	9.5	16	22	28	33	39
H_2O_2 conc. (mg L^{-1})	10.63	20.20	34.02	46.77	59.53	70.16	82.91
FE (%)	151.14	152.29	163.86	158.17	156.76	154.87	152.65

Supplementary Table 25 | Data sheet of the permanganate titration to calculate H_2O_2 yield and Faradaic efficiency (%) during the overall H_2O_2 electrolysis in 1.0 M $KHCO_3$(aq) for D-PSFZ || D-PSFZ. The H_2O_2 concentration was accumulated by chronoamperometric measurement for 10 min at the cell potential range between 1.60 to 2.20 V and 16 mL of electrolyte was used (For the titration, 0.2 mL of the product solution was taken, *only for 1.60 V, 0.4 mL of the solution was taken.).

	1.60 V	1.70 V	1.80 V	1.90 V	2.20 V	2.10 V	2.20 V
Charge (mC)	952	1750	2823	4087	4151	5786	7397
Titrant volume (μL)	14*	13	21	30	30	41	50
H_2O_2 conc. (mg L^{-1})	14.88	27.64	44.64	63.78	63.78	87.16	106.30
FE (%)	159.99	161.39	161.58	159.76	157.61	154.84	148.09

Supplementary Table 26 | Data sheet of the permanganate titration to calculate H_2O_2 yield and Faradaic efficiency (%) during the overall H_2O_2 electrolysis in 1.0 M $KHCO_3/K_2CO_3$(aq) for D-PSFZ || D-PSFZ. (Electrolyte: 12 mL, for the titration, 0.5 mL of the product solution was taken)

	1.70 V	1.80 V	1.90 V	2.00 V	2.10 V	2.20 V	2.30 V
Charge (mC)	2871	5555.4	8351	11717	13828.5	14025.3	18772
Titrant volume (μL)	8	15	22	32	42	40	52
H_2O_2 conc. (mg L^{-1})	6.80	12.76	18.71	27.21	35.72	34.02	44.22
FE (%)	151.56	147.15	143.59	148.75	163.01	154.74	150.74

Supplementary Table 27 | Data sheet of the permanganate titration to calculate H_2O_2 yield and Faradaic efficiency (%) during the stability test toward 2e-ORR in 1.0 M $KHCO_3$(aq) for D-PSFZ shown in **Figure 3d**. (Electrolyte flow rate: 10 sccm, for the titration, 0.5 mL of the product solution was taken)

Time (h)	0.5	24	48	72	96	120	144	168	192	216	240
Current (mA)	66.63	70.66	71.48	71.63	71.63	72.59	71.53	73.28	73.07	74.50	68.98
Titrant volume (μL)	80	85	86	83	87	86	82	82	79	85	85
H_2O_2 conc. (mg L^{-1})	68.03	72.28	73.13	70.58	73.98	73.13	69.73	69.73	67.18	72.28	72.28
FE (%)	98.27	98.36	98.37	96.49	98.85	97.60	95.95	94.75	93.04	95.72	99.57
Time (h)	264	288	312	336	360	384	408	432	456	480	499
Current (mA)	69.32	66.35	65.30	65.92	65.72	61.41	67.56	71.80	69.78	72.32	66.13
Titrant volume (μL)	84	81	79	79	79	73	76	82	82	82	79
H_2O_2 conc. (mg L^{-1})	71.43	68.88	67.18	67.18	67.18	62.08	64.63	69.73	69.73	69.73	67.18
FE (%)	98.73	99.10	98.65	98.17	98.32	97.77	95.01	95.77	97.19	95.41	98.02

Supplementary Table 28 | Data sheet of the permanganate titration to calculate H_2O_2 yield and Faradaic efficiency (%) during the stability test toward 2e-WOR in 1.0 M $KHCO_3/K_2CO_3$(aq) for D-PSFZ shown in **Figure 4d**. (Electrolyte flow rate: 15 sccm, chronopotentiometry at 70 mA cm^{-2} on 1 cm^2 electrode)

Time (h)	0.5	22.85	43.2	68.2	88.45	109.6	135.5	152.2	165.27	180.45	197.5
Analyte volume (mL)	0.5	0.5	0.5	1.0	1.0	1.0	1.0	1.0	1.0	1.0	1.0
Titrant volume (μL)	22	21	20	43	39	39	41	42	40	41	40
H_2O_2 conc. (mg L^{-1})	18.71	17.86	17.01	18.28	16.58	16.58	17.43	17.86	17.01	17.43	17.01
FE (%)	55.00	53.16	51.28	54.08	50.32	50.32	52.23	53.16	51.28	52.23	51.28

Supplementary Table 29 | Data sheet of the permanganate titration to calculate H_2O_2 yield and Faradaic efficiency (%) during the stability test toward the overall H_2O_2 electrosynthesis in 2.0 M $KHCO_3/K_2CO_3$(aq) shown in **Figure 6c**. (Electrolyte flow rate: 15 sccm, at 50 mA cm^{-2} on 1 cm^2 electrodes, cathode FE = 95 %)

Time (h)	0.5	9	20	32	44	56	69	80	89	99
Analyte volume (mL)	0.25	0.25	0.25	0.5	0.5	0.8	0.4	0.25	0.5	1.0
Titrant volume (mL)	26	24	26	51	50	81	42	26	51	101
H_2O_2 conc. (mg L^{-1})	44.22	40.82	44.22	43.37	42.52	43.05	44.64	44.22	43.37	42.94
Cathode yield (mg L^{-1})	31.88	31.88	31.88	31.88	31.88	31.88	31.88	31.88	31.88	31.88
Anode yield (mg L^{-1})	12.34	8.94	12.34	11.49	10.64	11.17	12.77	12.34	11.49	11.07
Anodic FE (%)	51.88	40.48	51.88	49.19	46.39	48.15	53.20	51.88	49.19	47.80
Total FE (%)	146.88	135.48	146.88	144.19	141.39	143.15	148.20	146.88	144.19	142.80

Supplementary Table 30 | Comparison of 2e-ORR activities of D-PSFZ to recently published articles measured at high η region. [a] $\eta = 0.6$ V indicates where $E = 0.10$ V *vs*. RHE since $E^o(O_2/H_2O_2) = 0.70$ V.

Catalysts	Electrolyte	Ionic strength	Testing condition	J (mA cm^{-2}) at $\eta = 0.6$ V [a]	Corresponding FE (%)	Year	Ref.
D-PSFZ	Neutral ($KHCO_3$)	0.1 M	RRDE	3 mA cm^{-2}	~90 %	*This work*	
D-PSFZ	Neutral ($KHCO_3$)	0.1 M	H-cell	11 mA cm^{-2}	~99 %	*This work*	
D-PSFZ	Neutral ($KHCO_3$)	0.5 M	H-cell	32 mA cm^{-2}	~99 %	*This work*	
D-PSFZ	Neutral ($KHCO_3$)	1.0 M	H-cell	70 mA cm^{-2} ($\eta = 0.65$ V)	~97.5 %	*This work*	
D-PSFZ	Neutral ($KHCO_3$)	2.0 M	H-cell	80 mA cm^{-2} ($\eta = 0.65$ V)	~95 %	*This work*	
D-PSFZ	Weak base ($KHCO_3/K_2CO_3$)	1.0 M	H-cell	90 mA cm^{-2} ($\eta = 0.65$ V)	~94 %	*This work*	
D-PSFZ	Weak base ($KHCO_3/K_2CO_3$)	2.0 M	H-cell	120 mA cm^{-2} ($\eta = 0.65$ V)	~93 %	*This work*	
D-PSFZ	Base (KOH)	0.1 M	H-cell	18 mA cm^{-2} ($\eta = 0.65$ V)	~99 %	*This work*	
Pt-Hg	Acid ($HClO_4$)	0.1 M	RRDE	~3.6 mA cm^{-2}	~90 %	2013	S1
Au-Pt-Ni	Base (KOH)	0.1 M	RRDE	~2.7 mA cm^{-2}	~90 %	2016	S2
FPC-800	Acid (H_2SO_4)	0.05 M	RRDE	~2.5 mA cm^{-2}	~82 %	2018	S3
g-N-CNHs	Acid (H_2SO_4)	0.1 M	RRDE	3.6 mA cm^{-2} ($\eta = 0.3$ V)	98 %	2018	S4
g-N-CNHs	Neutral (PBS)	0.1 M	RRDE	2.3 mA cm^{-2} ($\eta = 0.25$ V)	90 %	2018	S4
g-N-CNHs	Base (KOH)	0.1 M	RRDE	1.79 mA cm^{-2} ($\eta = 0.05$ V)	63 %	2018	S4
O-CNTs	Base (KOH)	0.1 M	RRDE	2.80 mA cm^{-2} ($\eta = 0.3$ V)	~88 %	2018	S5
O-CNTs	Neutral (PBS)	0.1 M	RRDE	2.80 mA cm^{-2}	~82 %	2018	S5
MesoC	Base (KOH)	0.1 M	RRDE	~4 mA cm^{-2} ($\eta = 0.5$ V)	~65 %	2018	S6
NCMK3IL50-800T	Acid (H_2SO_4)	0.5 M	RRDE	1.5 mA cm^{-2}	92 %	2018	S7
h-Pt_1-CuS_x	Acid ($HClO_4$)	0.1 M	RRDE	2.8 mA cm^{-2}	92 %	2019	S8
CoS_2	Acid (H_2SO_4)	0.05 M	RRDE	2 mA cm^{-2}	60 %	2019	S9
Fe-CNT	Base (KOH)	0.1 M	RRDE	3.2 mA cm^{-2} ($\eta = 0.3$ V)	~76 %	2019	S10
Fe-CNT	Neutral (PBS)	0.1 M	RRDE	3.2 mA cm^{-2}	~80 %	2019	S10
Amorphous Pd	Acid ($HClO_4$)	0.1 M	RRDE	3 mA cm^{-2}	95 %	2019	S11
oxo-G/NH_3H_2O	Base (KOH)	0.1 M	RRDE	~3 mA cm^{-2}	84 %	2019	S12
C_{60}-CNT hybrid	Acid (H_2SO_4)	0.05 M	RRDE	5 mA cm^{-2}	72 %	2019	S13

Co-N-C	Acid (H_2SO_4)	0.05 M	RRDE	3 mA cm^{-2}	80 %	2019	S14
Co-N-C	Neutral (K_2SO_4)	0.1 M	RRDE	3.8 mA cm^{-2}	55 %	2019	S14
Co-N-C	Base (KOH)	0.1 M	RRDE	3.8 mA cm^{-2}	65 %	2019	S14
O-C(Al)	Base (NaOH)	0.1 M	RRDE	2.9 mA cm^{-2} (η = 0.3 V)	93 %	2020	S15
$Pd^{\delta+}$-OCNT	Acid ($HClO_4$)	0.1 M	RRDE	2.1 mA cm^{-2}	90 %	2020	S16
MCHS-9:1	Acid (H_2SO_4)	0.5 M	RRDE	2.5 mA cm^{-2}	70 %	2020	S17
MCHS-9:1	Neutral (PBS)	0.1 M	RRDE	3 mA cm^{-2} (η = 0.4 V)	85 %	2020	S17
MCHS-9:1	Base (KOH)	0.1 M	RRDE	3 mA cm^{-2} (η = 0.3 V)	55 %	2020	S17
Co_1-NG(O)	Base (KOH)	0.1 M	RRDE	2.9 mA cm^{-2}	80 %	2020	S18
HE-CoN@CNTs	Acid ($HClO_4$)	0.1 M	RRDE	3 mA cm^{-2} (η = 0.5 V)	90 %	2020	S19
MOF NSs-300	Base (KOH)	0.1 M	RRDE	3.5 mA cm^{-2} (η = 0.5 V)	80 %	2020	S20
PtP_2 NCs	Acid ($HClO_4$)	0.1 M	RRDE	3 mA cm^{-2}	91 %	2020	S21
Co-NC	Acid ($HClO_4$)	0.1 M	RRDE	3 mA cm^{-2}	78 %	2020	S22
F-Cs	Base (KOH)	0.1 M	RRDE	3 mA cm^{-2}	80 %	2020	S23
F-Cs	Acid ($HClO_4$)	0.1 M	RRDE	3.2 mA cm^{-2}	75 %	2020	S23
{001}-Fe_2O_{3-x}	Acid (H_2SO_4)	0.005 M	RRDE	1.2 mA cm^{-2}	99 %	2020	S24
{001}-Fe_2O_{3-x}	Neutral (Na_2SO_4)	0.1 M	RRDE	3.5 mA cm^{-2}	92 %	2020	S24
{001}-Fe_2O_{3-x}	Base (KOH)	0.1 M	RRDE	4.1 mA cm^{-2}	99 %	2020	S24
N-mFLG-8	Base (KOH)	0.1 M	RRDE	3 mA cm^{-2}	95 %	2020	S25
N-CNT	Neutral (PBS)	0.1 M	RRDE	0.4 mA	90 %	2021	S26
Co-N-C	Neutral (NaCl)	0.5 M	RRDE	1.2 mA cm^{-2} (η = 0.5 V)	60 %	2021	S27
Co-N-C	Neutral (NaCl)	0.5 M	GDE	50 mA cm^{-2} (η = 1.2 V)	95 %	2021	S27
Co-N-C	Neutral (NaCl)	0.5 M	GDE	100 mA cm^{-2} (η = 2.2 V)	55 %	2021	S27
Sc-$CoSe_2$	Acid (H_2SO_4)	0.5 M	RRDE	1.2 mA cm^{-2}	95 %	2021	S28
Sc-$CoSe_2$	Acid (H_2SO_4)	0.5 M	GDE	63 mA cm^{-2} (η = 0.7 V)	95 %	2021	S28
Ni-MOF NS-6	Base (KOH)	0.1 M	RRDE	0.6 mA (η = 0.5 V)	95 %	2021	S29
a-TiO_{2-x}/TiC	Base (KOH)	0.1 M	RRDE	0.6 mA (η = 0.4 V)	90 %	2021	S30

O-GOMC	Base (KOH)	0.1 M	RRDE	0.6 mA (η = 0.3 V)	75 %	2021	S31
O-GOMC	Neutral (PBS)	0.1 M	RRDE	0.7 mA (η = 0.5 V)	80 %	2021	S31
P-Co@C-700	Acid ($HClO_4$)	0.1 M	RRDE	3 mA cm^{-2}	82 %	2022	S32
In_2O_3/CDs-10	Base (KOH)	0.1 M	RRDE	2.8 mA cm^{-2} (η = 0.3 V)	92 %	2022	S33
In_2O_3/CDs-10	Base (KOH)	0.1 M	Flow-cell	120 mA cm^{-2} (η = 0.2 V)	95 %	2022	S33
c-Mo/NCPs	Base (KOH)	0.1 M	RRDE	2.8 mA cm^{-2} (η = 0.5 V)	85 %	2022	S34
c-Mo/NCPs	Base (KOH)	0.1 M	Flow-cell	45 mA cm^{-2} (η = 0.4 V)	80 %	2022	S34
$Ni_{2-x}P$-V_{Ni}	Base (KOH)	0.1 M	RRDE	3 mA cm^{-2} (η = 0.5 V)	95 %	2022	S35
$Ni_{2-x}P$-V_{Ni}	Neutral (PBS)	0.1 M	Flow-cell	15 mA cm^{-2}	70 %	2022	S35
Co_1@GO	Base (KOH)	0.1 M	RRDE	0.5 mA (η = 0.3 V)	75 %	2022	S36
OCG-800	Base (KOH)	0.1 M	RRDE	3.7 mA cm^{-2}	92 %	2022	S37
In SAs/NSBC	Neutral (PBS)	0.1 M	RRDE	0.7 mA cm^{-2}	92 %	2022	S38
In SAs/NSBC	Neutral (PBS)	0.1 M	PEMFC	90 mA cm^{-2} (η = N/A)	80 %	2022	S38
Pb(NiWMnNb $ZrTi)_{1/6}O_3$	Base (KOH)	0.1 M	RRDE	3 mA cm^{-2}	92 %	2022	S39
Pb(NiWMnNb $ZrTi)_{1/6}O_3$	Base (KOH)	0.1 M	H-cell	35 mA cm^{-2}	N/A	2022	S39
Co-N_2-C/HO	Base (KOH)	0.1 M	RRDE	2.8 mA cm^{-2}	83 %	2022	S40
Pd*x*-NC	Base (KOH)	0.1 M	RRDE	3.2 mA cm^{-2}	88 %	2022	S41
Pd*x*-NC	Neutral (PBS)	0.1 M	RRDE	3.2 mA cm^{-2}	35 %	2022	S41
Pd*x*-NC	Acid ($HClO_4$)	0.1 M	RRDE	1.8 mA cm^{-2}	75 %	2022	S41
Co_{SA}-N-CNTs	Base (KOH)	0.1 M	RRDE	3 mA cm^{-2}	80 %	2022	S42
Co_{SA}-N-CNTs	Acid (H_2SO_4)	0.5 M	Flow-cell	100 mA (area N/A) (η = 0.7 V)	90 %	2022	S42
CoPC-CNTs	Neutral (K_2SO_4)	1.0 M	Flow-cell	300 mA cm^{-2} (E_{cell} = 2.57 V for 100 hours)	90 %	2023	S59
CoPC-OCNTs	Base (KOH)	1.0 M	Flow-cell	200 mA cm^{-2} (for 30 hours)	95 %	2023	S60
	Neutral (K_2SO_4)	0.3 M	Flow-cell	25~100 mA cm^{-2}	65 ~ 90 %	2023	S60

Supplementary Table 31 | Comparison of 2e-WOR activities of D-PSFZ to recently published articles measured at high η region. [a] $\eta = 0.6$ V indicates where E = ~2.35 V *vs.* RHE since $E^o(H_2O_2/H_2O) = 1.76$ V.

Catalysts	Electrolyte	Ionic strength	Testing condition	J (mA cm^{-2}) at $\eta \sim 0.6$ V [a]	Corresponding FE (%)	Year	Ref.
D-PSFZ	Neutral ($KHCO_3$)	0.1 M	CF (H-cell)	1.5 mA cm^{-2}	36.5 %	*This work*	
D-PSFZ	Neutral ($KHCO_3$)	0.5 M	CF (H-cell)	5 mA cm^{-2}	50.6 %	*This work*	
D-PSFZ	Neutral ($KHCO_3$)	1.0 M	CF (H-cell)	9 mA cm^{-2}	43.1 %	*This work*	
D-PSFZ	Neutral ($KHCO_3$)	2.0 M	CF (H-cell)	20 mA cm^{-2}	40.0 %	*This work*	
D-PSFZ	Weak base ($KHCO_3/K_2CO_3$)	1.0 M	CF (H-cell)	~70 mA cm^{-2} ($\eta = 0.74$ V)	59.6 %	*This work*	
D-PSFZ	Weak base ($KHCO_3/K_2CO_3$)	2.0 M	CF (H-cell)	~50 mA cm^{-2} ($\eta = 0.64$ V)	55.0 %	*This work*	
				~80 mA cm^{-2} ($\eta = 0.84$ V)	63.5 %		
$BiVO_4$	Neutral ($NaHCO_3$)	1.0 M	FTO (H-cell)	2.8 mA cm^{-2} ($\eta = 0.64$ V)	30 %	2017	S43
				5.5 μmol min^{-1} cm^{-2} ($\eta = 1.34$ V)	70 %		
$CaSnO_3$	Neutral ($NaHCO_3$)	2.0 M	FTO (H-cell)	3 mA cm^{-2} ($\eta = 0.64$ V)	45 %	2019	S44
ZnO	Neutral ($KHCO_3$)	2.0 M	FTO (H-cell)	5 mA cm^{-2}	60 %	2019	S45
C,N codoped TiO_2	Weak acid (Na_2SO_4, pH =3)	0.05 M	Ti (H-cell)	0.4 mA cm^{-2} (η ~1.6 V)	7 %	2020	S46
CFP-60%	Base (Na_2CO_3, pH = 12)	1.0 M	CF (H-cell)	~50 mA cm^{-2} ($\eta = 0.64$ V)	~50 %	2020	S47
BDD-Ti	Neutral ($NaHCO_3$)	1.0 M	Ti (H-cell)	11 μmol min^{-1} cm^{-2} ($\eta = 1.44$ V)	~30 %	2020	S48
$CaSnO_3$@CF-2	Neutral ($NaHCO_3$)	2.0 M	CF (H-cell)	~20 mA cm^{-2}	~55 %	2021	S49
(Ti,Mn)O_x	Neutral (PBS)	0.5 M	FTO (H-cell)	~1 mA cm^{-2} ($\eta = 0.4$ V)	~18 %	2021	S50
CNFs/NF@PTFE-60%	Base (Na_2CO_3, pH = 12)	1.0 M	CF (H-cell)	75 mA cm^{-2} ($\eta = 2$ V)	30 %	2021	S51
BDD	Weak base ($KHCO_3/K_2CO_3$)	2.0 M	BDD film (H-cell)	~100 mA cm^{-2} ($\eta = 1$ V)	75 %	2021	S52
FTO	Base (K_2CO_3, pH = 12)	5.0 M	FTO (H-cell)	150 mA cm^{-2} ($\eta \sim 1.4$ V)	80 %	2022	S53
CC/$CuWO_4$	Neutral ($KHCO_3$)	2.0 M	CF (H-cell)	~22 mA cm^{-2} ($\eta = 0.84$ V)	~70 %	2022	S54
BDD/Nb-4	Base (K_2CO_3, pH = 12)	1.0 M	BDD film (H-cell)	100 mA cm^{-2} ($\eta > 1.4$ V)	~70 %	2022	S55
Sb_2O_3	Neutral ($KHCO_3$)	2.0 M	FTO (H-cell)	0.1 μmol min^{-1} cm^{-2} ($\eta = 1.34$ V)	~18 %	2022	S56

Supplementary Table 32 | Concurrent H_2O_2 production electrochemical cells and their performance. [a]The active areas of cathode and anode are different thus the current density (*J*) are shown in J_{anode} and $J_{cathode}$. [b]The FEs are not available. [c]The system is based on the photo-electrocatalysis.

Anode (active area)	Cathode (active area)	Electrolyte	Testing condition	*J* (mA cm^{-2}) at E_{cell} (V)	Corresponding FE (%)	Year	Ref.
D-PSFZ (1 cm^2)	D-PSFZ (1 cm^2)	0.5 M $KHCO_3$	Beaker cell	8 mA cm^{-2} at 2.1 V	155 %	*This work*	
D-PSFZ (1 cm^2)	D-PSFZ (1 cm^2)	1.0 M $KHCO_3$	Beaker cell	10 mA cm^{-2} at 2.1 V	155 %	*This work*	
D-PSFZ (1 cm^2)	D-PSFZ (1 cm^2)	1 M $KHCO_3/K_2CO_3$	Beaker cell	50 mA cm^{-2} at 2.1 V	160 %	*This work*	
CFP-60% (0.42 cm^2)	O-CNT[S5] (2 cm^2)	1.0 M Na_2CO_3	Flow cell	J_{anode}:120 mA cm^{-2} $J_{cathode}$:25 mA cm^{-2} at 1.7 V [a]	150 %	2020	S47
$WO_3/BiVO_4$ (7 cm^2)	Au (14.4 cm^2)	2 M $KHCO_3$	Petri-dish cell	NA (photocatalysts)	[b]0.066 µmol min^{-1} cm^{-2}	2017	S57
$BiVO_4$ (2 cm^2)	CMK-3 (2 cm^2)	Anode / Cathode 1 M Na_2SO_4 / 2 M $KHCO_3$	H cell	[c]5 mA cm^{-2} at 1.5 V	[b]0.48 µmol min^{-1} cm^{-2}	2018	S58
				[c]1 mA cm^{-2} at no bias	[b]2.42 µmol min^{-1} cm^{-2}		

Supplementary References

S1. Siahrostami, S. *et al.* Enabling direct H_2O_2 production through rational electrocatalyst design. *Nat. Mater.* **12**, 1137–1143 (2013).

S2. Zheng, Z., Ng, Y. H., Wang, D. W. & Amal, R. Epitaxial Growth of Au–Pt–Ni Nanorods for Direct High Selectivity H_2O_2 Production. *Adv. Mater.* **28**, 9949–9955 (2016).

S3. Zhao, K. *et al.* Enhanced H_2O_2 production by selective electrochemical reduction of O_2 on fluorine-doped hierarchically porous carbon. *J. Catal.* **357**, 118–126 (2018).

S4. Iglesias, D. *et al.* N-Doped Graphitized Carbon Nanohorns as a Forefront Electrocatalyst in Highly Selective O_2 Reduction to H_2O_2. *Chem* **4**, 106–123 (2018).

S5. Lu, Z. *et al.* High-efficiency oxygen reduction to hydrogen peroxide catalysed by oxidized carbon materials. *Nat. Catal.* **1**, 156–162 (2018).

S6. Chen, S. *et al.* Defective Carbon-Based Materials for the Electrochemical Synthesis of Hydrogen Peroxide. *ACS Sustain. Chem. Eng.* **6**, 311–317 (2018).

S7. Sun, Y. *et al.* Efficient Electrochemical Hydrogen Peroxide Production from Molecular Oxygen on Nitrogen-Doped Mesoporous Carbon Catalysts. *ACS Catal.* **8**, 2844–2856 (2018).

S8. Shen, R. *et al.* High-Concentration Single Atomic Pt Sites on Hollow CuS_x for Selective O_2 Reduction to H_2O_2 in Acid Solution. *Chem* **5**, 2099–2110 (2019).

S9. Sheng, H. *et al.* Electrocatalytic Production of H_2O_2 by Selective Oxygen Reduction Using Earth-Abundant Cobalt Pyrite (CoS_2). *ACS Catal.* **9**, 8433–8442 (2019).

S10. Jiang, K. *et al.* Highly selective oxygen reduction to hydrogen peroxide on transition metal single atom coordination. *Nat. Commun.* **10**, (2019).

S11. Wang, Y. L. *et al.* In Situ Deposition of Pd during Oxygen Reduction Yields Highly Selective and Active

Electrocatalysts for Direct H_2O_2 Production. *ACS Catal.* **9**, 8453–8463 (2019).

S12. Han, L. *et al.* In-Plane Carbon Lattice-Defect Regulating Electrochemical Oxygen Reduction to Hydrogen Peroxide Production over Nitrogen-Doped Graphene. *ACS Catal.* **9**, 1283–1288 (2019).

S13. Hasanzadeh, A., Khataee, A., Zarei, M. & Zhang, Y. Two-electron oxygen reduction on fullerene C_{60}-carbon nanotubes covalent hybrid as a metal-free electrocatalyst. *Sci. Rep.* **9**, 1–13 (2019).

S14. Sun, Y. *et al.* Activity-Selectivity Trends in the Electrochemical Production of Hydrogen Peroxide over Single-Site Metal-Nitrogen-Carbon Catalysts. *J. Am. Chem. Soc.* **141**, 12372–12381 (2019).

S15. Yang, Q. *et al.* Atomically dispersed Lewis acid sites boost 2-electron oxygen reduction activity of carbon-based catalysts. *Nat. Commun.* **11**, 1–10 (2020).

S16. Chang, Q. *et al.* Promoting H_2O_2 production via 2-electron oxygen reduction by coordinating partially oxidized Pd with defect carbon. *Nat. Commun.* **11**, 1–9 (2020).

S17. Pang, Y. *et al.* Mesoporous Carbon Hollow Spheres as Efficient Electrocatalysts for Oxygen Reduction to Hydrogen Peroxide in Neutral Electrolytes. *ACS Catal.* **10**, 7434–7442 (2020).

S18. Jung, E. *et al.* Atomic-level tuning of Co–N–C catalyst for high-performance electrochemical H_2O_2 production. *Nat. Mater.* **19**, 436–442 (2020).

S19. Zhang, Q. *et al.* Direct insights into the role of epoxy groups on cobalt sites for acidic H_2O_2 production. *Nat. Commun.* **11**, (2020).

S20. Wang, M. *et al.* Partially Pyrolyzed Binary Metal–Organic Framework Nanosheets for Efficient Electrochemical Hydrogen Peroxide Synthesis. *Angew. Chem. Int. Ed.* **59**, 14373–14377 (2020).

S21. Li, H. *et al.* Scalable neutral H_2O_2 electrosynthesis by platinum diphosphide nanocrystals by regulating oxygen reduction reaction pathways. *Nat. Commun.* **11**, 3928 (2020).

S22. Gao, J. *et al.* Enabling Direct H_2O_2 Production in Acidic Media through Rational Design of Transition Metal Single Atom Catalyst. *Chem* **6**, 658–674 (2020).

S23. Jia, N. *et al.* N,F-Codoped Carbon Nanocages: An Efficient Electrocatalyst for Hydrogen Peroxide Electroproduction in Alkaline and Acidic Solutions. *ACS Sustain. Chem. Eng.* **8**, 2883–2891 (2020).

S24. Gao, R. *et al.* Engineering Facets and Oxygen Vacancies over Hematite Single Crystal for Intensified Electrocatalytic H_2O_2 Production. *Adv. Funct. Mater.* **30**, 1910539 (2020).

S25. Li, L. *et al.* Tailoring Selectivity of Electrochemical Hydrogen Peroxide Generation by Tunable Pyrrolic-Nitrogen-Carbon. *Adv. Energy Mater.* **10**, 2000789 (2020).

S26. Ren, S., Cui, W., Li, L. & Yi, Z. N-doped carbon nanotubes as an efficient electrocatalyst for O_2 conversion to H_2O_2 in neutral electrolyte. *Sustain. Energy Fuels* **5**, 6310–6314 (2021).

S27. Zhao, Q. *et al.* Approaching a high-rate and sustainable production of hydrogen peroxide: oxygen reduction on Co–N–C single-atom electrocatalysts in simulated seawater. *Energy Environ. Sci.* **14**, 5444–5456 (2021).

S28. Zhang, X. L. *et al.* Strongly Coupled Cobalt Diselenide Monolayers for Selective Electrocatalytic Oxygen Reduction to H_2O_2 under Acidic Conditions. *Angew. Chem. Int. Ed.* **60**, 26922–26931 (2021).

S29. Wang, M. *et al.* An Efficient Interfacial Synthesis of Two-Dimensional Metal–Organic Framework Nanosheets for Electrochemical Hydrogen Peroxide Production. *Angew. Chem. Int. Ed.* **60**, 11190–11195 (2021).

S30. Xu, Z. *et al.* Enhanced Electrochemical H_2O_2 Production via Two-Electron Oxygen Reduction Enabled by Surface-Derived Amorphous Oxygen-Deficient TiO_{2-x}. *ACS Appl. Mater. Interfaces* **13**, 33182–33187 (2021).

S31. Lim, J. S. *et al.* Designing highly active nanoporous carbon H_2O_2 production electrocatalysts through active site identification. *Chem* **7**, 3114–3130 (2021).

S32. Wang, Y., Zhou, Y., Feng, Y. & Yu, X. Y. Synergistic Electronic and Pore Structure Modulation in Open Carbon Nanocages Enabling Efficient Electrocatalytic Production of H_2O_2 in Acidic Medium. *Adv. Funct. Mater.* **32**, 2110734 (2022).

S33. Wu, J. *et al.* The Electron Transport Regulation in Carbon Dots/In_2O_3 Electrocatalyst Enable 100% Selectivity for Oxygen Reduction to Hydrogen Peroxide. *Adv. Funct. Mater.* **32**, 2203647 (2022).

S34. Jin, M. *et al.* Low-Coordinated Mo Clusters for High-Efficiency Electrocatalytic Hydrogen Peroxide Production. *Adv. Mater. Interfaces* **2201144**, 2201144 (2022).

S35. Zhou, Z. *et al.* Cation-Vacancy-Enriched Nickel Phosphide for Efficient Electrosynthesis of Hydrogen Peroxides. *Adv. Mater.* **34**, 2106541 (2022).

S36. Zhang, B. W. *et al.* Highly efficient and selective electrocatalytic hydrogen peroxide production on Co-O-C active centers on graphene oxide. *Commun. Chem.* **5**, 1–7 (2022).

S37. Lee, K. *et al.* Structure-controlled graphene electrocatalysts for high-performance H2O2 production. *Energy Environ. Sci.* **15**, 2858–2866 (2022).

S38. Zhang, E. *et al.* Engineering the Local Atomic Environments of Indium Single-Atom Catalysts for Efficient Electrochemical Production of Hydrogen Peroxide. *Angew. Chem. Int. Ed.* **61**, e202117347 (2022).

S39. Chen, Z. *et al.* Entropy Enhanced Perovskite Oxide Ceramic for Efficient Electrochemical Reduction of Oxygen to Hydrogen Peroxide. *Angew. Chemie Int. Ed.* **61**, e202200086 (2022).

S40. Gong, H. *et al.* Low-Coordinated Co-N-C on Oxygenated Graphene for Efficient Electrocatalytic H_2O_2 Production. *Adv. Funct. Mater.* **32**, 2106886 (2022).

S41. Wang, N. *et al.* Highly Selective Oxygen Reduction to Hydrogen Peroxide on a Carbon-Supported Single-Atom Pd Electrocatalyst. *ACS Catal.* **12**, 4156–4164 (2022).

S42. Liu, W. *et al.* Tuning the atomic configuration of Co-N-C electrocatalyst enables highly-selective H2O2 production in acidic media. *Appl. Catal. B Environ.* **310**, 121312 (2022).

S43. Shi, X. *et al.* Understanding activity trends in electrochemical water oxidation to form hydrogen peroxide. *Nat. Commun.* **8**, 701 (2017).

S44. Park, S. Y. *et al.* CaSnO3 : An Electrocatalyst for Two-Electron Water Oxidation Reaction to Form H_2O_2. *ACS Energy Lett.* **4**, 352–357 (2019).

S45. Kelly, S. R. *et al.* ZnO As an Active and Selective Catalyst for Electrochemical Water Oxidation to Hydrogen Peroxide. *ACS Catal.* **9**, 4593–4599 (2019).

S46. Xue, S. G. *et al.* Selective Electrocatalytic Water Oxidation to Produce H2O2 Using a C,N Codoped TiO2 Electrode in an Acidic Electrolyte. *ACS Appl. Mater. Interfaces* **12**, 4423–4431 (2020).

S47. Xia, C. *et al.* Confined local oxygen gas promotes electrochemical water oxidation to hydrogen peroxide. *Nat. Catal.* **3**, 125–134 (2020).

S48. Mavrikis, S., Göltz, M., Rosiwal, S., Wang, L. & Ponce De León, C. Boron-Doped Diamond Electrocatalyst for Enhanced Anodic H2O2Production. *ACS Appl. Energy Mater.* **3**, 3169–3173 (2020).

S49. Zhang, C. *et al.* High Yield Electrosynthesis of Hydrogen Peroxide from Water Using Electrospun $CaSnO_3$@Carbon Fiber Membrane Catalysts with Abundant Oxygen Vacancy. *Adv. Funct. Mater.* **31**, 2100099 (2021).

S50. Li, J. *et al.* Microstructural origin of selective water oxidation to hydrogen peroxide at low overpotentials: a study on Mn-alloyed TiO_2. *J. Mater. Chem. A* **9**, 18498–18505 (2021).

S51. Sun, Y. *et al.* Efficient electrochemical water oxidation to hydrogen peroxide over intrinsic carbon defect-rich carbon nanofibers. *J. Mater. Chem. A* **9**, 23994–24001 (2021).

S52. Mavrikis, S. *et al.* Effective Hydrogen Peroxide Production from Electrochemical Water Oxidation. *ACS Energy Lett.* **6**, 2369–2377 (2021).

S53. Fan, L. *et al.* CO_2/carbonate-mediated electrochemical water oxidation to hydrogen peroxide. *Nat. Commun.* **13**, 1–9 (2022).

S54. Li, L., Xiao, K., Wong, P. K., Hu, Z. & Yu, J. C. Hydrogen Peroxide Production from Water Oxidation on a $CuWO_4$ Anode in Oxygen-Deficient Conditions for Water Decontamination. *ACS Appl. Mater. Interfaces* **14**, 7878–7887 (2022).

S55. Mavrikis, S., Göltz, M., Rosiwal, S., Wang, L. & Ponce de León, C. Carbonate-Induced Electrosynthesis of Hydrogen Peroxide via Two-Electron Water Oxidation. *ChemSusChem* **15**, e202102137 (2022).

S56. Guo, W. *et al.* Effects of Sb_2O_3 polymorphism on the performances for electrocatalytic H_2O_2 production via the two-electron water oxidation reaction. *Appl. Surf. Sci.* **606**, 155006 (2022).

S57. Fuku, K. *et al.* Photoelectrochemical Hydrogen Peroxide Production from Water on a $WO_3/BiVO_4$ Photoanode and from O_2 on an Au Cathode Without External Bias. *Chem. - An Asian J.* **12**, 1111–1119 (2017).

S58. Shi, X., Zhang, Y., Siahrostami, S. & Zheng, X. Light-Driven $BiVO_4$ –C Fuel Cell with Simultaneous Production of H_2O_2. *Adv. Energy Mater.* **8**, 1801158 (2018).

S59 Lee, B.-H. *et. al,* Supramolecular tuning of supported metal phthalocyanine catalysts for hydrogen peroxide electrosynthesis. *Nat. Catal.*, **6**, 234 (2023).

S60 Cao, P., Quan, X., Nie, X., Zhao, K., Liu, Y., Chen, S., Yu, H., Chen, J. G., Metal single-site catalyst design for electrocatalytic production of hydrogen peroxide at industrial-relevant currents. *Nat. Commun.*, **14**, 172 (2023).
