# Supplementary material for: Concurrent oxygen reduction and water oxidation at high ionic strength for scalable electrosynthesis of hydrogen peroxide
Source: Nat Commun. 2023 Sep 19;14:5822. doi: 10.1038/s41467-023-41397-1 (PMC10509222; doi:10.1038/s41467-023-41397-1)
Supplement: Supplementary file 3 — Description of Additional Supplementary Files [file 41467_2023_41397_MOESM3_ESM.pdf]

<u>**Description of Additional Supplementary Files**</u>

Supplementary Movie 1. *in-situ* Dye decomposition experiments by H_2O_2 from the D-PSFZ || D-PSFZ cell.
